# Heterologous infection and vaccination shapes immunity against SARS-CoV-2 variants

**DOI:** 10.1126/science.abm0811

**Published:** 2022-01-14

**Authors:** Catherine J. Reynolds, Joseph M. Gibbons, Corinna Pade, Kai-Min Lin, Diana Muñoz Sandoval, Franziska Pieper, David K. Butler, Siyi Liu, Ashley D. Otter, George Joy, Katia Menacho, Marianna Fontana, Angelique Smit, Beatrix Kele, Teresa Cutino-Moguel, Mala K. Maini, Mahdad Noursadeghi, COVIDsortium Immune Correlates Network, Tim Brooks, Amanda Semper, Charlotte Manisty, Thomas A. Treibel, James C. Moon, COVIDsortium Investigators, Áine McKnight, Daniel M. Altmann, Rosemary J. Boyton

**Affiliations:** 1 Department of Infectious Disease, Imperial College London, London, UK.; 2 Blizard Institute, Barts and the London School of Medicine and Dentistry, Queen Mary University of London, London, UK.; 3 UK Health Security Agency, Porton Down, UK.; 4 St. Bartholomew’s Hospital, Barts Health NHS Trust, London, UK.; 5 Royal Free London NHS Foundation Trust, London, UK.; 6 Division of Infection and Immunity, University College London, London, UK.; 7 Institute of Cardiovascular Science, University College London, London, UK.; 8 Department of Immunology and Inflammation, Imperial College London, London, UK.; 9 Lung Division, Royal Brompton and Harefield Hospitals, Guy’s and St. Thomas’ NHS Foundation Trust, London, UK.

## Abstract

The impact of the initial severe acute respiratory syndrome coronavirus 2 (SARS-CoV-2) infecting strain on downstream immunity to heterologous variants of concern (VOCs) is unknown. Studying a longitudinal healthcare worker cohort, we found that after three antigen exposures (infection plus two vaccine doses), S1 antibody, memory B cells, and heterologous neutralization of B.1.351, P.1, and B.1.617.2 plateaued, whereas B.1.1.7 neutralization and spike T cell responses increased. Serology using the Wuhan Hu-1 spike receptor binding domain poorly predicted neutralizing immunity against VOCs. Neutralization potency against VOCs changed with heterologous virus encounter and number of antigen exposures. Neutralization potency fell differentially depending on targeted VOCs over the 5 months from the second vaccine dose. Heterologous combinations of spike encountered during infection and vaccination shape subsequent cross-protection against VOC, with implications for future-proof next-generation vaccines.

## Immune imprinting

For severe acute respiratory syndrome coronavirus 2 (SARS-CoV-2), immune responses to heterologous variants are influenced by a person’s infection history. Healthcare workers (HCWs) may be exposed to several doses and types of antigens, either by natural infection or by vaccination. Reynolds *et al*. studied a cohort of UK HCWs followed since March 2020. The immunological profiles of these people depended on how often the subject had encountered antigen and which variant was involved. Vaccine responses after infection were found to be less effective if the infection involved heterologous spike from a variant virus. Unfortunately, the N501Y spike mutation, found in many variants, seems to induce the regulatory T cell transcription factor FOXP3, indicating that the virus could subvert effective T cell function. Changes to antibody binding between variants also means that serology data using the Wuhan Hu-1 S1 receptor-binding domain sequence may not be a reliable measure of protection. —CA

## Teaser

SARS-CoV-2 B.1.1.7 infection differentially shapes heterologous immunity against subsequent variants in two-dose vaccinees.

After experiencing >18 months of the COVID-19 pandemic, immunological analysis has shifted to issues of response durability, boosting, and mitigation against future variants of concern (VOCs). Researchers thus are confronting viral and population heterogeneities that affect immunity and future protection (*1*–*4*). Individuals are either immunologically naïve to severe acute respiratory syndrome coronavirus 2 (SARS-CoV-2) or have been infected with either the original Wuhan Hu-1 strain or one of the Alpha to Delta VOCs, which we will refer to by full Pango lineage terms. Infection-naïve individuals, those infected by ancestral SARS-CoV-2 Wuhan Hu-1 or a VOC, may have received different numbers of vaccine doses. Thus, there is a spectrum of individuals spanning from the immunologically naïve to those who have experienced one, two, three exposures (and four with boosting) to homologous or heterologous spike sequences. The challenge is to understand whether different antigen exposure combinations are associated with the same quality, quantity, and durability of immunity and ability to cross-protect against other VOCs.

Using a cohort of UK healthcare workers (HCWs) for whom we have extensive, longitudinal, clinical, transcriptomic, and immunologic characterization (*5*–*10*)*,* we address these questions by immunological comparison of BNT162b2 (Pfizer-BioNTech vaccine) vaccinees, with or without infection. We and others have shown the boosting effect of prior infection by the Wuhan Hu-1 strain on the response to vaccination with homologous spike (*8*, *9*, *11*–*14*), and others are decoding changes in immune programming between the first and second dose (*15*)*.* We compared the impact of prior infection with Wuhan Hu-1 or B.1.1.7. (Alpha VOC) in the context of vaccination on T and memory B cell (MBC) responses, cross-neutralization against VOCs, durability of immunity, and susceptibility to B.1.617.2 (Delta VOC) breakthrough infection. We considered the extent to which first encounter with the spike sequence shapes subsequent response features to explore the possible effect of immune imprinting or “original antigenic sin” (*16*–*18*).

## B and T cell immunity after three homologous antigen exposures

**Fig. 1.**
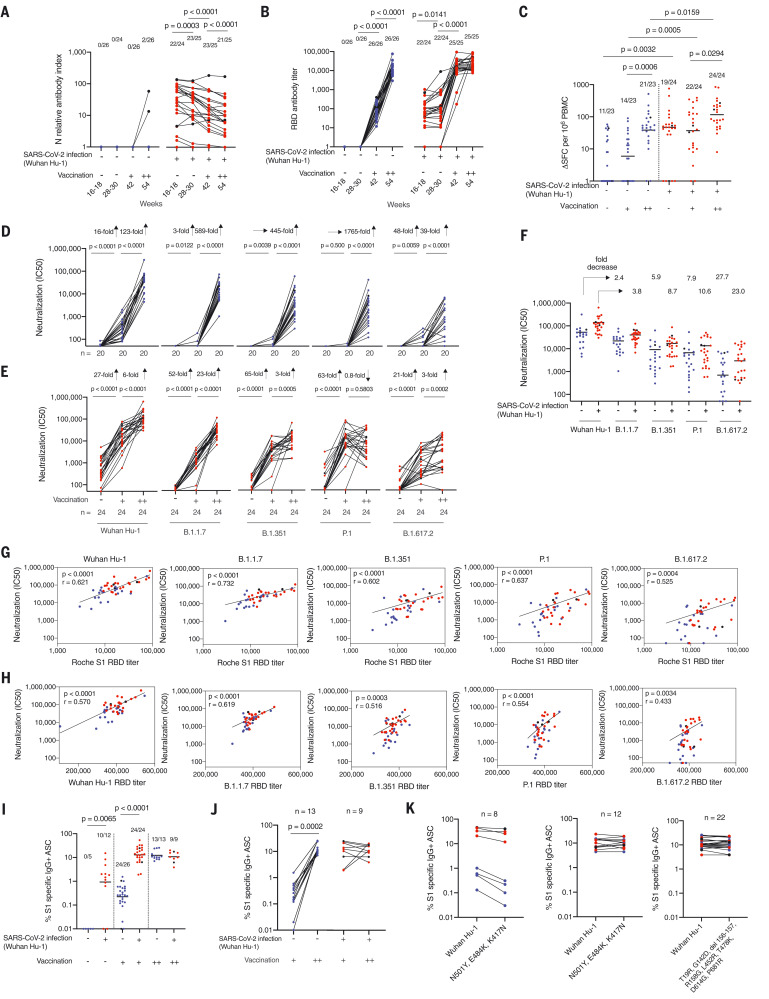
T and B cell immunity to infection with Wuhan Hu-1 SARS-CoV-2 and after one and two doses of BNT162b2 vaccine in infection naïve and previously infected HCWs. (**A**) N Ab and (**B**) S1 RBD serum Ab titers measured by ECLIA in HCWs with (red, *n* = 25) and without (blue, *n* = 26) laboratory-confirmed SARS-CoV-2 infection at 16 to 18 weeks after the start of the first UK epidemic wave and 2 to 3 weeks after first (+, 42 weeks) and second (++, 54 weeks) dose of BNT162b2 vaccine. In all figures, HCWs without laboratory-confirmed infection are shown in blue and HCWs with laboratory-confirmed infection are shown in red. HCWs with new infection or reinfection after 30 weeks are shown in black. (**C**) Magnitude of T cell responses to spike MEP pool in HCWs with (red, *n* = 24) and without (blue, *n* = 23) laboratory-confirmed SARS-CoV-2 infection 16 to 18 weeks after the start of the first UK epidemic wave and 2 to 3 weeks after first (+, 42 weeks) and second (++, 54 weeks) dose of vaccine. Neutralizing Ab titer (IC_50_) against authentic Wuhan Hu-1 live virus and B.1.1.7, B.1.351, P.1, and B.1.617.2 VOCs is plotted longitudinally for (**D**) SARS-CoV-2 infection naïve (blue, *n* = 20) and (**E**) previously Wuhan Hu-1 infected HCWs (red, *n* = 24) unvaccinated and after the first and second dose of BNT162b2 vaccine. (**F**) IC_50_ nAb against Wuhan Hu-1 and VOCs after two-dose BNT162b2 vaccination in previously infected HCWs (red, *n* = 24) and infection-naïve HCWs (blue, *n* = 20). (**G**) Correlation between Roche S1 RBD Ab titer and nAb (IC_50_) against SARS-CoV-2 authentic Wuhan Hu-1 live virus and the B.1.1.7, B.1.351, P.1, and B.1.617.2 VOCs. (**H**) Correlation between Wuhan Hu-1, VOC RBD, and corresponding live virus in two-dose BNT162b2 vaccinated previously infected (red, *n* = 24) and SARS-CoV-2 infection-naïve (blue, *n* = 20) HCWs. (**I**) Percentage of S1-specific IgG^+^ antibody-secreting cells (ASCs) in SARS-CoV-2 infection naïve and previously infected HCWs at 16 to 18 weeks and after first- and second-dose BNT162b2 vaccination. (**J**) Percentage of S1-specific IgG^+^ ASCs plotted pairwise after first- and second-dose vaccination for HCWs with (red, *n* = 9) and without (blue, *n* = 13) prior SARS-CoV-2 infection. (**K**) Left, percentage of IgG^+^ ASCs specific for Wuhan Hu-1 S1 protein or spike protein containing the mutations N501Y, E484K, and K417N in HCWs with (red) and without (blue) prior SARS-CoV-2 infection after first-dose vaccination (*n* = 4 per group). Middle and right panels, percentage of IgG^+^ ASCs specific for Wuhan Hu-1 S1 or S1 spike protein containing N501Y, E484K, and K417N (B.1.351 VOC) mutations or S1 spike protein containing T19R, G142D, del 156-157, R158G, L452R, T478K, D614G, and P681R (B.1.617.2 VOC) mutations after second-dose BNT162b2 vaccination (middle panel, *n* = 6 per group; right panel, infection naïve HCWs are indicated in blue, *n* = 13, and previously Wuhan Hu-1 infected HCWs are indicated in red, *n* = 9). In (A), (B), (D), (E), and (H), Wilcoxon matched-pairs signed rank test was used; in (C) and (I), Mann-Whitney *U* test was used; and in (G), Spearman’s rank correlation was used. New infection (in the infection-naïve group, *n* = 2) and reinfection (in the previously infected group, *n* = 2) data points shown in black were excluded from statistical analysis. SFCs, spot-forming cells.

We analyzed this longitudinal vaccine cohort (*n* = 51) at 20 d [interquartile range (IQR) = 7] after receiving a second BNT162b2 dose (fig. S1 and table S1). Twenty-five HCWs were infected with SARS-CoV-2 approximately synchronously and coincidentally with peak transmission in London in March 2020 (6). At 1 year, nucleoprotein (N) antibody (Ab) responses had waned but were still positive (anti-N Ab levels expressed as a cutoff index of ≥1.0 were classified as positive), 16% having fallen to subthreshold levels (Fig. 1A). Two (2/25, 8%) HCWs appeared to have been reinfected (increase in N) and two (2/26, 8%) newly infected HCWs (positive N having been previously negative) were among the previously uninfected cohort (Fig. 1A). T cell responses to N were sustained at 1 year in most previously infected HCWs (22/24, 92%) (fig. S2). All previously infected HCWs with positive anti–S1 receptor binding domain (RBD) responses at 16 to 18 weeks had sustained responses at 28 to 30 weeks (22/24, 92%), which substantially increased after single-dose vaccination (36-fold increase in Ab titer) (Fig. 1B). This response plateaued (i.e., only increased a further 1.4-fold) after second-dose vaccination (third antigen exposure). The previously infected HCWs (2/24, 8%) with unrecordable anti–S1 RBD responses at 16 to 18 and 28 to 30 weeks showed a 155-fold increase in anti-S1 response after second-dose vaccination, demonstrating the benefit of a third antigen exposure in poor Ab responders (*19*, *20*)*.* Infection-naïve HCWs had incrementally increased Ab responses after first- and second-dose vaccination, achieving about half the Ab titer of their previously infected counterparts 20 days after their second vaccine dose (Fig. 1B).

T cell responses to the spike mapped epitope peptide (MEP) pool (table S2) increased after second-dose vaccination in previously infected individuals (i.e., third exposure to antigen) (*P* = 0.0294; Fig. 1C). T cell responses after second-dose vaccination in infection-naïve HCWs achieved similar levels to those seen at 16 to 18 weeks after natural SARS-CoV-2 infection. However, there were fewer nonresponders (2/23, 9%) in double-vaccinated infection-naïve individuals compared with (5/25, 20%) 16 to 18 weeks after natural infection.

Infection-naïve individuals showed incremental increases in neutralizing Ab (nAb) to authentic Wuhan Hu-1 virus and VOCs after the first and second vaccination, with the largest fold increase occurring after the second vaccine dose (Wuhan Hu-1,123-fold; B.1.1.7, 589-fold; B.1.351, 445-fold; P.1, 1765-fold; and B.1.617.2, 39-fold). There was a wider heterogeneity of nAb response to B.1.351, P.1, and B.1.617.2 VOCs compared with B.1.1.7 and Wuhan Hu-1 (Fig. 1D). Individuals who had experienced SARS-CoV-2 infection before vaccination also showed the largest fold nAb increase on second antigen exposure (i.e., after the first vaccine dose) (Wuhan Hu-1, 27-fold; B.1.1.7, 52-fold; B.1.351, 65-fold; P.1, 63-fold; and B.1.617.2, 21-fold) (Fig. 1E). By contrast, nAb responses against Wuhan Hu-1 and VOCs B.1.351, P.1, and B.1.617.2 plateaued or decreased between the second and third antigen exposure. This was not the case for nAb responses against Wuhan Hu-1 and B.1.1.7, which increased sixfold and 23-fold, respectively, between the second and third antigen exposure (Fig. 1E). The potency of nAb response after the third antigen exposure varied depending on the VOC being neutralized. Previously infected HCWs who had a low nAb IC_50_ after the first vaccine dose caught up after the second vaccine dose (i.e., the third antigen exposure). Heterologous neutralizing IC_50_s were lower than those against homologous Wuhan Hu-1, and were at risk of falling below a threshold for protection as levels waned (*21*, *22*) (1F, S3).

There was a positive correlation between S1 RBD-binding Ab and neutralization (IC_50_), with previously infected individuals showing higher nAb IC_50_ and S1 RBD binding (Fig. 1G). Wuhan-Hu-1 sequence–specific S1 RBD binding correlated less well with nAb IC_50_ against VOCs B.1.351, P.1, and B.1.617.2, especially for infection-naïve, double-vaccinated HCWs. To establish whether this was due to different sequences in the VOC RBD, the relationship between VOC RBD-binding titers and neutralization was explored. There was positive correlation between the Roche S1 RBD and VOC RBD specific binding (fig. S4). The weaker correlations between VOC nAb titers and VOC RBD binding indicate that antibodies targeting regions outside of the RBD may contribute to neutralization, so the binding titer is not predictive of neutralization (Fig. 1H). For example, a two-dose-vaccinated HCW with an S1 RBD (Wuhan Hu-1)–binding titer of 2950 U/ml, considered strongly positive, showed divergent neutralization of VOCs, with IC_50_s of Wuhan Hu-1, 6,071, B.1.1.7, 1,037; B.1.351, 301; P.1, 560, and B.1.617.2 zero 20 d after second-dose vaccination that all fell to undetectable levels 18 weeks later. This individual was later infected by B.1.617.2. Thus, S1 serology data using the Wuhan Hu-1 S1 RBD and VOC sequence is an unreliable marker for neutralization potency against VOCs.

For infection-naïve subjects, the frequency of S1-specific MBC 20 d after first-dose vaccination was lower compared with 16 to 18 weeks after infection. Conversely, MBCs were amplified in previously infected HCWs to a level similar to that seen after two doses of vaccine (Fig. 1I). As expected from the S1 RBD-binding Ab and nAb responses, MBC responses plateaued, with no further enhancement after a third antigen exposure (i.e., double-vaccinated previously infected HCWs) (Fig. 1, I and J). For infection-naïve HCWs, the MBC frequency for specificity to S1 containing the N501Y, E484K, and K417N B.1.351 mutations was lower after one vaccine dose (Fig. 1K, left panel). After two (two-dose vaccination or single-dose vaccination and prior infection) or three (two-dose vaccination and prior infection) antigen exposures, the MBC response was maintained whether stimulated by S1 or S1-containing mutations found in B.1.351 and B.1.617.2 (Fig. 1K, middle and right panels). There was a high frequency of MBCs able to recognize S1 and S1-containing VOC-specific mutations, but this did not always correlate well with a VOC-specific nAb response (fig. S5). Authentic P.1 (*r* = 0.529, *P* = 0.0138) and B.1.617.2 (*r* = 0.548, *P* = 0.0102) nAb responses of double-vaccinated HCWs with and without prior Wuhan Hu-1 infection positively correlated with the MBC frequency against S1 containing the B.1.617.2–specific mutations (fig. S5C).

**Fig. 2.**
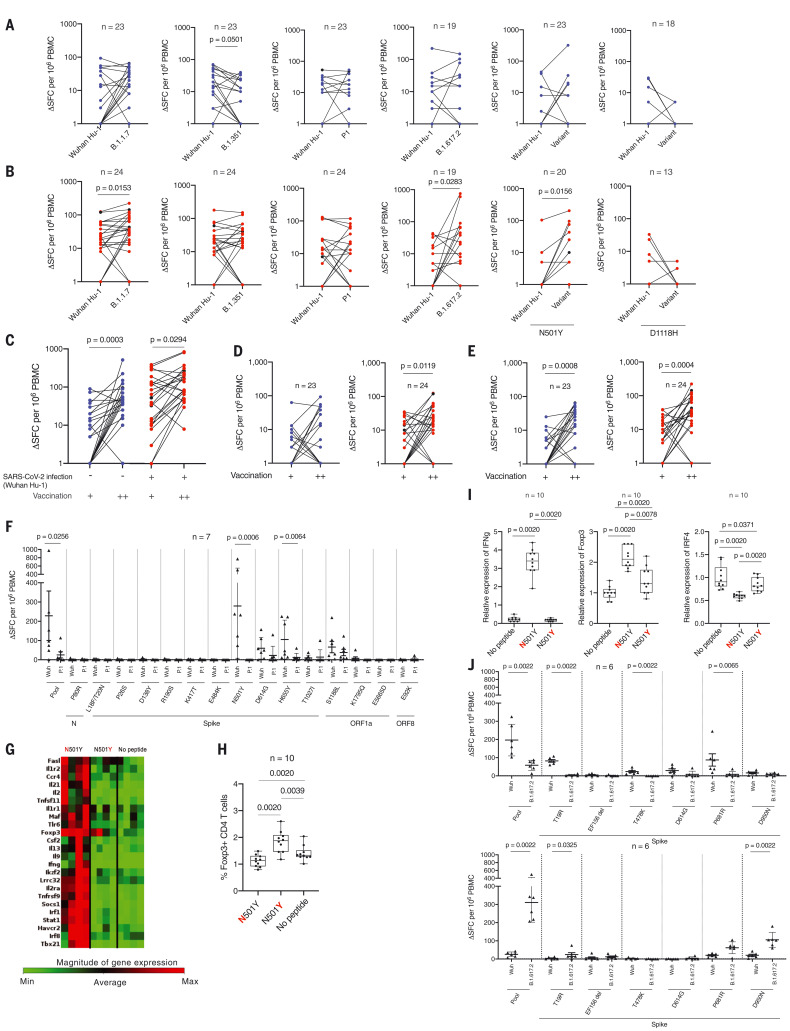
T cell responses to Wuhan Hu-1 and VOC peptide pools after one- and two-dose BNT162b2 vaccination in infection-naïve and previously infected HCWs. Magnitude of T cell responses to B.1.1.7, B.1.351, P.1, and B.1.617.2 variant specific peptide pools and to the matched sequence peptide pools from Wuhan Hu-1 as well as to individual wild-type and variant N501Y and D1118H peptides in (**A**) SARS-Cov-2 infection-naïve (blue) HCWs and (**B**) previously infected (red) HCWs who had received two doses of BNT162b2 vaccine. In all figures, HCWs without labaoratory-confirmed infection are shown in blue, HCWs with laboratory-confirmed infection are shown in red, and HCWs with new infection or reinfection after 30 weeks are shown in black. (**C** to **E**) Magnitude of T cell responses to spike MEP (C), Wuhan Hu-1 (D), and B.1.1.7 variant (E) peptide pools after one (+) and two (++) doses of BNT162b2 vaccine in infection-naïve HCWs (blue) and previously infected HCWs (red). (**F**) Magnitude of T cell response to wild-type Wuhan Hu-1 and P.1 variant peptide pools and individual peptides in Wuhan Hu-1 peptide pool immunized HLA-DRB1*04:01 transgenic mice (*n* = 7). (**G**) Heatmap showing relative gene expression of T cell activation markers in draining lymph node (DLN) cells from Wuhan Hu-1 N501Y peptide–primed DRB1*04:01 transgenic mice (*n* = 4) stimulated for 24 h in vitro with 10 μg/ml of wild-type Wuhan Hu–1 or variant peptide. Genes shown in red are significantly different (*P* < 0.05) between no peptide control and Wuhan Hu-1 or variant peptide stimulated cells with a fold change greater than 1.5. (**H**) Percentage of Foxp3^+^ CD4 T cells by flow cytometry in DLN cells from Wuhan Hu-1 N501Y peptide-primed DRB1*04:01 transgenic mice (*n* = 10) stimulated for 24 h in vitro with 10 mg/ml of wild-type or variant peptide. (**I**) Relative gene expression of *ifng, foxp3*, and *irf4* in DLN cells from Wuhan Hu-1 N501Y peptide-primed DRB1*0401 transgenic mice (*n* = 10) stimulated for 24 h in vitro with 10 μg/ml of Wuhan Hu-1 or variant peptide. (**J**) Magnitude of T cell response to wild-type Wuhan Hu-1 and B.1.617.2 variant peptide pools and individual peptides in Wuhan Hu-1 peptide pool immunized (top panel, *n* = 6) or B.1.617.2 peptide pool immunized (bottom panel, *n* = 6) HLA-DRB1*04:01 transgenic mice. In (A) to (E), (I), and (J), Wilcoxon matched-pairs signed rank test was used. New infection (in the infection-naïve group, *n* = 2) and reinfection (in the previously infected group, *n* = 2) data points shown in black were excluded from statistical analysis. In (F) and (G), Mann-Whitney *U* test was used.

Next, we examined the T cell response against VOCs B.1.1.7, B.1.351, P.1, and B.1.617.2 peptide pools and Wuhan Hu-1 matched pools and individual peptides with substituted epitopes covering N501Y and D1118H (table S2 and Fig. 2, A and B). T cell responses in doubly vaccinated infection-naïve individuals were variable. Previously infected double-vaccinated individuals showed a significantly increased T cell response to B.1.1.7 (*P* = 0.0153) and B.1.617.2 (*P* = 0.0283) peptide pools and N501Y (*P* = 0.0156) variant peptide compared with the respective Wuhan Hu-1 pools and peptide (Fig. 2B). A cumulative increase in T cell response to the Wuhan Hu-1 spike MEP pool was observed as the number of antigen exposures increased (one to two exposures, *P* = 0.0003; two to three exposures, *P* = 0.0294; Fig. 2C). There was a similarly increased T cell response to the B.1.1.7 variant peptide pool (one to two exposures, *P* = 0.0008; two to three exposures, *P* = 0.0004; Fig. 2E) depending on number of antigen exposures, indicating a heteroclitic response to variant peptides (*23*) (Fig. 2, D and E).

## VOC mutation alters T cell effector program

Immunization of transgenic mice expressing human HLADRB1*0401 were used to determine whether any of the variant peptides behave as an altered peptide ligand, eliciting a differential T cell program (*9*). In silico analysis of P.1 variant peptides indicated that most are not predicted to bind to common HLAII alleles found in the UK population (table S3). The N501Y mutation is predicted to change from strong to weak binding to HLADRB1*0401. Interferon γ (IFNγ) T cell responses in mice primed with Wuhan-Hu-1 peptide pool were ablated for the 501Y variant peptide (found in the B.1.1.7, B.1.351, and P.1 VOCs (Fig. 2F) when presented with HLA-DRB1*0401 (*n* = 7, *P* = 0.006). Altered CD4 recognition of the N501Y mutation merits further attention because it is a frequent replacement among reported sequences, comprising a convergent mutational signature shared by the Alpha, Beta, and Gamma lineages (*24*–*26*)*.* Thus far, the lack of T cell responses to 501Y has been described in terms of absence of CD8 recognition (*27*)*.* Our transcriptomic analysis showed an absence of immune effector response to the variant peptide, although induction of the regulatory T cell transcription factor FOXP3 did occur, indicating that the variant peptide may exert an altered peptide ligand effect on T cell function, switching from effector to regulatory (Fig. 2G). This was further confirmed by quantitative polymerase chain reaction (qPCR) for IRF4 and by CD4 expression of FoxP3 protein, indicating that mutant epitopes may subvert T cell activation into a regulatory program (Fig. 2, G to I).

Mice were primed with either B.1.617.2 or Wuhan Hu-1 matched specific peptide pools. In each case, T cells responded on challenge to the pool with which they were primed and not to the heterologous panel (*P* = 0.0022; Fig. 2J). That is, the T cell repertoire distinguishes VOC mutations, specifically recognizing the B.1.617.2 epitopes. In silico analysis of the B.1.617.2 variant peptides indicated that most are predicted to bind one or more of the common HLAII alleles found in the UK population (table S4). The D950N mutation is predicted to change from HLADRB1*0401 binding from weak to strong. T cell responses after priming with B.1.617.2 peptide pool were present for the D950N variant peptide but not after priming with the Wuhan Hu-1 pool (*P* = 0.0022; Fig. 2J).

## Heterologous B.1.1.7 infection shapes subsequent immunity

**Fig. 3.**
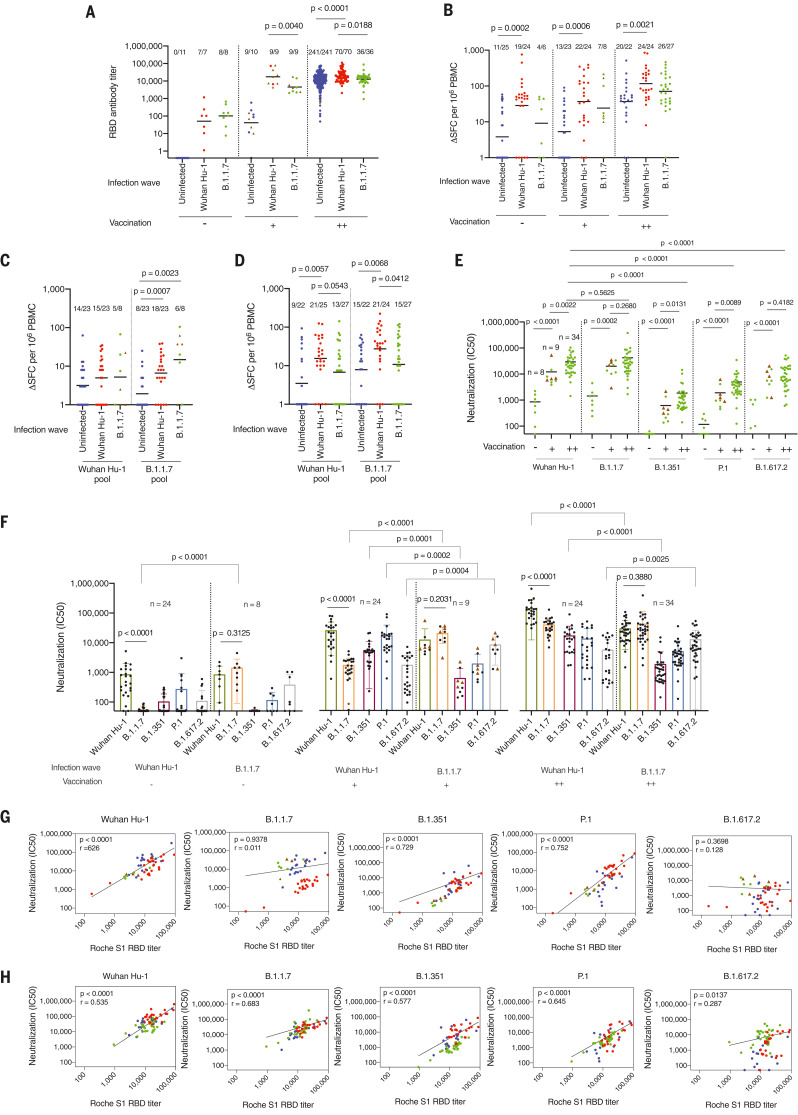
T cell and B cell immunity and neutralization hierarchy after heterologous exposure through infection with the B.1.1.7 VOC during the second UK wave and in the context of single- and two-dose vaccination. (**A**) RBD Ab titers at 54 to 57 weeks after the start of study recruitment in March 2020 in HCWs who were not infected with SARS-CoV-2 (blue, *n* = 256), those infected during the first UK wave by the Wuhan Hu-1 strain (red, *n* = 86), and those infected during the second UK wave by the B.1.1.7 (green, *n* = 53). (**B**) Magnitude of T cell response to Wuhan Hu-1 spike MEP peptide pool. Data are plotted according to whether individuals were unvaccinated (–) or had received one (+) or two (++) doses of BNT162b2 vaccine. (**C** and **D**) Magnitude of T cell response to B.1.1.7 peptide pools (Wuhan Hu-1 or B.1.1.7 variant peptides) after first dose (C) or second dose (D) of vaccine. (**E** and **F**) Neutralizing Ab titers (IC_50_) against authentic Wuhan Hu-1 live virus and B.1.1.7, B.1.351, P.1, or B.1.617.2 VOCs in HCWs infected with SARS-CoV-2 during the B.1.1.7 wave (E) and during the Wuhan Hu-1 first UK wave (F), plotted according to whether individuals were unvaccinated (–, Wuhan Hu-1 wave, *n* = 24; B.1.1.7 wave, *n* = 8) or had received one dose (+, Wuhan Hu-1 wave *n* = 24, B.1.1.7 wave *n* = 9) or two doses (++, Wuhan Hu-1 wave *n* = 24, B.1.1.7 wave *n* = 34) of vaccine. (**G** and **H**) Correlation between Roche S1 RBD Ab titer and nAb (IC_50_) against authentic Wuhan Hu-1 live virus and B.1.1.7, B.1.351, P.1, and B.1.617.2 VOC in one-dose (G) and two-dose (H) BNT162b2 vaccinated HCWs previously infected by Wuhan Hu-1 (red, *n* = 23) or B.1.1.7 infected (green, one dose *n* = 9, two doses *n* = 31) and two-dose vaccinated infection-naïve (blue, *n* = 19) HCWs at 54 to 57 weeks after initial study recruitment. In all graphs, individuals who received the ChAdOx1 nCoV-19 vaccine are marked as brown triangles. In (A) to (E), Mann-Whitney *U* test was used; in (F), Mann-Whitney *U* test or Wilcoxon matched-pairs signed rank test was used; and in (G) and (H), Spearman’s rank correlation was used.

We then looked at the impact of infection with B.1.1.7 during the second wave on immune responses in individuals given vaccine expressing Wuhan Hu-1 spike. For this part of the study, an additional 358 HCWs were followed up at 55 to 57 weeks after recruitment into the study (fig. S1). Of these, 63 had been infected with SARS-CoV-2 during the first Wuhan Hu-1 wave. Fifty-three of previously uninfected HCWs (53/296, 18%) were identified by virtue of longitudinal N serology (from baseline to 16 to 18, 28 to 30, 42, 54, and 55 to 57 weeks) as having been newly infected during the B.1.1.7 s wave. Thirty-six (68%) were double-vaccinated and eight (15%) unvaccinated at the time of follow-up (fig. S1 and tables S5 and S6). Five previously infected HCWs were reinfected (5/63, 8%) during the second wave. S1 IgG titers were measured at 55 to 57 weeks (fig. S6B). The median date of a positive SARS-CoV-2 PCR test was 28 December 2020 (IQR = 22 d). The B.1.1.7 VOC accounted for 94.7% of SARS-CoV-2 infections in central London in the 2 weeks leading up to 2 January 2021 (*28*); we thus made the starting assumption that for most HCW-infected individuals during the second wave, the infecting strain was B.1.1.7. To support this, we compared the Wuhan Hu-1 and B.1.1.7 nAb IC_50_ results for unvaccinated (*n* = 24 and 8), single-dose vaccinated (*n* = 25 and 9), and two-dose vaccinated (*n* = 24 and 34) HCWs infected during the first and second UK waves by Wuhan Hu-1 and B.1.1.7, respectively (fig. S7). In unvaccinated HCWs, those infected during the first wave (fig. S7A, LHS) had nAb IC_50_ responses against Wuhan Hu-1, but negligible or no nAb IC_50_ response against B.1.1.7 (*P* < 0.0001). By contrast, HCWs infected as B.1.1.7 peaked (fig. S7A, RHS) had similar nAb IC_50_s against B.1.1.7 and Wuhan Hu-1 live virus. In Wuhan Hu-1–infected HCWs, the Wuhan Hu-1 and B.1.1.7 nAb IC_50_ increased and remained significantly different after one-dose vaccination (*P* < 0.0001) and two-dose vaccination (*P* < 0.0001), but the differential between them decreased (Fig. 3F and fig. S7, B and C).

We set out to investigate the impact of heterologous antigen exposure with the B.1.1.7 variant. A confounder in assessing comparative postvaccination immunity between HCWs infected during the first wave (Wuhan-Hu-1) and those infected during the second wave (B.1.1.7) is that they differ with respect to the infecting strain and in the time interval between infection and vaccination. We hypothesized that observed differences in immunity may relate to recall differences between heterologous spike sequences [immune imprinting (*16*–*18*)] and/or to differences in affinity maturation of the Ab repertoire with time from initial infection. Somatic hypermutation and breadth of neutralizing response is known to improve with time from infection (*29*–*31*), but there may also be intrinsic differences between the breadth and hierarchy of neutralizing responses primed by different variants (*32*). We initially compared nAb profiles for neutralization of Wuhan Hu-1 and VOC Alpha to Delta at a similar number of weeks after PCR-confirmed SARS-CoV-2 infection with Wuhan Hu-1 during the first wave and B.1.1.7 during the second wave (all unvaccinated HCWs). These data support the contention that there are qualitatively and quantitatively differential patterns, even matching for time point (fig. S9).

After a single vaccine dose, infection-naïve HCWs had an S1 RBD response similar to that seen after infection with B.1.1.7. Those HCWs who were infected by Wuhan Hu-1 12 months earlier had a 3.9-fold higher S1-RBD response than those infected more recently by the B.1.1.7 variant (*P* = 0.004; Fig. 3A). After two vaccine doses, HCWs infected during March 2020 by Wuhan Hu-1 (*n* = 70), having had three homologous antigen exposures, had a 1.5-fold higher S1-RBD response than those encountering three antigen exposures, with one of these being heterologous B.1.1.7 (*n* = 36, *P* = 0.0188; Fig. 3A). The B.1.1.7–infected two-dose vaccinated HCWs had an S1-RBD response similar to that of the two-dose infection-naïve vaccinees. Infection-naïve vacccinees had a 1.8-fold lower response compared with the Wuhan Hu-1 previously infected group (*n* = 241, *P* < 0.0001; Fig. 3A). The implication of this is that the phenomenon of enhanced vaccine responses by infection, which has been reproducibly described by us and others (*8*, *9*, *11*–*14*), is less effective if the infection involves heterologous spike from a VOC.

After two vaccine doses, individuals infected during the Wuhan Hu-1 wave had a higher T cell response to the B.1.1.7 peptide pool than those infected during the B.1.1.7 wave (*P* = 0.0412), arguing for stronger T cell priming in those with three homologous exposures (Fig. 3, B to D).

We next considered the effect of B.1.1.7 infection in single and double vaccination. In two-dose vaccinated individuals, B.1.1.7 infection resulted in a different hierarchy of nAb IC_50_ responses against VOCs compared with after Wuhan Hu-1 infection: Wuhan Hu-1 > B.1.1.7 > B.1.351 > P.1 > B.1.617.2. In the context of heterologous B.1.1.7 infection and two-dose vaccination, the hierarchy changed to B.1.1.7 > Wuhan Hu-1 > B.1.617.2 > P.1 > B.1.351 (Fig. 3, E and F). This indicates a process of selective discriminative heterologous imprinting after exposure to B.1.1.7 infection (Fig. 3, E and F, and fig. S8). As predicted for immune imprinting by SARS-CoV-2 variants, the first encounter imparts a differential subsequent pattern (*16*–*18*).

To better understand the relationship between differential nAb IC_50_ and S1 RBD binding, we correlated the two. First we compared the impact of two homologous antigen exposures [either two-dose vaccination (blue, infection-naïve) or prior Wuhan Hu-1 infection followed by single-dose vaccination (red, Wuhan Hu-1)] with heterologous exposure through B.1.1.7 infection [single-dose vaccination and B.1.1.7 infection (green, B.1.1.7)]. Two-dose vaccinated infection-naïve HCWs (blue) achieved higher neutralization against authentic Wuhan Hu-1 live virus than those with heterologous infection by B.1.1.7 and single-dose vaccination (green) (*P* = 0.0036; Fig. 3G and fig. S10A). Individuals infected by B.1.1.7 and single-dose vaccinated (green) had higher nAb IC_50_ against B.1.1.7 compared with two-dose vaccinated infection-naïve HCWs (*P* < 0.0001) (blue) and Wuhan Hu-1 previously infected single-dose vaccinated HCWs (*P* < 0.0001) (red) (Fig. 3G and fig. S10A). Furthermore, neutralization IC_50_ of B.1.351 live virus was less potent after heterologous B.1.1.7 infection and single-dose vaccination (green) (*P* = 0.0001; Fig. 3G and fig. S10A). After three antigen encounters, the impact of heterologous exposure became more pronounced. HCWs with heterologous infection with B.1.1.7 (green) rather than Wuhan Hu-1 (red) had substantially lower neutralization against authentic Wuhan Hu-1 (*P* < 0.0001), B.1.351 (*P* < 0.0001), and P.1 (*P* = 0.0616) live virus (fig. S10B). Furthermore, two-dose vaccinated HCWs with a history of heterologous infection by B.1.1.7 had lower nAb IC_50_s against B.1.351 than infection-naïve double vaccinated HCWs (*P* = 0.0007; fig. S10B). By contrast, two-dose vaccinated HCWs with a history of heterologous infection with B.1.1.7 (green) had higher nAb IC_50_ against B.1.617.2 than infection-naïve two-dose vaccinated HCWs (*P* < 0.0001; fig. S10B). Thus, the neutralization potency achieved was dependent on the number of antigen encounters and the presence or absence of heterologous exposure. Ab neutralization is a widely accepted correlate of protection (COP), estimated at an nAb IC_50_ of >15, and S1 RBD binding is an accessible and accepted marker of this (*22*). Our data here show that S1 RBD and VOC S1 RBD titers are not a reliable COP for VOC, especially in the context of selective discriminative heterologous exposure during infection with B.1.1.7. In HCWs with heterologous B.1.1.7 exposure, the nAb IC_50_ of B1.351 was significantly reduced (*P* < 0.0001) and B.1.617.2 was significantly increased (*P* = 0.0025) compared with HCWs with homologous Wuhan Hu-1 infection.

## Differential longitudinal immunity after heterologous infection

**Fig. 4.**
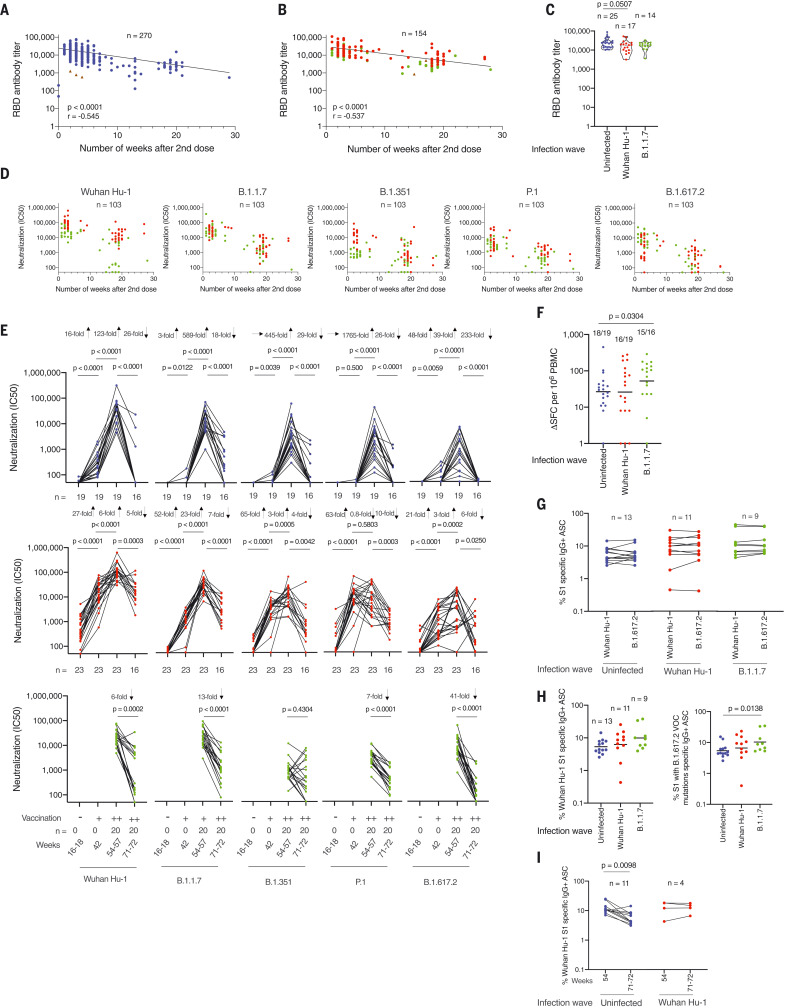
Differential impact of heterologous exposure through B.1.1.7 infection on neutralization of other VOC and durability of the immune response. (**A**) S1 RBD Ab titers in HCWs with no previous SARS-CoV-2 infection (blue) and two-dose vaccination (*n* = 270). (**B**) S1 RBD Ab titers in HCWs with a history of SARS-CoV-2 infection (Wuhan Hu-1, red; B.1.17, green) and two-dose vaccination (*n* = 154), plotted by the number of weeks that serum was sampled after the second vaccine dose. HCWs who received the ChAdOx1 nCoV-19 vaccine are indicated by brown triangles. (**C**) S1 RBD Ab titers in infection-naïve HCWs (blue, *n* = 25) or those with a history of SARS-CoV-2 infection with the Wuhan Hu-1 strain (red, *n* = 17) or B.1.17 VOCs (green, *n* = 14) and three-dose vaccination sampled between 10 d and 7 weeks after the third vaccine dose. (**D**) Neutralizing Ab titers (IC_50_) against authentic Wuhan Hu-1 live virus and B.1.1.7, B.1.351, P.1, and B.1.617.2 VOC in HCWs with a history of previous SARS-CoV-2 infection who had received two vaccine doses, plotted by the number of weeks that serum was sampled after the second vaccine dose. (**E**) Neutralizing Ab titer (IC_50_) against authentic Wuhan Hu-1 live virus and B.1.1.7, B.1.351, P.1, and B.1.617.2 VOCs plotted longitudinally 20 d after the first (+) and 20 days after the second (++) dose and then 21 weeks after the second BNT162b2 vaccination. Shown are SARS-CoV-2 infection-naïve HCWs (blue, upper panel), Wuhan Hu-1 previously infected HCWs (red, middle panel), and B.1.1.7–infected HCWs (green, lower panel). (**F**) Magnitude of T cell response to spike MEP peptide pool at 71 to 72 weeks after initial study recruitment in double-vaccinated infection-naïve HCWs (blue, *n* = 19) and HCWs with a history of SARS-CoV-2 infection by Wuhan Hu-1 (red, *n* = 19) or B.1.1.7 (green, *n* = 16). (**G**) Percentage of IgG^+^ ASCs specific for Wuhan Hu-1 S1 or S1 protein containing T19R, G142D, del 156-157, R158G, L452R, T478K, D614G, and P681R (B.1.617.2 VOC) mutations at 71 to 72 weeks after initial study recruitment in double-vaccinated infection-naïve (blue, *n* = 13) HCWs and those with a history of SARS-CoV-2 infection with the Wuhan Hu-1 strain (red, *n* = 11) and B.1.1.7 VOC (green, *n* = 9) 21 weeks after the second vaccine dose. (**H**) Percentage of IgG^+^ ASCs specific for Wuhan Hu-1 S1 protein (left panel) or S1 protein containing T19R, G142D, del 156-157, R158G, L452R, T478K, D614G, and P681R (B.1.617.2 VOC) mutations (right panel) at 71 to 72 weeks after initial study recruitment in double-vaccinated infection-naïve HCWs (blue, *n* = 13), previously Wuhan Hu-1–infected HCWs (red, *n* = 11), and B.1.1.7–infected HCWs (green, *n* = 9). (**I**) Percentage of IgG^+^ ASCs specific for Wuhan Hu-1 S1 protein in double-vaccinated HCWs with (red, *n* = 4) or without (blue, *n* = 11) a history of Wuhan Hu-1 infection at 54 and 71 to 72 weeks after initial study recruitment. In (A) and (B), Spearman’s rank correlation was used; in (C), (F), and (H), Mann-Whitney *U* test was used; and in (E) and (I), Wilcoxon matched-pairs signed rank test was used.

Next, we considered the relative durability of immunity after three-dose homologous (Wuhan Hu-1 infection plus vaccination) and heterologous (B.1.1.7 infection plus vaccination) antigen compared with two-dose vaccinated infection-naïve individuals. S1 RBD Ab levels declined as the number of weeks after the second dose increased in both infection-naïve (*r* = 0.545; *P* < 0.0001) and previously infected (*r* = 0.537; *P* < 0.0001) HCWs (Fig. 4, A and B, and tables S1, S5 to S7, and S9). After a third vaccine dose, S1 RBD binding increased in all three groups to a similar level (Fig. 4C). B.1.1.7 infection (green circles) compared with Wuhan Hu-1 infection (red circles) was associated with lower nAb IC_50_ against Wuhan Hu-1 (*P* < 0.0001) and B.1.351 (*P* < 0.0001) VOC initially at (10 d to 8 weeks after vaccination) and P.1 (*P* = 0.0020) 8 weeks later (12 to 22 weeks after vaccination), indicating that differential nAb responses over time depend on the VOC (Fig. 4D). Two-dose vaccinated infection-naïve HCWs were at a lower starting value for nAb IC_50_ against B.1.617.2 compared with HCWs infected with B.1.1.7 (*P* < 0.0001) and Wuhan Hu-1 (*P* = 0.0462) (Fig. 3H and fig. S10B). The nAb IC_50_ against authentic Wuhan Hu-1 and all four VOCs (B.1.1.7, B.1.351, P.1, and B.1.617.2) fell differentially depending on the VOC being neutralized and SARS-CoV-2 infection history (Fig. 4E).

In two-dose vaccinated infection-naïve HCW, nAb IC_50_ against authentic Wuhan Hu-1 and all four of the VOCs fell significantly over 18 weeks (Wuhan Hu-1, 26-fold, *P* < 0.0001; B.1.1.7, 18-fold, *P* < 0.0001; B.1.351, 29-fold, *P* < 0.0001; P.1. 26-fold, *P* < 0.0001; B.1.617.2 233-fold, *P* < 0.0001). This was most marked for B.1.617.2 live virus neutralization; by 21 weeks after the second dose, it was zero for 21/27 (78%) HCWs tested (median = 0, IQR = 0, *n* = 27) (Fig. 4E). For two-dose vaccinated HCWs previously infected with Wuhan Hu-1, there was a significantly and less pronounced fall in nAb IC_50_ against authentic Wuhan Hu-1 and three of the VOCs over 18 weeks (Wuhan Hu-1, fivefold, *P* = 0.0003; B.1.1.7, sevenfold, *P* < 0.0001; B.1.351, fourfold, *P* = 0.0042; P.1. 10-fold, *P* = 0.0003). By contrast, in the presence of heterologous infection with B.1.1.7 and two-dose vaccination, nAb IC_50_ fell significantly over 18 weeks for some variants (Wuhan Hu-1, sixfold, *P* = 0.0002; B.1.1.7 13-fold, *P* < 0.0001; P.1 sevenfold, *P* < 0.0001; and B.1.617.2 41-fold, *P* < 0.0001), whereas nAb IC_50_ against B.1.351 did not (*P* = 0.4304).

T cell responses against spike MEP pool 21 weeks after the second dose were higher in two-dose vaccinated HCWs that were infected with B.1.1.7 than in two-dose vaccinated infection-naïve HCWs (*P* = 0.0304; Fig. 4F).

MBC frequencies against the Wuhan Hu-1 S1 and S1 containing the B.1.617.2 mutations were equivalent 21 weeks after second dose vaccination (Fig. 4G). Two-dose vaccinated B.1.1.7 infected HCW had higher frequency responses against S1 containing the B.1.617.2 mutations than two-dose vaccinated infection-naïve HCWs (*P* = 0.0138; Fig. 4H). MBC frequencies in two-dose vaccinated infection-naïve HCWs fell over 18 weeks (*n* = 11, *P* = 0.0098), whereas those from Wuhan Hu-1 previously infected HCWs were sustained (*n* = 4; Fig. 4I).

## Immune parameters associated with B.1.617.2 breakthrough infection

At the time of the 71- to 72- and 83- to 84-week recruitments, B.1.617.2 VOC infections accounted for 97.8 and 99.9% of UK SARS-CoV-2 infections, respectively (*26*)*.* We identified 6/80 (8%) and 14/74 (19%) B.1.617.2 breakthrough infections in the double-vaccinated HCWs. Ten were previously infection-naïve, four had previous Wuhan Hu-1 infection, and six were infected with B.1.1.7 (tables S8 and S10). PCR-positive confirmed B.1.617.2 breakthrough infections occurred in the context of S1 RBD Ab levels ranging 1110 to 29,308 U/ml (median = 9010, IQR = 13,650) 2 to 3 weeks after the second vaccine dose. We evaluated the S1 RBD, nAb, T cell, and MBC data in these double-vaccinated HCW before (purple circle) and after (lilac circle) their B.1.617.2 breakthrough infection (fig. S11, A to J). Analysis showed that both previously infected and infection-naïve HCWs with S1 RBD Ab responses (20 d after the second dose) of >1100 U/ml had become infected, and breakthrough infection was not specifically associated with being a low responder (fig. S11, A and B). Relatively potent nAb IC_50_ at 20 d after the second vaccine dose had fallen 12 weeks later. This fall was more pronounced in the infection-naïve double-vaccinated HCWs (fig. S11, D and E). For B.1.617.2, the nAb IC_50_ fell to zero in two-dose vaccinated infection-naïve HCWs (fig. S11, D and G). B.1.617.2 breakthrough infections have been linked to low nAb responses (*33*). Current estimates for COP apply poorly in the case of B.1.617.2 (*22*). In the cases reported here, breakthrough infections occurred in the face of good prior S1 RBD Ab titers, nAb IC_50_s, and MBC frequency 20 d after second vaccine dose, but responses fell in the subsequent 18 weeks, especially in the infection-naïve two-dose vaccinated HCWs. Initial immune responses poorly predicted protection against B.1.617.2 breakthrough infection (fig. S11, A to J).

## Discussion

The new challenge in a world facing diverse VOCs is to understand both cross-neutralization of these by first-generation spike vaccines and also how protective immunity is differentially shaped in those who have had infection by the different VOCs (*34*). These real-life issues of immune imprinting have implications for the optimal design of second-generation variant spike vaccine boosters in parts of the world experiencing different VOCs or for assessing heterogeneity in the quantity, quality, and durability of immune protection in those who have experienced different combinations of infecting and vaccinating sequences. Infection by B.1.1.7 has different features from Wuhan Hu-1 in terms of protection against VOCs, which is evident in the response to infection and in the differential impact of vaccine-boosting previously described (*8*, *9*, *11*–*14*). Differential skewing of the immune repertoire may occur if the prime and/or boost is through B.1.1.7, because epitope modification at DY144 and N501Y changes the nAb repertoire (*4*, *35*). We found a differential hierarchy of cross-neutralization to VOCs after natural infection or one or two vaccine doses, depending on the infecting strain. That different immune priming by B.1.1.7 is likely a consequence of heterologous virus per se and not of a shorter time span for affinity maturation is supported by data from Israel, confirming that susceptibility grows with time and with declining Ab levels (*36*). Vaccine programs currently use a prototypic Wuhan Hu-1 sequence against a background of infection by different VOCs predominating in different parts of the world. Decoding the differential breadth of VOC-neutralizing responses ensuing from diverse priming combinations will affect which variant spike sequences may best serve in second-generation vaccines. This appears to be a more complex choice than simply opting for the most concerning variant at any given time. Because heterologous combinations can confer a diminished response against other variants, there may be a case for sticking with the Wuhan Hu-1 sequence in booster vaccinations in the first instance in the face of unknown future VOCs or for improved efforts to define vaccines based on optimization of common, conserved neutralizing epitopes (*37*). In any case, the inference from this cohort is that populations infected during waves of different variants carry distinct immune memory, with implications for differential protection against future VOCs.

## Methods summary

Detailed materials and methods are provided in the supplementary materials. Recruitment of 731 HCWs into the COVIDsortium cohort followed longitudinally with weekly self-reported symptom diaries, SARS-CoV-2 PCR, N, and S1 RBD serology for 16 weeks from the start of the first UK wave has been described previously (*5*–*10*). This enabled a cross-sectional, case-controlled sub-study of 136 HCWs recruited 16 to 18 weeks after the March 2020 UK lockdown that reported discordant neutralizing antibody and T cell responses in SARS-CoV-2 natural infection during the first wave (Wuhan Hu-1) (*6*). A cross-sectional, case-controlled vaccine sub-study cohort of 51 HCWs at 22 d after first BNT162b2 dose described vaccine immunity in HCWs with (*n* = 25) and without (*n* = 26) prior SARS-CoV-2 infection during the Wuhan Hu-1 wave (*8*, *9*). The current sub-study includes longitudinal follow-up of this previously published vaccine sub-study cohort (*n* = 51) at 20 d (IQR = 7) after a second BNT162b2 dose plus an additional 358 HCWs, 53 of whom were infected with B.1.1.7 during the second UK wave. At 71 to 72 weeks, 80 two-dose vaccinated HCWs were re-recruited 18 to 21 weeks after their second dose for longitudinal follow-up of SARS-CoV-2 infection-naïve (*n* = 27) or HCWs previously infected during the Wuhan Hu-1 UK wave (*n* = 31) or the second B.1.1.7 UK wave (*n* = 22). At 83 to 84 weeks, 74 previously two-dose vaccinated HCWs who were infection naïve (*n* = 30) or infected during the Wuhan Hu-1 wave (*n* = 18) or the B.1.1.7 wave (*n* = 19) were re-recruited 30 to 33 weeks after their second dose. Sixty-seven (91%) were within a median of 18 d (IQR = 12) after their third BNT162b2 dose. HCWs with breakthrough infection by B.1.617.2 were identified by PCR and N serology. Peripheral blood mononuclear cells and serum samples were prepared and cryopreserved as previously described (*6*, *9*). Anti-nucleocapsid [cutoff index (COI) ≥ 1.0 U/ml, positive] and anti-spike antibody (COI ≥ 0.8 U/ml, positive) detection Ab testing was conducted at UK Health Security Agency (UKHSA) using the Roche Cobas e801 analyzer. Recombinant proteins were used in ELISAs looking at the VOC S1 RBD responses: SARS-CoV-2 spike glycoprotein (S1) RBD, SARS-CoV-2 (N501Y mutant), (501Y.V2: K417N, E484K, N501Y), (B.1.1.28: K417T, E484K, N501Y), and (B.1.617.2: L452R, T478K) spike glycoprotein (S1) RBDs derived from Wuhan Hu-1, B.1.1.7, B.1.351, P.1, and B.1.617.2 VOC, respectively. T cell experiments peptide panels included an MEP composed of a pool of 18 12- to 20-mer peptide epitopes (*6*, *9*), and VOC pools contained peptides from the B.1.1.7, B.1.351, P.1, and B.1.617.2 sequences and their respective Wuhan Hu-1 sequence (table S2). IFNγ-T cell ELISpots and MBC ELISpots were performed as previously described (*6*, *9*). MBC ELISpot plates were coated with phosphate-buffered saline, purified anti-human IgG MT91/145, SARS-CoV-2 S1 spike, E484K, K417N, N501Y spike or T19R, G142D, del 156-157, R158G, L452R, T478K, D614G, P681R spike. In silico predictions of HLA-DRB1/peptide binding were performed using NetMHCIIpan-4.0 (*9*, *38*, *39*). Studies using HLAII transgenics (DRB1*0401) were performed as previously described (*9*, *40*, *41*). For transcriptomic and flow cytometry analysis, mouse lymph node cells were cultured with no peptide and wild-type or variant N501Y peptides, and at 24 hours, cells were harvested and lysed for RNA extraction or stained for flow cytometry. SARS-CoV-2 microneutralization assays were performed as described previously using VeroE6 cells (*6*, *9*). Participant sera were incubated with 3 × 10^4^ focus-forming units of SARS-CoV-2 (Wuhan Hu-1, B.1.1.7, B.1.351, P.1, or B.1617.2 isolates of SARS-CoV-2 live virus).

## Supplementary Material

20211202-1

## Supplementary Materials

### This PDF file includes:

Materials and Methods Figs. S1 to S11 Tables S1 to S13 Members of the COVIDsortium Immune Correlates Network and COVIDsortium Investigators References (*42*, *43*)

### Other Supplementary Material for this manuscript includes the following:

Data Table S14

MDAR Reproducibility Checklist

## Notes

View/request a protocol for this paper from *Bio-protocol*.
