# Supplementary material for: Heterologous infection and vaccination shapes immunity against SARS-CoV-2 variants
Source: Science. 2021 Dec 2;375(6577):183–92. doi: 10.1126/science.abm0811 (PMC10186585; doi:10.1126/science.abm0811)
Supplement: 20211202-1 [file science.abm0811.v1.pdf]

Cite as: C. J. Reynolds *et al.*, *Science* 10.1126/science.abm0811

# Heterologous infection and vaccination shapes immunity against SARS-CoV-2 variants

**Catherine J. Reynolds[1]†, Joseph M. Gibbons[2]†, Corinna Pade[2]†, Kai-Min Lin[1], Diana Munoz Sandoval[1], Franziska Pieper[1], David K. Butler[1], Siyi Liu[1], Ashley D. Otter[3], George Joy[4], Katia Menacho[4], Marianna Fontana[5], Angelique Smit[5], Beatrix Kele[4], Teresa Cutino-Moguel[4], Mala K. Maini[6], Mahdad Noursadeghi[6], COVIDsortium Immune Correlates Network‡, Tim Brooks[3], Amanda Semper[3], Charlotte Manisty[4,7], Thomas A. Treibel[4,7], James C. Moon[4,7], COVIDsortium Investigators‡, Áine McKnight[2]§, Daniel M. Altmann[8]§, Rosemary J. Boyton[1,9]§***

[1]Department of Infectious Disease, Imperial College London, London, UK. [2]Blizard Institute, Barts and the London School of Medicine and Dentistry, Queen Mary University of London, London, UK. [3]UK Health Security Agency, Porton Down, UK. [4]St. Bartholomew's Hospital, Barts Health NHS Trust, London, UK. [5]Royal Free London NHS Foundation Trust, London, UK. [6]Division of Infection and Immunity, University College London, London, UK. [7]Institute of Cardiovascular Science, University College London, London, UK. [8]Department of Immunology and Inflammation, Imperial College London, London, UK. [9]Lung Division, Royal Brompton and Harefield Hospitals, Guy's and St. Thomas' NHS Foundation Trust, London, UK.

†These authors contributed equally to this work and are co-first authors.

‡The members of the COVIDsortium Immune Correlates Network and the COVIDsortium Investigators are listed in the supplementary materials.

§These authors contributed equally to this work and are co-senior authors.

*Corresponding author. Email: r.boyton@imperial.ac.uk

**The impact of initial severe acute respiratory syndrome coronavirus 2 (SARS-CoV-2) infecting strain on downstream immunity to heterologous variants of concern (VOC) is unknown. Studying a longitudinal healthcare worker cohort, we found that after three antigen exposures (infection+two vaccine doses), S1 antibody, memory B cells and heterologous neutralization of B.1.351, P.1 and B.1.617.2 plateaued, while B.1.1.7 neutralization and spike T cell responses increased. Serology using Wuhan Hu-1 spike receptor binding domain poorly predicted neutralizing immunity against VOCs. Neutralization potency against VOCs changed with heterologous virus encounter and number of antigen exposures. Neutralization potency fell differentially depending on targeted VOCs over 5-months from the second vaccine dose. Heterologous combinations of spike encountered during infection and vaccination shape subsequent cross-protection against VOC, with implications for future-proof next-generation vaccines.**

After experiencing >18 months of the COVID-19 pandemic, immunological analysis has shifted to issues of response durability, boosting, and mitigation against future variants of concern (VOC). In doing so we confront viral and population heterogeneities that affect immunity and future protection (*1*–*4*). Individuals are either immunologically naïve to severe acute respiratory syndrome coronavirus 2 (SARS-CoV-2) or have been infected with either the original Wuhan Hu-1 strain or one of the alpha to delta variants of concern (VOC; which we will refer to by full Pango lineage terms). Infection-naïve individuals, those infected by ancestral SARS-CoV-2 Wuhan Hu-1 or a VOC may have received different numbers of vaccine doses. Thus, there is a spectrum of individuals spanning the immunologically naïve to those who have experienced one, two, three exposures (and four with boosting) to homologous or heterologous spike sequences. The challenge is to understand whether different antigen exposure combinations are associated with the same quality, quantity and durability of immunity, and ability to cross-protect against other VOCs.

Using a cohort of UK healthcare workers (HCW) for which we have extensive, longitudinal, clinical, transcriptomic, and immunologic characterization (*5*–*10*), we address these questions by immunological comparison of BNT162b2 (Pfizer-BioNTech vaccine) vaccinees, with or without infection. We and others have shown the boosting effect of prior infection by the Wuhan Hu-1 strain on the response to vaccination with homologous spike (*8*, *9*, *11*–*14*), and others are decoding changes in immune programming between first and second dose (*15*). We compared the impact of prior infection with Wuhan Hu-1 or B.1.1.7. (alpha VOC) in the context of vaccination on T and memory B cell (MBC) responses, cross-neutralization against VOC, durability of immunity and susceptibility to B.1.617.2 (delta VOC) breakthrough infection. We considered the extent to which first encounter with spike sequence shapes subsequent response features to explore the possible effect of immune imprinting or 'original antigenic sin' (*16*–*18*).

### B and T cell immunity after three homologous antigen exposures

We analyzed this longitudinal vaccine cohort ($n$ = 51) at 20d (interquartile range, IQR 7) after receiving a second BNT162b2 dose (fig. S1 and table S1). Twenty-five HCW were infected with SARS-CoV-2 approximately synchronous and coincident with peak transmission in London in March 2020 (*6*). At one year, nucleoprotein (N) antibody (Ab) responses had waned but were still positive (anti-N Ab expressed as a cut-off index of ≥1.0 were classified as positive), 16% having fallen to sub-threshold levels (Fig. 1A). Two (2/25, 8%) HCW appeared to have been reinfected (increase in N) and two (2/26, 8%) newly infected (positive N having been previously negative) among the previously uninfected cohort (Fig. 1A). T cell responses to N were sustained at 1 year in the majority of prior infected individuals (22/24, 92%) (fig. S2). All prior infected HCW with positive anti-S1 receptor binding domain (RBD) responses at 16-18 weeks had sustained responses at 28-30 weeks (22/24, 92%), which substantially enhanced following single dose vaccination (36-fold increase in Ab titer) (Fig. 1B). This response plateaued (only increased a further 1.4-fold) following second dose vaccination (third antigen exposure). The prior infected HCW (2/24, 8%) with unrecordable anti-S1 RBD responses at 16-18 and 28-30 weeks showed a 155-fold increase anti-S1 response following second dose vaccination, demonstrating the benefit of a third antigen exposure in poor Ab responders (*19*, *20*). Infection-naïve HCW made incrementally increased Ab responses following first and second dose vaccination, achieving about half the Ab titer of their previously infected counterparts 20 days after second vaccine dose (Fig. 1B).

T cell responses to spike mapped epitope peptide (MEP) pool (table S2) increased following second dose vaccination in prior infected individuals (i.e., third exposure to antigen) ($P$ = 0.0294; Fig. 1C). T cell responses following two dose vaccination in infection-naïve HCW achieved similar levels to those seen at 16-18 weeks after natural SARS-CoV-2 infection. However, there were fewer non-responders (2/23, 9%) in double vaccinated infection-naïve individuals compared with (5/25, 20%) 16-18 weeks after natural infection.

Infection-naïve individuals showed incremental increases in neutralizing Ab (nAb) to authentic Wuhan Hu-1 virus and VOCs following first and second vaccination, the largest fold increase occurring after the second vaccine dose (Wuhan Hu-1,123-fold; B.1.1.7, 589-fold; B.1.351, 445-fold; P.1, 1765-fold; and B.1.617.2, 39-fold increase). There was a wider heterogeneity of nAb response to B.1.351, P.1 and B.1.617.2 VOCs compared to B.1.1.7 and Wuhan Hu-1 (Fig. 1D). Individuals who had experienced SARS-CoV-2 infection prior to vaccination also showed the largest fold nAb increase on second antigen exposure (i.e., after first vaccine dose) (Wuhan Hu-1, 27-fold; B.1.1.7, 52-fold; B.1.351, 65-fold; P.1, 63-fold; and B.1.617.2, 20-fold increase) (Fig. 1E). In contrast, nAb responses against Wuhan Hu-1 and VOCs B.1.351, P.1 and B.1.617.2 plateaued or decreased between the second and third antigen exposure. This was not the case for nAb responses against Wuhan Hu-1 and B.1.1.7, which increased 6-fold and 23-fold respectively between the second and third antigen exposure (Fig. 1E). The potency of nAb response after third antigen exposure varied depending on the VOC being neutralized. Prior infected HCW who made a low nAb IC50 after the first vaccine dose caught up following the second vaccine dose (i.e., third antigen exposure). Heterologous neutralizing IC50s were lower than those against homologous Wuhan Hu-1 and at risk of falling below a threshold for protection as levels wane (*21*, *22*) (1F, S3).

There was a positive correlation between S1 RBD binding Ab and neutralization (IC50), with previously infected individuals showing higher nAb IC50 and S1 RBD binding (Fig. 1G). Wuhan-Hu-1 sequence specific S1 RBD binding correlated less well with nAb IC50 against VOCs B.1.351, P.1 and B.1.617.2, especially for infection-naïve, double vaccinated HCW. To establish if this was due to different sequences in the VOC RBD, the relationship between VOC RBD binding titers and neutralization was explored. There was positive correlation between the Roche S1 RBD and VOC RBD specific binding (fig. S4). The weaker correlations between VOC nAb titers and VOC RBD binding indicate that antibodies targeting regions outside of RBD may contribute to neutralization and therefore the binding titer is not predictive of neutralization (Fig. 1H). For example, a two-dose vaccinated HCW with an S1 RBD (Wuhan Hu-1) binding titer of 2950 U/ml, considered strongly positive, showed divergent neutralization of VOCs, with IC50s of: Wuhan Hu-1, 6,071, B.1.1.7, 1,037; B.1.351, 301; P.1, 560 and B.1.617.2 zero 20d after second dose vaccination that all fell to undetectable 18 weeks later. This individual was later infected by B.1.617.2. Thus, S1 serology data using the Wuhan Hu-1 S1 RBD and VOC sequence is an unreliable marker for neutralization potency against VOCs.

For infection-naïve subjects, frequency of S1 specific MBC 20d after first-dose vaccination was lower compared to 16-18 weeks after infection. On the other hand, MBCs were amplified in previously infected HCWs to a level similar to that seen following two doses of vaccine (Fig. 1I). As expected from the S1 RBD binding Ab and nAb responses, MBC responses plateaued, with no further enhancement following a third antigen exposure (i.e., double vaccinated previously infected HCW) (Fig. 1, I and J). For infection-naïve HCW, the MBC frequency for specificity to S1 containing the N501Y, E484K and K417N B.1.351 mutations was lower after one vaccine dose (Fig. 1K, left panel). After two (two dose vaccination or single dose vaccination and prior infection) or three antigen exposures (two dose vaccination and prior infection), the MBC response is maintained whether stimulated by S1 or S1

containing mutations found in B.1.351 and B.1.617.2 (Fig. 1K, middle and right panels). There was a high frequency of MBC able to recognize S1 and S1 containing VOC specific mutations, but not always correlated well with a VOC specific nAb response (fig. S5). Authentic P.1 ($r$ = 0.529, $P$ = 0.0138) and B.1.617.2 ($r$ = 0.548, $P$ = 0.001) nAb responses of double vaccinated HCW with and without prior Wuhan Hu-1 infection positively correlated with the MBC frequency against S1 containing the B.1.617.2 specific mutations (fig. S5C).

Next, we examined the T cell response against VOCs B.1.1.7, B.1.351, P.1, B.1.617.2 peptide pools and Wuhan Hu-1 matched pools and individual peptides with substituted epitopes covering N501Y and D1118H (table S2 and Fig. 2, A and B). T cell responses in doubly vaccinated, infection-naïve individuals were variable. Previously infected doubly vaccinated individuals showed a significantly increased T cell response to B.1.1.7 ($P$ = 0.0153) and B.1.617.2 ($P$ = 0.0283) peptide pools and N501Y ($P$ = 0.0156) variant peptide compared to the respective Wuhan Hu-1 pools and peptide (Fig. 2B). A cumulative increase in T cell response to the Wuhan Hu-1 spike MEP pool was observed as the number of antigen exposures increased (one to two exposures, $P$ = 0.0003; two to three exposures, $P$ = 0.0294; Fig. 2C). There was a similarly increased T cell response to the B.1.1.7 variant peptide pool (one to two exposures, $P$ = 0.0008; two to three exposures, $P$ = 0.0004; Fig. 2E) depending on number of antigen exposures, indicating a heteroclitic response to variant peptides (*23*) (Fig. 2, D and E).

## VOC mutation alters T cell effector program

Immunization of transgenic mice expressing human HLADRB1*0401 were used to see if any of the variant peptides behave as an altered peptide ligand (APL), eliciting a differential T cell program (*9*). *In silico* analysis of P.1 variant peptides indicated that the majority are not predicted to bind to common HLAII alleles found in the UK population (table S3). The N501Y mutation is predicted to change from strong to weak binding to HLADRB1*0401. IFN$\gamma$ T cell responses in mice primed with Wuhan-Hu-1 peptide pool were ablated for the 501Y variant peptide (found in the B.1.1.7, B.1.351 and P.1 VOCs (Fig. 2F) when presented by HLA-DRB1*0401 ($n$ = 7, $P$ = 0.006). Altered CD4 recognition of the N501Y mutation merits further attention as it is a frequent replacement among reported sequences, comprising a convergent mutational signature shared by alpha, beta and gamma lineages (*24*–*26*). Thus far the lack of T cell responses to 501Y has been described in terms of absence of CD8 recognition (*27*). Our transcriptomic analysis showed an absence of immune effector response to the variant peptide, although induction of the regulatory T cell (Treg) transcription factor, FOXP3, did occur, indicating that the variant peptide may exert an APL effect on T cell function, switching from effector to regulatory (Fig. 2G). This was further confirmed by qPCR for IRF4 and by CD4 expression of FoxP3 protein, indicating that mutant epitopes may subvert T cell activation into a regulatory program (Fig. 2, G to I).

Mice were primed with either B.1.617.2 or Wuhan Hu-1 matched specific peptide pools. In each case, T cells responded on challenge to the pool with which they were primed and not to the heterologous panel ($P$ = 0.0022; Fig. 2J). That is, the T cell repertoire distinguishes VOC mutations, specifically recognizing the B.1.617.2 epitopes. *In silico* analysis of the B.1.617.2 variant peptides indicated that the majority are predicted to bind one or more of the common HLAII alleles found in the UK population (table S4). The D950N mutation is predicted to change from HLADRB1*0401 binding from weak to strong. T cell responses after priming with B.1.617.2 peptide pool were present for the D950N variant peptide, but not after priming with the Wuhan Hu-1 pool ($P$ = 0.0022; Fig. 2J).

## Heterologous B.1.1.7 infection shapes subsequent immunity

We then looked at the impact of infection with B.1.1.7 during the second wave on immune responses in individuals given vaccine expressing Wuhan Hu-1 spike. For this part of the study, an additional 358 HCWs were followed up at 55-57 weeks after recruitment into the study (fig. S1). Of these, 63 had been infected with SARS-CoV-2 during the first Wuhan Hu-1 wave. Fifty-three of previously uninfected HCW (53/296, 18%) were identified by virtue of longitudinal N serology (from baseline to 16-18, 28-30, 42, 54, and 55-57 weeks) as having been newly infected during the B.1.1.7 s wave. Thirty-six (68%) were doubly vaccinated and eight (15%) unvaccinated at the time of follow up (fig. S1 and tables S5 and S6). Five previously infected HCW were reinfected (5/63, 8%) during the second wave. S1 IgG titers were measured at 55-57 weeks (fig. S6B). The median date of a positive SARS-CoV-2 PCR test was December 28$^{th}$, 2020 (IQR, 22d). The B.1.1.7 VOC accounted for 94.7% of SARS-CoV-2 infections in central London in the two weeks leading up to January 2$^{nd}$, 202 (*28*); we thus made the starting assumption that for the majority of HCW infected during the second wave, the infecting strain was B.1.1.7. To support this, we compared the Wuhan Hu-1 and B.1.1.7 nAb IC50 results for unvaccinated ($n$ = 24 and 8), single dose ($n$ = 25 and 9), and two dose vaccinated ($n$ = 24 and 34), HCW infected during the first and second UK waves by Wuhan Hu-1 and B.1.1.7 respectively (fig. S7). In unvaccinated HCWs, those infected during the first wave (fig. S7A, LHS) made nAb IC50 responses against Wuhan Hu-1, but negligible or no nAb IC50 response against B.1.1.7 ($P$ < 0.0001). In contrast, HCW infected as B.1.1.7 peaked (fig. S7A, RHS) made similar nAb IC50 against B.1.1.7 and Wuhan Hu-1 live virus. In Wuhan Hu-1 infected HCW, the Wuhan Hu-1

and B.1.1.7 nAb IC50 increased and remained significantly different after one ($P < 0.0001$) and two ($P < 0.0001$) dose vaccination, but the differential between them decreased (Fig. 3F and fig. S7, B and C).

We set out to investigate the impact of heterologous antigen exposure with B.1.1.7. A confounder in assessing comparative post-vaccination immunity between first wave (Wuhan-Hu-1) infected and second wave (B.1.1.7) infected HCW is that they differ with respect to the infecting strain and in the time interval between infection and vaccination. We hypothesized that observed differences in immunity may relate to recall differences between heterologous spike sequences – immune imprinting (*16–18*), and/or to differences in affinity maturation of the Ab repertoire with time from initial infection. Somatic hypermutation and breadth of neutralizing response is known to improve with time from infection (*29–31*), but also, there may be intrinsic differences between the breadth and hierarchy of neutralizing responses primed by different variants (*32*). We initially compared nAb profiles for neutralization of Wuhan Hu-1 and VOC alpha to delta at a similar number of weeks after PCR confirmed SARS-CoV-2 infection with Wuhan Hu-1 during the first wave and B.1.1.7 during the second wave (all HCW unvaccinated). This data supports the contention that there are qualitatively and quantitatively differential patterns, even matching for timepoint (fig. S9).

After a single vaccine dose, infection-naïve HCW made a similar S1 RBD response to that seen in infection with B.1.1.7. Those HCW that were infected by Wuhan Hu-1 12 months earlier made a 3.9-fold higher S1-RBD response than those infected more recently by the B.1.1.7 variant ($P = 0.004$; Fig. 3A). After two vaccine doses, HCW infected during March 2020 by Wuhan Hu-1 ($n = 70$), having had three homologous antigen exposures, made a 1.5-fold higher S1-RBD response than those encountering three antigen exposures, with one of these being heterologous B.1.1.7 ($n = 36$, $P = 0.0188$; Fig. 3A). The B.1.1.7 infected two dose vaccinated HCW made a similar S1-RBD response to two-dose infection-naïve vaccinees. Infection-naïve vacccinees made a 1.8-fold lower response compared to the Wuhan Hu-1 prior infected group ($n = 241$, $P < 0.0001$; Fig. 3A). The indication is that the phenomenon of enhanced vaccine responses by infection, which has been reproducibly described by us and others (*8*, *9*, *11–14*), is less effective if the infection involves heterologous spike from a VOC.

After two vaccine doses, individuals infected during the Wuhan Hu-1 wave made a higher T cell response to the B.1.1.7 peptide pool than those infected during the B.1.1.7 wave ($P = 0.0412$), arguing for stronger T cell priming in those with three homologous exposures (Fig. 3, B to D).

We next considered the effect of B.1.1.7 infection in single and double vaccination. In two dose vaccinated individuals, B.1.1.7 infection resulted in a different hierarchy of nAb IC50 responses against VOC compared to after Wuhan Hu-1 infection: Wuhan Hu-1>B.1.1.7>B.1.351>P.1>B.1.617.2. In the context of heterologous B.1.1.7 infection and two dose vaccination, the hierarchy changed to B.1.1.7>Wuhan Hu-1>B.1.617.2>P.1>B.1.351 (Fig. 3, E and F). This indicates a process of selective discriminative heterologous imprinting following exposure to B.1.1.7 infection (Fig. 3, E and F, and fig. S8). As predicted for immune imprinting by SARS-CoV-2 variants, first encounter imparts a differential subsequent pattern (*16–18*).

To better understand the relationship between differential nAb IC50 and S1 RBD binding we correlated the two. First we compared the impact of 2 homologous antigen exposures (either 2 dose vaccination [blue, infection-naive] or prior Wuhan Hu-1 infection followed by single-dose vaccination [red, Wuhan Hu-1]) to heterologous exposure through B.1.1.7 infection (single dose vaccination and B.1.1.7 infection [green, B.1.1.7]). Double dose vaccinated infection-naïve HCW (blue) achieved higher neutralization against authentic Wuhan Hu-1 live virus than those with heterologous infection by B.1.1.7 and single-dose vaccination (green) ($P = 0.0036$; Fig. 3G and fig. S10A). Individuals infected by B.1.1.7 and single-dose vaccinated (green) made higher nAb IC50 against B.1.1.7 compared to two dose infection-naïve HCW ($P < 0.0001$) (blue) and Wuhan Hu-1 prior infected single-dose vaccinated HCW ($P < 0.0001$) (red) (Fig. 3G and fig. S10A). Furthermore, neutralization IC50 of B.1.351 live virus was less potent following heterologous B.1.1.7 infection and single dose vaccination (green) ($P = 0.0001$; Fig. 3G and fig. S10A). Following 3 antigen encounters, the impact of heterologous exposure becomes more pronounced. HCW with heterologous infection with B.1.1.7 (green) rather than Wuhan Hu-1 (red) had substantially lower neutralization against authentic Wuhan Hu-1 ($P < 0.0001$), B.1.351 ($P < 0.0001$) and P.1 ($P = 0.0616$) live virus (fig. S10B). Furthermore, double vaccinated HCW with a history of heterologous infection by B.1.1.7 had lower nAb IC50 against B.1.351 than infection-naïve double vaccinated HCW ($P = 0.0007$; fig. S10B). In contrast, double dose vaccinated HCW with a history of heterologous infection with B.1.1.7 (green) had higher nAb IC50 against B.1.617.2 than infection-naïve double vaccinated HCW ($P < 0.0001$; fig. S10B). Thus, neutralization potency achieved was dependent on number of antigen encounters and the presence or absence of heterologous exposure. Ab neutralization is a widely accepted correlate of protection (COP), estimated at a nAb IC50 of >15, and S1 RBD binding an accessible and accepted marker of this (*22*). Our data here show that S1 RBD and VOC S1 RBD titers are not a reliable COP for VOC, especially in the context of selective discriminative heterologous exposure during infection with B.1.1.7. In HCW with heterologous B.1.1.7 exposure, the nAb IC50 of B1.351 was significantly reduced ($P < 0.0001$) and B.1.617.2 significantly increased ($P =$

0.0025) when compared to HCW with homologous Wuhan Hu-1 infection.

### Differential longitudinal immunity after heterologous infection

Next, we considered relative durability of immunity following 3 dose homologous (Wuhan Hu-1 infection plus vaccination) and heterologous (B.1.1.7 infection plus vaccination) antigen compared to two dose vaccinated infection-naïve individuals. S1 RBD Ab levels declined as the number of weeks post second dose increased in both infection-naïve ($r$ = 0.545; $P$ < 0.0001) and prior infected ($r$ = 0.537; $P$ < 0.0001) HCW (Fig. 4, A and B, and tables S1 and S5 to S9). Following a third vaccine dose the S1 RBD binding increased in all three groups to a similar level (Fig. 4C). B.1.1.7 infection (green circles) compared with Wuhan Hu-1 infection (red circles) was associated with lower nAb IC50 against Wuhan Hu-1 ($P$ < 0.0001) and B.1.351 ($P$ < 0.0001) VOC initially at (10d-8 weeks after vaccination) and P.1 ($P$ = 0.0020) 8 weeks later (12-22 weeks after vaccination), indicating differential nAb responses over time depend on the VOC (Fig. 4D). Double vaccinated infection-naïve HCW were at a lower starting value for nAb IC50 against B.1.617.2 compared to B.1.1.7 ($P$ < 0.0001) and Wuhan Hu-1 ($P$ = 0.0462) infected HCW (Fig. 3H and fig. S10B). The nAb IC50 against authentic Wuhan Hu-1 and all four VOC (B.1.1.7, B.1.351, P.1, and B.1.617.2) fell differentially depending on the VOC being neutralized and SARS-CoV-2 infection history (Fig. 4E).

In two dose vaccinated infection-naïve HCW, nAb IC50 against authentic Wuhan Hu-1 and all four of the VOCs fell significantly over 18 weeks (Wuhan Hu-1, 26-fold, $P$ < 0.0001; B.1.1.7, 18-fold, $P$ < 0.0001; B.1.351, 29-fold, $P$ < 0.0001; P.1. 26-fold, $P$ < 0.0001; B.1.617.2 233-fold, $P$ < 0.0001). This was most marked for B.1.617.2 live virus neutralization where by 21 weeks after the second dose it was zero for 21/27 (78%) of HCW tested (median 0, IQR 0, $n$ = 27) (Fig. 4E). For two dose vaccinated prior Wuhan Hu-1 infected HCW, there was a significantly and less pronounced fall in nAb IC50 against authentic Wuhan Hu-1 and three of the VOCs over 18 weeks (Wuhan Hu-1, 5-fold, $P$ = 0.0003; B.1.1.7, 7-fold, $P$ < 0.0001; B.1.351, 4-fold, $P$ = 0.0042; P.1. 10-fold, $P$ = 0.0003). By contrast, in the presence of heterologous infection with B.1.1.7 and two dose vaccination, nAb IC50 fell significantly over 18 weeks for some variants (Wuhan Hu-1, 6-fold, $P$ = 0.0002; B.1.1.7 13-fold, $P$ < 0.0001; P.1 7-fold, $P$ < 0.0001; and B.1.617.2 41-fold, $P$ < 0.0001) while nAb IC50 against B.1.351 did not ($P$ = 0.4304).

T cell responses against spike MEP pool 21 weeks after the second dose were higher in two dose vaccinated HCW that were infected with B.1.1.7 than two dose vaccinated infection-naïve HCW ($P$ = 0.0304; Fig. 4F).

MBC frequencies against the Wuhan Hu-1 S1 and S1 containing the B.1.617.2 mutations were equivalent twenty-one weeks after second dose vaccination (Fig. 4G). Two dose vaccinated B.1.1.7 infected HCW made higher frequency responses against S1 containing the B.1.617.2 mutations than two dose vaccinated infection-naïve HCW ($P$ = 0.0138; Fig. 4H). MBC frequencies in two dose vaccinated infection-naïve HCW fell over 18 weeks ($n$ = 11, $P$ = 0.0098) while those from Wuhan Hu-1 prior infected HCW were sustained ($n$ = 4; Fig. 4I).

### Immune parameters associated with B.1.617.2 breakthrough infection

At the time of the 71-72 and 83-84 week recruitments B.1.617.2 VOC infections accounted for 97.8% and 99.9% of UK SARS-CoV-2 infections respectively (*26*). We identified 6/80 (8%) and 14/74 (19%) B.1.617.2 breakthrough infections in the double vaccinated HCW. Ten were previously infection-naïve, four had prior Wuhan Hu-1 infection and six were infected by B.1.1.7 (tables S8 and S10). PCR positive confirmed B.1.617.2 breakthrough infections occurred in the context of S1 RBD Ab levels ranging 1,110 to 29,308 U/ml (median = 9,010, IQR 13,650) 2-3 weeks after the second vaccine dose. We evaluated the S1 RBD, nAb, T cell and MBC data in these double vaccinated HCW before (purple circle) and after (lilac circle) their B.1.617.2 breakthrough infection (fig. S11, A to J). Analysis showed that both prior infected and infection-naïve HCW making S1 RBD Ab responses (20d post second dose) of over 1,100 U/ml had become infected and breakthrough infection was not specifically associated with being a low responder (fig. S11, A and B). Relatively potent nAb IC50 at 20d after second vaccine dose had fallen 12 weeks later. This fall was more pronounced in the infection-naïve double vaccinated HCW (fig. S11, D and E). For B.1.617.2 the nAb IC50 fell to zero in two dose vaccinated infection-naïve HCW (fig. S11, D and G). B.1.617.2 breakthrough infections have been linked to low nAb responses (*33*). Current estimates for COP apply poorly in the case of B.1.617.2 (*22*). In the cases reported here, breakthrough infections occurred in the face of good prior S1 RBD Ab titers, nAb IC50s and MBC frequency 20d after second vaccine dose, but responses fell in the subsequent 18 weeks especially in the infection naïve two dose vaccinated. Initial immune responses poorly predicted protection against B.1.617.2 breakthrough infection (fig. S11, A to J).

### Discussion

The new challenge in a world facing diverse VOC is to understand both cross-neutralization of these by first-generation spike vaccines, but also, how protective immunity is differentially shaped in those who have had infection by the different VOC (*34*). These real-life issues of immune imprinting have implications for optimal design of second-generation variant

spike vaccine boosters in parts of the world experiencing different VOCs, or in assessing heterogeneity in the quantity, quality, and durability of immune protection in those who have experienced different combinations of infecting and vaccinating sequences. Infection by B.1.1.7 had different features to Wuhan Hu-1 in terms of protection against VOC, evident in the response to infection and in the differential impact of vaccine-boosting previously described (*8*, *9*, *11–14*). Differential skewing of the immune repertoire may occur if the prime/boost is through B.1.1.7, since epitope modification at DY144 and N501Y changes the nAb repertoire (*4*, *35*). We found a differential hierarchy of cross-neutralization to VOC after natural infection, one or two vaccine doses, depending on the infecting strain. That different immune priming by B.1.1.7 is likely a consequence of heterologous virus per se and not of a shorter timespan for affinity maturation is supported by data from Israel, confirming that susceptibility grows with time and with declining Ab levels (*36*). Vaccine programs currently use prototypic Wuhan Hu-1 sequence against a background of infection by different VOC predominating in different parts of the world. Decoding the differential breadth of VOC neutralizing response ensuing from diverse priming combinations will affect which variant spike sequences may best serve in second generation vaccines. This appears a more complex choice than simply opting for the most concerning variant at any given time. Since heterologous combinations can confer a diminished response against other variants, there may be a case for sticking with the Wuhan Hu-1 sequence in booster vaccinations in the first instance in the face of unknown future VOCs, or for improved efforts to define vaccines based on optimization of common, conserved, neutralizing epitopes (*37*). In any case, the inference from this cohort is that populations infected during waves of different variants carry distinct immune memory, with implications for differential protection against future VOCs.

## Methods summary

Detailed materials and methods are reported in the supplementary materials. Recruitment of 731 HCW into the COVIDsortium cohort followed longitudinally with weekly self-reported symptom diaries, SARS-CoV-2 PCR, N and S1 RBD serology for 16-weeks from the start of the first UK wave has been described (*5–10*). This enabled a cross-sectional case controlled sub-study of 136 HCW recruited 16-18 weeks after the March 2020 UK lockdown that reported discordant neutralizing antibody and T cell responses in SARS-CoV-2 natural infection during the 1st wave (Wuhan Hu-1) (*6*). A cross-sectional, case-controlled vaccine sub-study cohort of 51 HCW at 22d after first BNT162b2 dose described vaccine immunity in HCW with ($n$ = 25) and without ($n$ = 26) prior SARS-CoV-2 infection during the Wuhan Hu-1 wave (*8*, *9*). The current sub-study includes longitudinal follow-up of this previously published vaccine sub-study cohort ($n$ = 51) at 20d (IQR, 7) after second BNT162b2 dose plus an additional 358 HCW, 53 of whom were infected with B.1.1.7 during the second UK wave. At 71-72 weeks, 80 2-dose vaccinated HCW were re-recruited 18-21 weeks after their second dose for longitudinal follow up of SARS-CoV-2 infection-naïve ($n$ = 27) or prior-infected HCW infected during the Wuhan Hu-1 UK wave ($n$ = 31) or the second B.1.1.7 UK wave ($n$ = 22). At 83-84 weeks, 74 previously 2-dose vaccinated HCW were re-recruited 30-33 weeks after their second dose that were infection naïve ($n$ = 30) or infected during the Wuhan Hu-1 ($n$ = 18) or B.1.1.7 wave ($n$ = 19). Sixty-seven (91%) were within a median of 18d (IQR, 12) after their third BNT162b2 dose. HCW with breakthrough infection by B.1.617.2 were identified by PCR and N serology. Peripheral blood mononuclear cells (PBMC) and serum samples were prepared and cryopreserved as previously described (*6*, *9*). Anti-nucleocapsid (COI ≥ 1.0, positive) and anti-spike antibody (≥0.8 U/ml, positive) detection Ab testing was conducted at UK Health Security Agency (UKHSA) using the Roche cobas®e801 analyser. Recombinant proteins were used in ELISAs looking at the VOC S1 RBD responses: SARS-CoV-2 Spike Glycoprotein (S1) RBD, SARS-CoV-2 (N501Y Mutant), (501Y.V2: K417N, E484K, N501Y), (B.1.1.28: K417T, E484K, N501Y) and (B.1.617.2: L452R, T478K) Spike Glycoprotein (S1) RBDs derived from Wuhan Hu-1, B.1.1.7, B.1.351, P.1 and B.1.617.2 VOC respectively. T cell experiments peptide panels included spike mapped epitope pool (MEP) comprised of a pool of eighteen 12-20mer peptide epitopes (*6*, *9*) and VOC pools contained peptides from the B.1.1.7, B.1.351, P.1 and B.1.617.2 sequences and their respective Wuhan Hu-1 sequence (table S2). IFN$\gamma$-T cell ELISpots and MBC ELISpots were performed as previously described (*6*, *9*). MBC ELISpot plates were coated with PBS, purified anti-human IgG MT91/145, SARS-CoV-2 S1 spike, E484K, K417N, N501Y spike or T19R, G142D, del 156-157, R158G, L452R, T478K, D614G, P681R spike. *In silico* predictions of HLA-DRB1/peptide-binding utilized NetMHCIIpan-4.0 (*9*, *38*, *39*). Studies using HLAII transgenics (DRB1*0401) were performed as previously described (*9*, *40*, *41*). For transcriptomic and flow cytometry analysis, mouse lymph node cells were cultured with no peptide, wild-type or variant N501Y peptides, then, at 24h, cells were harvested and lysed for RNA extraction or stained for flow cytometry. SARS-CoV-2 microneutralization assays were carried out as described previously using VeroE6 cells (*6*, *9*). Participant sera were incubated with $3x10^4$ FFU SARS-CoV-2 (Wuhan Hu-1, B.1.1.7, B.1.351, P.1, or B.1617.2 isolates of SARS-CoV-2 live virus).

### REFERENCES AND NOTES

1. D. A. Collier, I. A. T. M. Ferreira, P. Kotagiri, R. P. Datir, E. Y. Lim, E. Touizer, B. Meng, A. Abdullahi, CITIID-NIHR BioResource COVID-19 Collaboration, A. Elmer, N. Kingston, B. Graves, E. Le Gresley, D. Caputo, L. Bergamaschi, K. G. C. Smith, J. R. Bradley, L. Ceron-Gutierrez, P. Cortes-Acevedo, G. Barcenas-Morales, M. A. Linterman, L. E. McCoy, C. Davis, E. Thomson, P. A. Lyons, E. McKinney, R.

Doffinger, M. Wills, R. K. Gupta, Age-related immune response heterogeneity to SARS-CoV-2 vaccine BNT162b2. *Nature* **596**, 417–422 (2021). doi:10.1038/s41586-021-03739-1 Medline
2. E. C. Wall, M. Wu, R. Harvey, G. Kelly, S. Warchal, C. Sawyer, R. Daniels, P. Hobson, E. Hatipoglu, Y. Ngai, S. Hussain, J. Nicod, R. Goldstone, K. Ambrose, S. Hindmarsh, R. Beale, A. Riddell, S. Gamblin, M. Howell, G. Kassiotis, V. Libri, B. Williams, C. Swanton, S. Gandhi, D. L. Bauer, Neutralising antibody activity against SARS-CoV-2 VOCs B.1.617.2 and B.1.351 by BNT162b2 vaccination. *Lancet* **397**, 2331–2333 (2021). doi:10.1016/S0140-6736(21)01290-3 Medline
3. D. Planas, D. Veyer, A. Baidaliuk, I. Staropoli, F. Guivel-Benhassine, M. M. Rajah, C. Planchais, F. Porrot, N. Robillard, J. Puech, M. Prot, F. Gallais, P. Gantner, A. Velay, J. Le Guen, N. Kassis-Chikhani, D. Edriss, L. Belec, A. Seve, L. Courtellemont, H. Péré, L. Hocqueloux, S. Fafi-Kremer, T. Prazuck, H. Mouquet, T. Bruel, E. Simon-Lorière, F. A. Rey, O. Schwartz, Reduced sensitivity of SARS-CoV-2 variant Delta to antibody neutralization. *Nature* **596**, 276–280 (2021). doi:10.1038/s41586-021-03777-9 Medline
4. W. T. Harvey, A. M. Carabelli, B. Jackson, R. K. Gupta, E. C. Thomson, E. M. Harrison, C. Ludden, R. Reeve, A. Rambaut, S. J. Peacock, D. L. Robertson; COVID-19 Genomics UK (COG-UK) Consortium, SARS-CoV-2 variants, spike mutations and immune escape. *Nat. Rev. Microbiol.* **19**, 409–424 (2021). doi:10.1038/s41579-021-00573-0 Medline
5. T. A. Treibel, C. Manisty, M. Burton, Á. McKnight, J. Lambourne, J. B. Augusto, X. Couto-Parada, T. Cutino-Moguel, M. Noursadeghi, J. C. Moon, COVID-19: PCR screening of asymptomatic health-care workers at London hospital. *Lancet* **395**, 1608–1610 (2020). doi:10.1016/S0140-6736(20)31100-4 Medline
6. C. J. Reynolds, L. Swadling, J. M. Gibbons, C. Pade, M. P. Jensen, M. O. Diniz, N. M. Schmidt, D. K. Butler, O. E. Amin, S. N. L. Bailey, S. M. Murray, F. P. Pieper, S. Taylor, J. Jones, M. Jones, W. J. Lee, J. Rosenheim, A. Chandran, G. Joy, C. Di Genova, N. Temperton, J. Lambourne, T. Cutino-Moguel, M. Andiapen, M. Fontana, A. Smit, A. Semper, B. O'Brien, B. Chain, T. Brooks, C. Manisty, T. Treibel, J. C. Moon, M. Noursadeghi, D. M. Altmann, M. K. Maini, Á. McKnight, R. J. Boyton; COVIDsortium investigators; COVIDsortium immune correlates network, Discordant neutralizing antibody and T cell responses in asymptomatic and mild SARS-CoV-2 infection. *Sci. Immunol.* **5**, eabf3698 (2020). doi:10.1126/sciimmunol.abf3698 Medline
7. C. Manisty, T. A. Treibel, M. Jensen, A. Semper, G. Joy, R. K. Gupta, T. Cutino-Moguel, M. Andiapen, J. Jones, S. Taylor, A. Otter, C. Pade, J. Gibbons, J. Lee, J. Bacon, S. Thomas, C. Moon, M. Jones, D. Williams, J. Lambourne, M. Fontana, D. M. Altmann, R. Boyton, M. Maini, A. McKnight, B. Chain, M. Noursadeghi, J. C. Moon, Time series analysis and mechanistic modelling of heterogeneity and sero-reversion in antibody responses to mild SARS-CoV-2 infection. *EBioMedicine* **65**, 103259 (2021). doi:10.1016/j.ebiom.2021.103259 Medline
8. C. Manisty, A. D. Otter, T. A. Treibel, Á. McKnight, D. M. Altmann, T. Brooks, M. Noursadeghi, R. J. Boyton, A. Semper, J. C. Moon, Antibody response to first BNT162b2 dose in previously SARS-CoV-2-infected individuals. *Lancet* **397**, 1057–1058 (2021). doi:10.1016/S0140-6736(21)00501-8 Medline
9. C. J. Reynolds, C. Pade, J. M. Gibbons, D. K. Butler, A. D. Otter, K. Menacho, M. Fontana, A. Smit, J. E. Sackville-West, T. Cutino-Moguel, M. K. Maini, B. Chain, M. Noursadeghi, ; UK COVIDsortium Immune Correlates Network, T. Brooks, A. Semper, C. Manisty, T. A. Treibel, J. C. Moon, UK COVIDsortium Investigators, A. M. Valdes, Á. McKnight, D. M. Altmann, R. Boyton, Prior SARS-CoV-2 infection rescues B and T cell responses to variants after first vaccine dose. *Science* **372**, eabh1282 (2021). doi:10.1126/science.abh1282 Medline
10. R. K. Gupta, J. Rosenheim, L. C. Bell, A. Chandran, J. A. Guerra-Assuncao, G. Pollara, M. Whelan, J. Artico, G. Joy, H. Kurdi, D. M. Altmann, R. J. Boyton, M. K. Maini, A. McKnight, J. Lambourne, T. Cutino-Moguel, C. Manisty, T. A. Treibel, J. C. Moon, B. M. Chain, M. Noursadeghi, COVIDsortium Investigators, Blood transcriptional biomarkers of acute viral infection for detection of pre-symptomatic SARS-CoV-2 infection: A nested, case-control diagnostic accuracy study. *Lancet Microbe* **2**, e508–e517 (2021). doi:10.1016/S2666-5247(21)00146-4 Medline
11. F. Krammer, K. Srivastava, H. Alshammary, A. A. Amoako, M. H. Awawda, K. F. Beach, M. C. Bermúdez-González, D. A. Bielak, J. M. Carreño, R. L. Chernet, L. Q. Eaker, E. D. Ferreri, D. L. Floda, C. R. Gleason, J. Z. Hamburger, K. Jiang, G. Kleiner, D. Jurczyszak, J. C. Matthews, W. A. Mendez, I. Nabeel, L. C. F. Mulder, A. J. Raskin, K. T. Russo, A. T. Salimbangon, M. Saksena, A. S. Shin, G. Singh, L. A. Sominsky, D. Stadlbauer, A. Wajnberg, V. Simon, Antibody responses in seropositive persons after a single dose of SARS-CoV-2 mRNA vaccine. *N. Engl. J. Med.* **384**, 1372–1374 (2021). doi:10.1056/NEJMc2101667 Medline
12. L. Stamatatos, J. Czartoski, Y. H. Wan, L. J. Homad, V. Rubin, H. Glantz, M. Neradilek, E. Seydoux, M. F. Jennewein, A. J. MacCamy, J. Feng, G. Mize, S. C. De Rosa, A. Finzi, M. P. Lemos, K. W. Cohen, Z. Moodie, M. J. McElrath, A. T. McGuire, mRNA vaccination boosts cross-variant neutralizing antibodies elicited by SARS-CoV-2 infection. *Science* **372**, eabg9175 (2021). doi:10.1126/science.abg9175 Medline
13. R. R. Goel, S. A. Apostolidis, M. M. Painter, D. Mathew, A. Pattekar, O. Kuthuru, S. Gouma, P. Hicks, W. Meng, A. M. Rosenfeld, S. Dysinger, K. A. Lundgreen, L. Kuri-Cervantes, S. Adamski, A. Hicks, S. Korte, D. A. Oldridge, A. E. Baxter, J. R. Giles, M. E. Weirick, C. M. McAllister, J. Dougherty, S. Long, K. D'Andrea, J. T. Hamilton, M. R. Betts, E. T. Luning Prak, P. Bates, S. E. Hensley, A. R. Greenplate, E. J. Wherry, Distinct antibody and memory B cell responses in SARS-CoV-2 naïve and recovered individuals after mRNA vaccination. *Sci. Immunol.* **6**, eabi6950 (2021). doi:10.1126/sciimmunol.abi6950 Medline
14. J. E. Ebinger, J. Fert-Bober, I. Printsev, M. Wu, N. Sun, J. C. Prostko, E. C. Frias, J. L. Stewart, J. E. Van Eyk, J. G. Braun, S. Cheng, K. Sobhani, Antibody responses to the BNT162b2 mRNA vaccine in individuals previously infected with SARS-CoV-2. *Nat. Med.* **27**, 981–984 (2021). doi:10.1038/s41591-021-01325-6 Medline
15. P. S. Arunachalam, M. K. D. Scott, T. Hagan, C. Li, Y. Feng, F. Wimmers, L. Grigoryan, M. Trisal, V. V. Edara, L. Lai, S. E. Chang, A. Feng, S. Dhingra, M. Shah, A. S. Lee, S. Chinthrajah, S. B. Sindher, V. Mallajosyula, F. Gao, N. Sigal, S. Kowli, S. Gupta, K. Pellegrini, G. Tharp, S. Maysel-Auslender, S. Hamilton, H. Aoued, K. Hrusovsky, M. Roskey, S. E. Bosinger, H. T. Maecker, S. D. Boyd, M. M. Davis, P. J. Utz, M. S. Suthar, P. Khatri, K. C. Nadeau, B. Pulendran, Systems vaccinology of the BNT162b2 mRNA vaccine in humans. *Nature* **596**, 410–416 (2021). doi:10.1038/s41586-021-03791-x Medline
16. A. Zhang, H. D. Stacey, C. E. Mullarkey, M. S. Miller, Original antigenic sin: How first exposure shapes lifelong anti-influenza virus immune responses. *J. Immunol.* **202**, 335–340 (2019). doi:10.4049/jimmunol.1801149 Medline
17. T. Aydillo, A. Rombauts, D. Stadlbauer, S. Aslam, G. Abelenda-Alonso, A. Escalera, F. Amanat, K. Jiang, F. Krammer, J. Carratala, A. García-Sastre, Immunological imprinting of the antibody response in COVID-19 patients. *Nat. Commun.* **12**, 3781 (2021). doi:10.1038/s41467-021-23977-1 Medline
18. A. K. Wheatley, A. Fox, H. X. Tan, J. A. Juno, M. P. Davenport, K. Subbarao, S. J. Kent, Immune imprinting and SARS-CoV-2 vaccine design. *Trends Immunol.* **42**, 956–959 (2021). doi:10.1016/j.it.2021.09.001 Medline
19. N. Kamar, F. Abravanel, O. Marion, C. Couat, J. Izopet, A. Del Bello, Three doses of an mRNA Covid-19 vaccine in solid-organ transplant recipients. *N. Engl. J. Med.* **385**, 661–662 (2021). doi:10.1056/NEJMc2108861 Medline
20. A. Del Bello, F. Abravanel, O. Marion, C. Couat, L. Esposito, L. Lavayssière, J. Izopet, N. Kamar, Efficiency of a boost with a third dose of anti-SARS-CoV-2 messenger RNA-based vaccines in solid organ transplant recipients. *Am. J. Transplant.* ajt.16775 (2021). doi:10.1111/ajt.16775 Medline
21. D. M. Altmann, R. J. Boyton, R. Beale, Immunity to SARS-CoV-2 variants of concern. *Science* **371**, 1103–1104 (2021). doi:10.1126/science.abg7404 Medline
22. D. S. Khoury, D. Cromer, A. Reynaldi, T. E. Schlub, A. K. Wheatley, J. A. Juno, K. Subbarao, S. J. Kent, J. A. Triccas, M. P. Davenport, Neutralizing antibody levels are highly predictive of immune protection from symptomatic SARS-CoV-2 infection. *Nat. Med.* **27**, 1205–1211 (2021). doi:10.1038/s41591-021-01377-8 Medline
23. A. M. Solinger, M. E. Ultee, E. Margoliash, R. H. Schwartz, T-lymphocyte response to cytochrome c. I. Demonstration of a T-cell heteroclitic proliferative response and identification of a topographic antigenic determinant on pigeon cytochrome c whose immune recognition requires two complementing major histocompatibility complex-linked immune response genes. *J. Exp. Med.* **150**, 830–848 (1979). doi:10.1084/jem.150.4.830 Medline
24. D. P. Martin, S. Weaver, H. Tegally, J. E. San, S. D. Shank, E. Wilkinson, A. G. Lucaci, J. Giandhari, S. Naidoo, Y. Pillay, L. Singh, R. J. Lessells, R. K. Gupta, J. O. Wertheim, A. Nekturenko, B. Murrell, G. W. Harkins, P. Lemey, O. A. MacLean, D. L. Robertson, T. de Oliveira, S. L. Kosakovsky Pond; NGS-SA; COVID-19 Genomics UK (COG-UK), The emergence and ongoing convergent evolution of the SARS-

CoV-2 N501Y lineages. *Cell* **184**, 5189–5200.e7 (2021). doi:10.1016/j.cell.2021.09.003 Medline
25. L. Lu, A. W. Chu, R. R. Zhang, W. M. Chan, J. D. Ip, H. W. Tsoi, L. L. Chen, J. P. Cai, D. C. Lung, A. R. Tam, Y. S. Yau, M. Y. Kwan, W. K. To, O. T. Tsang, L. L. Lee, H. Yi, T. C. Ip, R. W. Poon, G. K. Siu, B. W. Mok, V. C. Cheng, K. H. Chan, K. Y. Yuen, I. F. Hung, K. K. To, The impact of spike N501Y mutation on neutralizing activity and RBD binding of SARS-CoV-2 convalescent serum. *EBioMedicine* **71**, 103544 (2021). doi:10.1016/j.ebiom.2021.103544 Medline
26. GISAID, "Tracking of variants" (2021); https://www.gisaid.org/hcov19-variants/
27. T. M. Snyder, R. M. Gittelman, M. Klinger, D. H. May, E. J. Osborne, R. Taniguchi, H. J. Zahid, I. M. Kaplan, J. N. Dines, M. T. Noakes, R. Pandya, X. Chen, S. Elasady, E. Svejnoha, P. Ebert, M. W. Pesesky, P. De Almeida, H. O'Donnell, Q. DeGottardi, G. Keitany, J. Lu, A. Vong, R. Elyanow, P. Fields, J. Greissl, L. Baldo, S. Semprini, C. Cerchione, F. Nicolini, M. Mazza, O. M. Delmonte, K. Dobbs, R. Laguna-Goya, G. Carreño-Tarragona, S. Barrio, L. Imberti, A. Sottini, E. Quiros-Roldan, C. Rossi, A. Biondi, L. R. Bettini, M. D'Angio, P. Bonfanti, M. F. Tompkins, C. Alba, C. Dalgard, V. Sambri, G. Martinelli, J. D. Goldman, J. R. Heath, H. C. Su, L. D. Notarangelo, E. Paz-Artal, J. Martinez-Lopez, J. M. Carlson, H. S. Robins, Magnitude and dynamics of the T-cell response to SARS-CoV-2 Infection at both individual and population levels. medRxiv 2020.07.31.20165647 [Preprint] (2020); https://doi.org/10.1101/2020.07.31.20165647.
28. Wellcome Sanger Institute, "COVID-19 genomic surveillance" (2021); https://covid19.sanger.ac.uk/lineages/raw.
29. F. Muecksch, Y. Weisblum, C. O. Barnes, F. Schmidt, D. Schaefer-Babajew, Z. Wang, J. C. C. Lorenzi, A. I. Flyak, A. T. DeLaitsch, K. E. Huey-Tubman, S. Hou, C. A. Schiffer, C. Gaebler, J. Da Silva, D. Poston, S. Finkin, A. Cho, M. Cipolla, T. Y. Oliveira, K. G. Millard, V. Ramos, A. Gazumyan, M. Rutkowska, M. Caskey, M. C. Nussenzweig, P. J. Bjorkman, T. Hatziioannou, P. D. Bieniasz, Affinity maturation of SARS-CoV-2 neutralizing antibodies confers potency, breadth, and resilience to viral escape mutations. *Immunity* **54**, 1853–1868.e7 (2021). doi:10.1016/j.immuni.2021.07.008 Medline
30. Z. Wang, F. Muecksch, D. Schaefer-Babajew, S. Finkin, C. Viant, C. Gaebler, H. H. Hoffmann, C. O. Barnes, M. Cipolla, V. Ramos, T. Y. Oliveira, A. Cho, F. Schmidt, J. Da Silva, E. Bednarski, L. Aguado, J. Yee, M. Daga, M. Turroja, K. G. Millard, M. Jankovic, A. Gazumyan, Z. Zhao, C. M. Rice, P. D. Bieniasz, M. Caskey, T. Hatziioannou, M. C. Nussenzweig, Naturally enhanced neutralizing breadth against SARS-CoV-2 one year after infection. *Nature* **595**, 426–431 (2021). doi:10.1038/s41586-021-03696-9 Medline
31. C. Gaebler, Z. Wang, J. C. C. Lorenzi, F. Muecksch, S. Finkin, M. Tokuyama, A. Cho, M. Jankovic, D. Schaefer-Babajew, T. Y. Oliveira, M. Cipolla, C. Viant, C. O. Barnes, Y. Bram, G. Breton, T. Hägglöf, P. Mendoza, A. Hurley, M. Turroja, K. Gordon, K. G. Millard, V. Ramos, F. Schmidt, Y. Weisblum, D. Jha, M. Tankelevich, G. Martinez-Delgado, J. Yee, R. Patel, J. Dizon, C. Unson-O'Brien, I. Shimeliovich, D. F. Robbiani, Z. Zhao, A. Gazumyan, R. E. Schwartz, T. Hatziioannou, P. J. Bjorkman, S. Mehandru, P. D. Bieniasz, M. Caskey, M. C. Nussenzweig, Evolution of antibody immunity to SARS-CoV-2. *Nature* **591**, 639–644 (2021). doi:10.1038/s41586-021-03207-w Medline
32. N. Faulkner, K. W. Ng, M. Y. Wu, R. Harvey, M. Margaritis, S. Paraskevopoulou, C. Houlihan, S. Hussain, M. Greco, W. Bolland, S. Warchal, J. Heaney, H. Rickman, M. Spyer, D. Frampton, M. Byott, T. de Oliveira, A. Sigal, S. Kjaer, C. Swanton, S. Gandhi, R. Beale, S. J. Gamblin, J. W. McCauley, R. S. Daniels, M. Howell, D. Bauer, E. Nastouli, G. Kassiotis, Reduced antibody cross-reactivity following infection with B.1.1.7 than with parental SARS-CoV-2 strains. *eLife* **10**, e69317 (2021). doi:10.7554/eLife.69317 Medline
33. M. Bergwerk, T. Gonen, Y. Lustig, S. Amit, M. Lipsitch, C. Cohen, M. Mandelboim, E. G. Levin, C. Rubin, V. Indenbaum, I. Tal, M. Zavitan, N. Zuckerman, A. Bar-Chaim, Y. Kreiss, G. Regev-Yochay, Covid-19 breakthrough infections in vaccinated health care workers. *N. Engl. J. Med.* **385**, 1474–1484 (2021). doi:10.1056/NEJMoa2109072 Medline
34. T. Moyo-Gwete, M. Madzivhandila, Z. Makhado, F. Ayres, D. Mhlanga, B. Oosthuysen, B. E. Lambson, P. Kgagudi, H. Tegally, A. Iranzadeh, D. Doolabh, L. Tyers, L. R. Chinhoyi, M. Mennen, S. Skelem, G. Marais, C. K. Wibmer, J. N. Bhiman, V. Ueckermann, T. Rossouw, M. Boswell, T. de Oliveira, C. Williamson, W. A. Burgers, N. Ntusi, L. Morris, P. L. Moore, Cross-reactive neutralizing antibody responses elicited by SARS-CoV-2 501Y.V2 (B.1.351). *N. Engl. J. Med.* **384**, 2161–2163 (2021). doi:10.1056/NEJMc2104192 Medline
35. M. McCallum, A. De Marco, F. A. Lempp, M. A. Tortorici, D. Pinto, A. C. Walls, M. Beltramello, A. Chen, Z. Liu, F. Zatta, S. Zepeda, J. di Iulio, J. E. Bowen, M. Montiel-Ruiz, J. Zhou, L. E. Rosen, S. Bianchi, B. Guarino, C. S. Fregni, R. Abdelnabi, S. C. Foo, P. W. Rothlauf, L. M. Bloyet, F. Benigni, E. Cameroni, J. Neyts, A. Riva, G. Snell, A. Telenti, S. P. J. Whelan, H. W. Virgin, D. Corti, M. S. Pizzuto, D. Veesler, N-terminal domain antigenic mapping reveals a site of vulnerability for SARS-CoV-2. *Cell* **184**, 2332–2347.e16 (2021). doi:10.1016/j.cell.2021.03.028 Medline
36. Y. Goldberg, M. Mandel, Y. M. Bar-On, O. Bodenheimer, L. Freedman, E. J. Haas, R. Milo, S. Alroy-Preis, N. Ash, A. Huppert, Waning immunity after the BNT162b2 vaccine in Israel. *N. Engl. J. Med.* NEJMoa2114228 (2021). doi:10.1056/NEJMoa2114228 Medline
37. L. A. VanBlargan, L. J. Adams, Z. Liu, R. E. Chen, P. Gilchuk, S. Raju, B. K. Smith, H. Zhao, J. B. Case, E. S. Winkler, B. M. Whitener, L. Droit, I. D. Aziati, T. L. Bricker, A. Joshi, P. Y. Shi, A. Creanga, A. Pegu, S. A. Handley, D. Wang, A. C. M. Boon, J. E. Crowe Jr., S. P. J. Whelan, D. H. Fremont, M. S. Diamond, A potently neutralizing SARS-CoV-2 antibody inhibits variants of concern by utilizing unique binding residues in a highly conserved epitope. *Immunity* **54**, 2399–2416.e6 (2021). doi:10.1016/j.immuni.2021.08.016 Medline
38. D. M. Altmann, C. J. Reynolds, R. J. Boyton, SARS-CoV-2 variants: Subversion of antibody response and predicted impact on T cell recognition. *Cell Rep. Med.* **2**, 100286 (2021). doi:10.1016/j.xcrm.2021.100286 Medline
39. B. Reynisson, B. Alvarez, S. Paul, B. Peters, M. Nielsen, NetMHCpan-4.1 and NetMHCIIpan-4.0: Improved predictions of MHC antigen presentation by concurrent motif deconvolution and integration of MS MHC eluted ligand data. *Nucleic Acids Res.* **48** (W1), W449–W454 (2020). doi:10.1093/nar/gkaa379 Medline
40. K. J. Quigley, C. J. Reynolds, A. Goudet, E. J. Raynsford, R. Sergeant, A. Quigley, S. Worgall, D. Bilton, R. Wilson, M. R. Loebinger, B. Maillere, D. M. Altmann, R. J. Boyton, Chronic infection by mucoid *Pseudomonas aeruginosa* associated with dysregulation in T-cell immunity to outer membrane porin F. *Am. J. Respir. Crit. Care Med.* **191**, 1250–1264 (2015). doi:10.1164/rccm.201411-1995OC Medline
41. C. Reynolds, A. Goudet, K. Jenjaroen, M. Sumonwiriya, D. Rinchai, J. Musson, S. Overbeek, J. Makinde, K. Quigley, J. Manji, N. Spink, P. Yos, V. Wuthiekanun, G. Bancroft, J. Robinson, G. Lertmemongkolchai, S. Dunachie, B. Maillere, M. Holden, D. Altmann, R. Boyton, T cell immunity to the alkyl hydroperoxide reductase of *Burkholderia pseudomallei*: A correlate of disease outcome in acute melioidosis. *J. Immunol.* **194**, 4814–4824 (2015). doi:10.4049/jimmunol.1402862 Medline
42. K. Tao, P. L. Tzou, J. Nouhin, R. K. Gupta, T. de Oliveira, S. L. Kosakovsky Pond, D. Fera, R. W. Shafer, The biological and clinical significance of emerging SARS-CoV-2 variants. *Nat. Rev. Genet.* **22**, 757–773 (2021). doi:10.1038/s41576-021-00408-x Medline
43. J. Zou, X. Xie, C. R. Fontes-Garfias, K. A. Swanson, I. Kanevsky, K. Tompkins, M. Cutler, D. Cooper, P. R. Dormitzer, P. Y. Shi, The effect of SARS-CoV-2 D614G mutation on BNT162b2 vaccine-elicited neutralization. *NPJ Vaccines* **6**, 44 (2021). doi:10.1038/s41541-021-00313-8 Medline

## ACKNOWLEDGMENTS

The authors thank HCW participants for participating in the study and the research teams involved in recruitment, obtaining consent, and sampling the HCW participants. The COVIDsortium Healthcare Workers bioresource is approved by the ethical committee of UK National Research Ethics Service (20/SC/0149) and registered on ClinicalTrials.gov (NCT04318314). The study conforms to the principles of the Helsinki Declaration, and all subjects provided written informed consent. The SARS-CoV-2 Wuhan Hu-1 Human 2019-nCoV Isolate EVA catalog code 026V-03883 was obtained from European Virus Archive Global. (EVAg), Charité - Universitätsmedizin Berlin. The SARS-CoV-2 B.1.1.7 isolate was obtained from NIBSC, thanks to the contribution of PHE Porton Down and Dr Simon Funnell. The nCoV19 isolate/UK ex South African/2021 lineage B.1.351 EVA catalog code 04V-04071 was obtained from EVAg, PHE Porton Down. P.1 and B.1.617.2 isolates were purchased from EVAg. This work is licensed under a Creative Commons Attribution 4.0 International (CC BY 4.0) license, which permits unrestricted use, distribution, and reproduction in any medium, provided the original work is properly cited. To view a copy of this license, visit https://creativecommons.org/licenses/by/4.0/. This license does not apply to

figures/photos/artwork or other content included in the article that is credited to a third party; obtain authorization from the rights holder before using such material. **Funding**: RJB and DMA are supported by MRC (MR/S019553/1, MR/R02622X/1, MR/V036939/1, MR/W020610/1), NIHR Imperial Biomedical Research Centre (BRC):ITMAT, Cystic Fibrosis Trust SRC (2019SRC015), NIHR EME Fast Track (NIHR134607), NIHR Long Covid (COV-LT2-0027), Innovate Uk (SBRI 10008614) and Horizon 2020 Marie Skłodowska-Curie Innovative Training Network (ITN) European Training Network (No 860325). ÁM is supported by MRC (MR/W020610/1), NIHR EME Fast Track (NIHR134607), Rosetrees Trust, The John Black Charitable Foundation, and Medical College of St Bartholomew's Hospital Trust. The COVIDsortium is supported by funding donated by individuals, charitable Trusts, and corporations including Goldman Sachs, Kenneth C Griffin, The Guy Foundation, GW Pharmaceuticals, Kusuma Trust, and Jagclif Charitable Trust, and enabled by Barts Charity with support from UCLH Charity. Wider support is acknowledged on the COVIDsortium website. Institutional support from Barts Health NHS Trust and Royal Free NHS Foundation Trust facilitated study processes, in partnership with University College London and Queen Mary University of London. MKM is supported by UKRI/NIHR UK-CIC, Wellcome Trust Investigator Award (214191/Z/18/Z) and CRUK Immunology grant (26603). JCM, CM and TAT are directly and indirectly supported by the University College London Hospitals (UCLH) and Barts NIHR Biomedical Research Centres and through the British Heart Foundation (BHF) Accelerator Award (AA/18/6/34223). TT is funded by a BHF Intermediate Research Fellowship (FS/19/35/34374). MN is supported by the Wellcome Trust (207511/Z/17/Z) and by NIHR Biomedical Research Funding to UCL and UCLH. The funders had no role in study design, data collection, data analysis, data interpretation, or writing of the report. **Author contributions:** R.J.B conceptualized and designed the study reported. R.J.B and D.M.A designed and supervised the T cell and B cell experiments. Á.M. designed and supervised the nAb experiments. T.B. and A.Se supervised S1 IgG and N IgG/IgM studies. D.MS., KM.L., F.P., and D.K.B. performed and analyzed T cell experiments. C.J.R. developed, performed and analyzed T cell, and memory B cell experiments. J.M.G. and C.P developed, performed and analyzed nAb experiments. A.D.O. performed and A.Se analyzed RBD and N antibody assays. T.B., C.M., Á.M., T.T., J.C.M., and M.N conceptualized and established the COVIDsortium HCW cohort. R.J.B, T.T., J.C.M., and C.M designed the vaccine sub-study cohort. G.J., K.M., M.F., A.S., C.M., T.A.T., and J.C.M. recruited HCW and collected samples. B.K. and T.C-M. helped with confirmation of sequence of viral isolates. C.J.R., D.MS., KM.L., F.P., S.L., and D.K.B. and COVIDsortium Investigators processed HCW samples. C.J.R., J.M.G., C.P., Á.M., D.M.A., and R.J.B. analyzed the data. C.J.R., J.M.G., C.P., C.M., T.T., J.C.M., M.K.M., A.Se., T.B., M.N., Á.M., D.M.A., and R.J.B. interpreted the data. R.J.B. and D.M.A wrote the manuscript with input from all the authors. All the authors reviewed and edited the manuscript and figures. **Competing interests:** R.J.B. and D.M.A. are members of the Global T cell Expert Consortium and have consulted for Oxford Immunotec outside the submitted work. **Data and materials availability:** All data needed to evaluate the conclusions in the paper are present in the paper or the supplementary materials. The SARS-CoV-2 Wuhan Hu-1 Human 2019-nCoV, B.1.351, P.1 and B.1.617.2 isolates were obtained under material agreements with European Virus Archive Global (EVAg), France. The SARS-CoV-2 B.1.1.7 isolate was obtained under a material agreement with the National Institute for Biological Standards and Control (NIBSC), UK. This work is licensed under a Creative Commons Attribution 4.0 International (CC BY 4.0) license, which permits unrestricted use, distribution, and reproduction in any medium, provided the original work is properly cited. To view a copy of this license, visit https://creativecommons.org/licenses/by/4.0/. This license does not apply to figures/photos/artwork or other content included in the article that is credited to a third party; obtain authorization from the rights holder before using such material.

## SUPPLEMENTARY MATERIALS

science.org/doi/10.1126/science.abm0811
Materials and Methods
Figs. S1 to S11
Tables S1 to S13
References (*42*, *43*)
Data Table S14
MDAR Reproducibility Checklist

25 August 2021; accepted 25 November 2021
Published online 2 December 2021
10.1126/science.abm0811

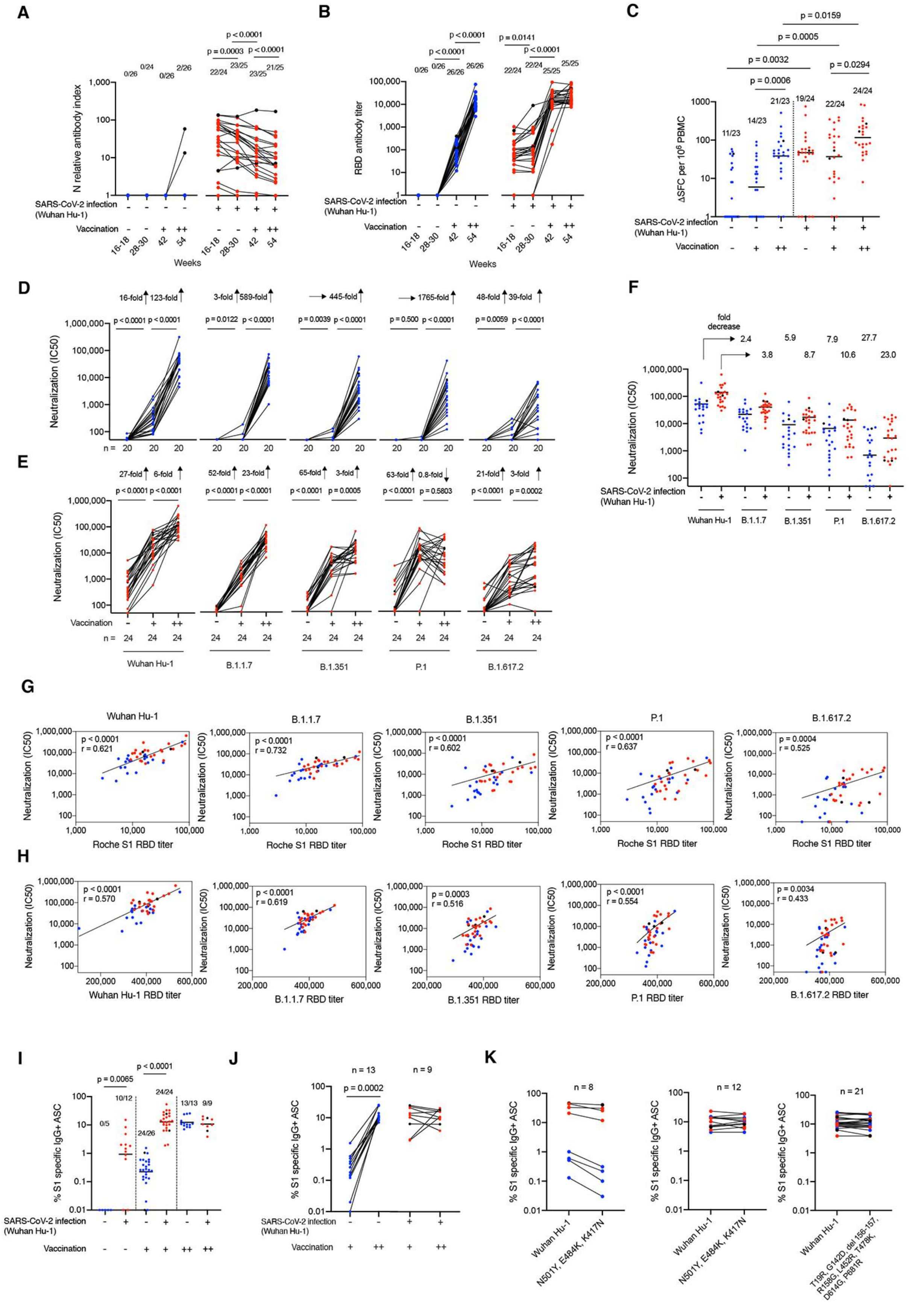
A
B
C
D
E
F
G
H
I
J
K
N relative antibody index
RBD antibody titer
ΔSFC per 10^6 PBMC
SARS-CoV-2 infection (Wuhan Hu-1)
Vaccination
Weeks
Neutralization (IC50)
fold decrease
Wuhan Hu-1
B.1.1.7
B.1.351
P.1
B.1.617.2
Roche S1 RBD titer
Wuhan Hu-1 RBD titer
B.1.1.7 RBD titer
B.1.351 RBD titer
P.1 RBD titer
B.1.617.2 RBD titer
% S1 specific IgG+ ASC
N501Y, E484K, K417N
T19R, G142D, del 156-157, R158G, L452R, T478K, D614G, P681R

**Fig. 1. T and B cell immunity to infection with Wuhan Hu-1 SARS-CoV-2 and following one and two doses of BNT162b2 vaccine in infection naïve and prior infected HCW.** (**A**) N Ab and (**B**) S1 RBD serum Ab titers measured by ECLIA in HCW with (red, *n* = 25) and without (blue, *n* = 26) lab-confirmed SARS-CoV-2 infection at 16-18 weeks after the start of the first UK epidemic wave and 2-3 weeks following first (+, 42 weeks) and second (++, 54 weeks) dose BNT162b2 vaccination. In all figures HCW without lab confirmed infection are shown in blue and HCW with lab confirmed infection are shown in red. HCW with new infection or re-infection after 30 weeks are shown in black. (**C**) Magnitude of T cell responses to spike MEP pool in HCW with (red, *n* = 24) and without (blue, *n* = 23) lab-confirmed SARS-CoV-2 infection 16-18 weeks after the start of the first UK epidemic wave and 2-3 weeks following first (+, 42 weeks) and second (++, 54 weeks) dose vaccination. Neutralizing Ab titer (IC50) against authentic Wuhan Hu-1 live virus and B.1.1.7, B.1.351, P.1 and B.1.617.2 VOCs plotted longitudinally for (**D**) SARS-CoV-2 infection naïve (blue, *n* = 20) and (**E**) prior Wuhan Hu-1 infected HCW (red, *n* = 24) unvaccinated and following the first and second dose BNT162b2 vaccination. (**F**) IC50 nAb against Wuhan Hu-1 and VOCs following two dose BNT162b2 vaccination in prior infected (red, *n* = 24) and infection naïve HCW (blue, *n* = 20). (**G**) Correlation between Roche S1 RBD Ab titer and nAb (IC50) against SARS-CoV-2 authentic Wuhan Hu-1 live virus and B.1.1.7, B.1.351, P.1 and B.1.617.2 VOC. (**H**) Correlation between Wuhan Hu-1, VOC RBD and corresponding live virus in two dose BNT162b2 vaccinated prior infected (red, *n* = 24) and SARS-CoV-2 infection naïve (blue, *n* = 20) HCW (**I**) Percentage of S1 specific IgG+ antibody secreting cells (ASC) in SARS-CoV-2 infection naïve and prior infected HCW at 16-18 weeks and following first and second dose BNT162b2 vaccination. (**J**) Percentage of S1 specific IgG+ ASC plotted pairwise after first and second dose vaccination for HCW with (red, *n* = 9) and without (blue, *n* = 13) prior SARS-CoV-2 infection. (**K**) left hand panel, percentage of IgG+ ASC specific for Wuhan Hu-1 S1 protein or spike protein containing the mutations N501Y, E484K and K417N in HCW with (red) and without (blue) prior SARS-CoV-2 infection following first dose vaccination (*n* = 4 per group). Middle and right-hand panels, percentage of IgG+ ASC specific for Wuhan Hu-1 S1 or S1 spike protein containing N501Y, E484K, K417N (B.1.351 VOC) mutations or S1 spike protein containing T19R, G142D, del 156-157, R158G, L452R, T478K, D614G, P681R (B.1.617.2 VOC) mutations after second dose BNT162b2 vaccination (middle panel, *n* = 6 per group, right-hand panel infection naïve are indicated in blue, *n* = 13 and prior Wuhan Hu-1 infected are indicated in red, *n* = 9). In (A), (B), (D), (E), and (H), Wilcoxon matched-pairs signed rank test was used; (C) and (I), Mann-Whitney *U* test; (G), Spearman's rank correlation. New infection (in the infection naïve group, *n* = 2) and re-infection (in the prior infected group, *n* = 2) data points shown in black were excluded from statistical analysis. ASC, antibody secreting cells; HCW, health care worker; N, nucleoprotein; RBD, receptor binding domain; S1, spike subunit 1; SFC, spot forming cells; VOC, variant of concern.

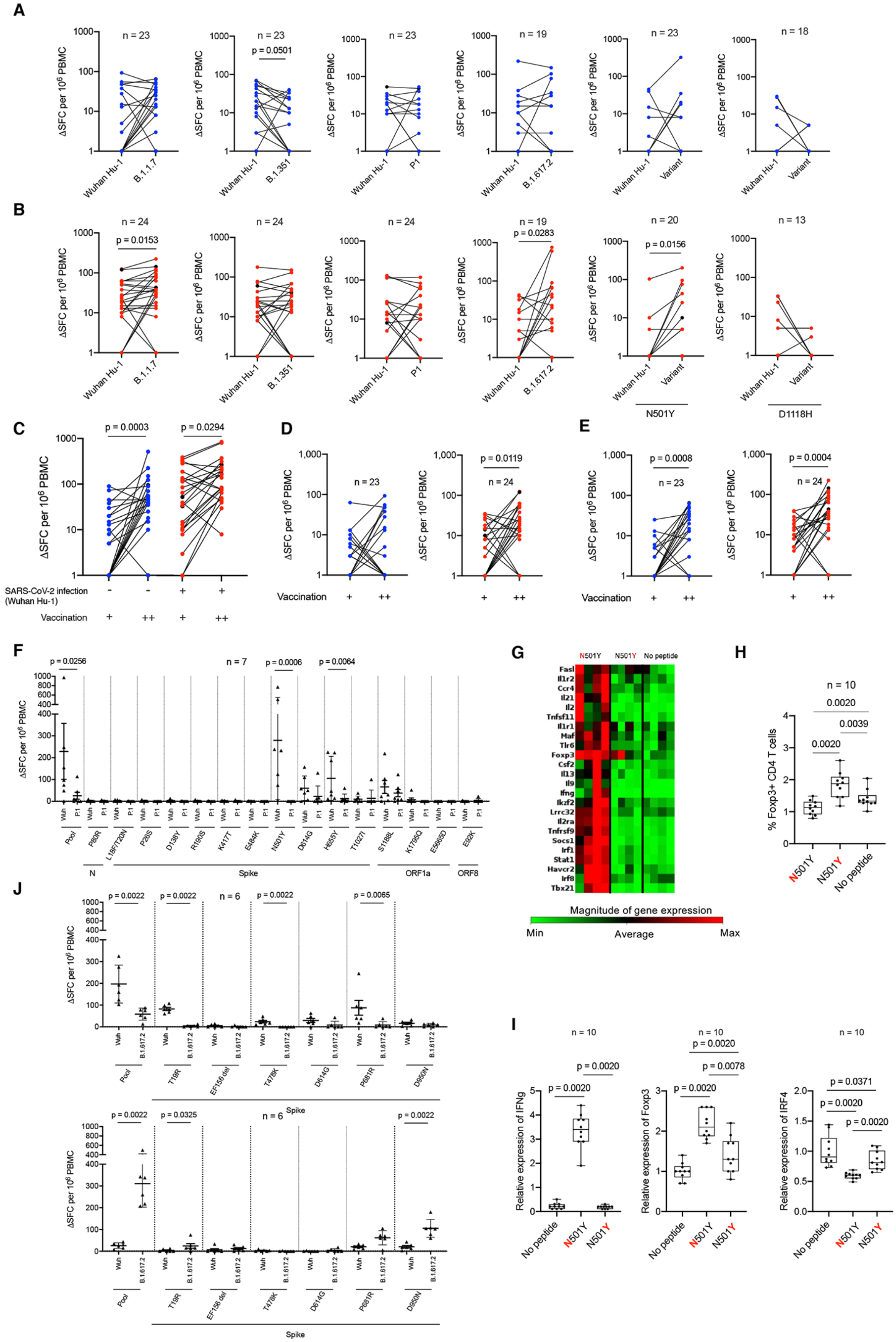
A
n = 23
n = 19
n = 18
p = 0.0501
ΔSFC per 10⁶ PBMC
Wuhan Hu-1
B.1.1.7
B.1.351
P1
B.1.617.2
Variant
B
n = 24
n = 20
n = 13
p = 0.0153
p = 0.0283
p = 0.0156
N501Y
D1118H
C
p = 0.0003
p = 0.0294
SARS-CoV-2 infection (Wuhan Hu-1)
Vaccination
D
n = 23
n = 24
p = 0.0119
E
p = 0.0008
p = 0.0004
F
p = 0.0256
n = 7
p = 0.0006
p = 0.0064
N
Spike
ORF1a
ORF8
G
N501Y
N501Y
No peptide
Magnitude of gene expression
Min
Average
Max
H
n = 10
0.0020
0.0039
0.0020
% Foxp3+ CD4 T cells
No peptide
J
p = 0.0022
p = 0.0065
n = 6
p = 0.0325
Spike
I
n = 10
p = 0.0020
p = 0.0078
p = 0.0371
Relative expression of IFNg
Relative expression of Foxp3
Relative expression of IRF4

**Fig. 2. T cell responses to Wuhan Hu-1 and VOC peptide pools following one and two dose BNT162b2 vaccination in infection naïve and prior infected HCW.** Magnitude of T cell responses to B.1.1.7, B.1.351, P.1 and B.1.617.2 variant specific peptide pools and to the matched sequence peptide pools from Wuhan Hu-1 as well as to individual wild type and variant N501Y and D1118H peptides in (**A**) SARS-Cov-2 infection naïve (blue) HCW and (**B**) prior infected (red) HCW that had received two doses of BNT162b2 vaccine. In all figures HCW without lab confirmed infection are shown in blue, HCW with lab confirmed infection are shown in red and HCW with new infection or re-infection after 30 weeks are shown in black. Magnitude of T cell responses to (**C**) Spike MEP pool, (**D**) Wuhan Hu-1 and (**E**) B.1.1.7 variant peptide pools following one (+) and two (++) doses of BNT162b2 vaccine in infection naïve HCW (blue) and prior infected HCW (red). (**F**) Magnitude of T cell response to wild-type Wuhan Hu-1 and P.1 variant peptide pools and individual peptides in Wuhan Hu-1 peptide pool immunized HLA-DRB1*04:01 transgenic mice ($n = 7$). (**G**) Heat-map showing relative gene expression of T cell activation markers in DLN cells from Wuhan Hu-1 N501Y peptide primed DRB1*04:01 transgenic mice ($n = 4$) stimulated for 24h in vitro with 10μg/ml of wild type Wuhan Hu–1 or variant peptide. Genes shown in red are significantly different ($P < 0.05$) between control and Wuhan Hu-1 stimulated cells with a fold change greater than 1.5. (**H**) Percentage of Foxp3+ CD4 T cells by flow cytometry in DLN cells from Wuhan Hu-1 N501Y peptide primed DRB1*04:01 transgenic mice ($n = 10$) stimulated for 24hr in vitro with 10mg/ml of wild-type or variant peptide. (**I**) Relative gene expression of *ifng, foxp3* and *irf4* in DLN cells from Wuhan Hu-1 N501Y peptide primed DRB1*0401 transgenic mice ($n = 10$) stimulated for 24hr in vitro with 10μg/ml of Wuhan Hu-1 or variant peptide. (**J**) Magnitude of T cell response to wild-type Wuhan Hu-1 and B.1.617.2 variant peptide pools and individual peptides in Wuhan Hu-1 peptide pool immunized (top panel, $n = 6$) or B.1.617.2 peptide pool immunized (bottom panel, $n = 6$) HLA-DRB1*04:01 transgenic mice. In (A) to (E), (I), and (J), Wilcoxon matched-pairs signed rank test was used. New infection (in the infection naïve group, $n = 2$) and re-infection (in the prior infected group, $n = 2$) data points shown in black were excluded from statistical analysis. In (F) and (G), Mann-Whitney *U* test was used. DLN, draining lymph node; HCW, health care workers; SFC, spot forming cells; VOC, variant of concern.

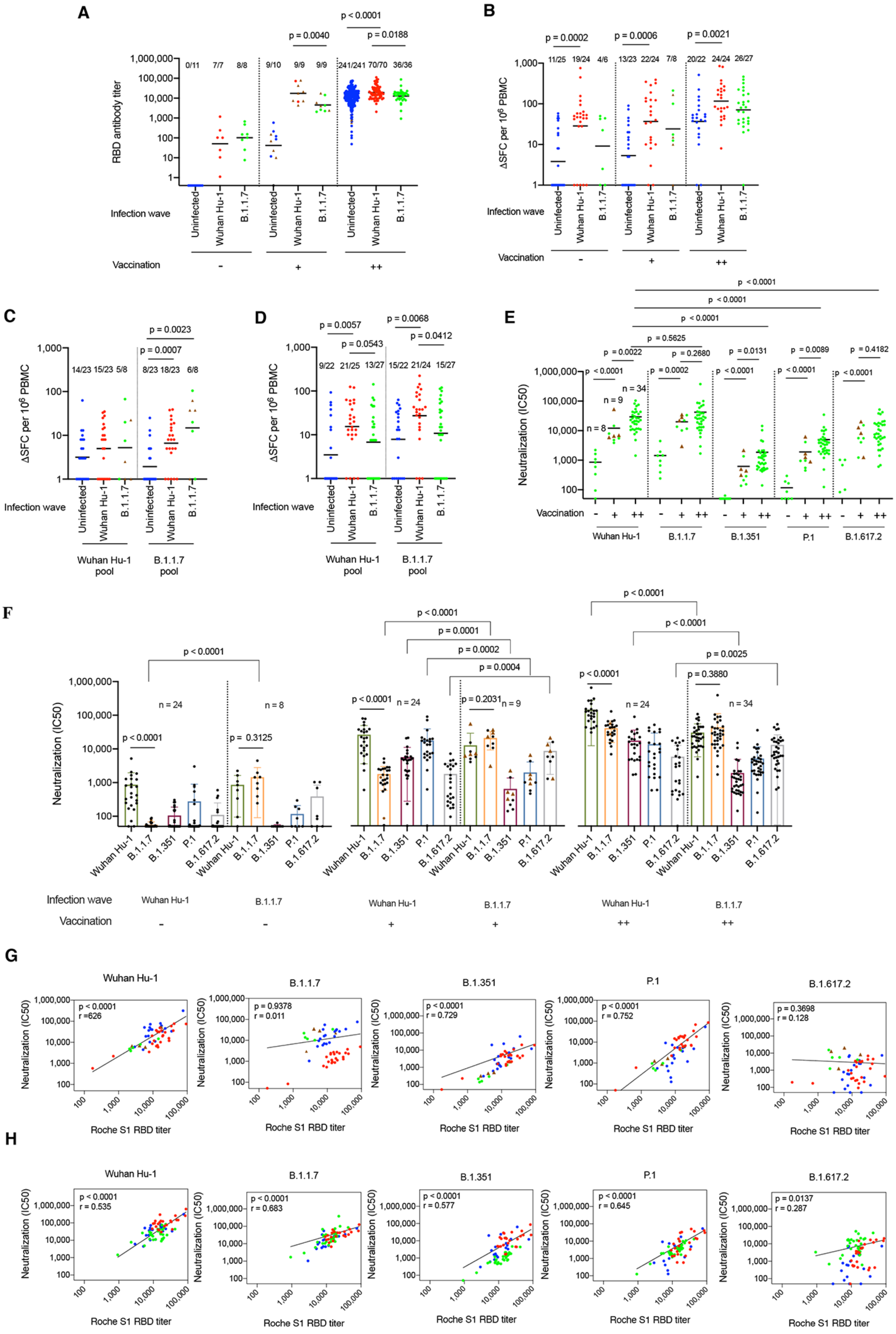
A
RBD antibody titer
Infection wave
Vaccination
Uninfected
Wuhan Hu-1
B.1.1.7
p < 0.0001
p = 0.0040
p = 0.0188
0/11 7/7 8/8 9/10 9/9 9/9 241/241 70/70 36/36
B
ΔSFC per 10^6 PBMC
p = 0.0002
p = 0.0006
p = 0.0021
11/25 19/24 4/6 13/23 22/24 7/8 20/22 24/24 26/27
C
p = 0.0023
p = 0.0007
14/23 15/23 5/8 8/23 18/23 6/8
Wuhan Hu-1 pool
B.1.1.7 pool
D
p = 0.0057
p = 0.0543
p = 0.0068
p = 0.0412
9/22 21/25 13/27 15/22 21/24 15/27
E
Neutralization (IC50)
p < 0.0001
p = 0.5625
p = 0.0022
p = 0.2680
p = 0.0002
p = 0.0131
p = 0.0089
p = 0.4182
n = 8
n = 9
n = 34
B.1.351
P.1
B.1.617.2
F
p < 0.0001
p = 0.3125
p = 0.0001
p = 0.0002
p = 0.0004
p = 0.2031
p = 0.0025
p = 0.3880
n = 24
n = 8
n = 9
n = 34
G
Roche S1 RBD titer
p < 0.0001 r = 626
p = 0.9378 r = 0.011
p < 0.0001 r = 0.729
p < 0.0001 r = 0.752
p = 0.3698 r = 0.128
H
p < 0.0001 r = 0.535
p < 0.0001 r = 0.683
p < 0.0001 r = 0.577
p < 0.0001 r = 0.645
p = 0.0137 r = 0.287

**Fig. 3. T cell and B cell immunity and neutralization hierarchy following heterologous exposure through infection with the B.1.1.7 VOC during the second UK wave and in the context of single and two dose vaccination.** (**A**) RBD Ab titers at 54-57 weeks after the start of study recruitment in March 2020 in HCW who were not infected with SARS-CoV-2 (blue, *n* = 256), infected during the first UK wave by the Wuhan Hu-1 strain (red, *n* = 86) or during the second UK wave by the B.1.1.7 (green, *n* = 53). (**B**) Magnitude of T cell response to Wuhan Hu-1 spike MEP peptide pool. Data are plotted according to whether individuals were unvaccinated (-) or had received 1 (+) or 2 (++) doses of BNT162b2 vaccine. Magnitude of T cell response to B.1.1.7 peptide pools (Wuhan Hu-1 or B.1.1.7 variant peptides) after (**C**) first dose or (**D**) second dose of vaccine. Neutralizing Ab titers (IC50) against authentic Wuhan Hu-1 live virus and B.1.1.7, B.1.351, P.1 or B.1.617.2 VOC in HCW infected with SARS-CoV-2 during B.1.1.7 wave (**E**) and during Wuhan Hu-1 first UK wave (**F**), plotted according to whether individuals were unvaccinated (-, Wuhan Hu-1 wave *n* = 24, B.1.1.7 wave *n* = 8)) or had received 1 (+, Wuhan Hu-1 wave *n* = 24, B.1.1.7 wave *n* = 9) or 2 (++, Wuhan Hu-1 wave *n* = 24, B.1.1.7 wave *n* = 34) doses of vaccine. Correlation between Roche S1 RBD Ab titer and nAb (IC50) against authentic Wuhan Hu-1 live virus and B.1.1.7, B.1.351, P.1 and B.1.617.2 VOC in one (**G**) and two (**H**) dose BNT162b2 vaccinated HCW prior infected by Wuhan Hu-1 (red, *n* = 23) or B.1.1.7 infected (green, one dose *n* = 9, two doses *n* = 31) and two dose vaccinated infection-naïve (blue, *n* = 19) HCW at 54-57 weeks after initial study recruitment. In all graphs, individuals who received the ChAdOx1 nCoV-19 vaccine are marked as brown triangles. In (A) to (E), Mann-Whitney *U* test was used; (F) Mann-Whitney *U* test or Wilcoxon matched-pairs signed rank test; (G) and (H), Spearman's rank correlation. HCW, Health care workers; RBD, receptor binding domain; S1, Spike subunit; SFC, spot forming cells; VOC, variant of concern.

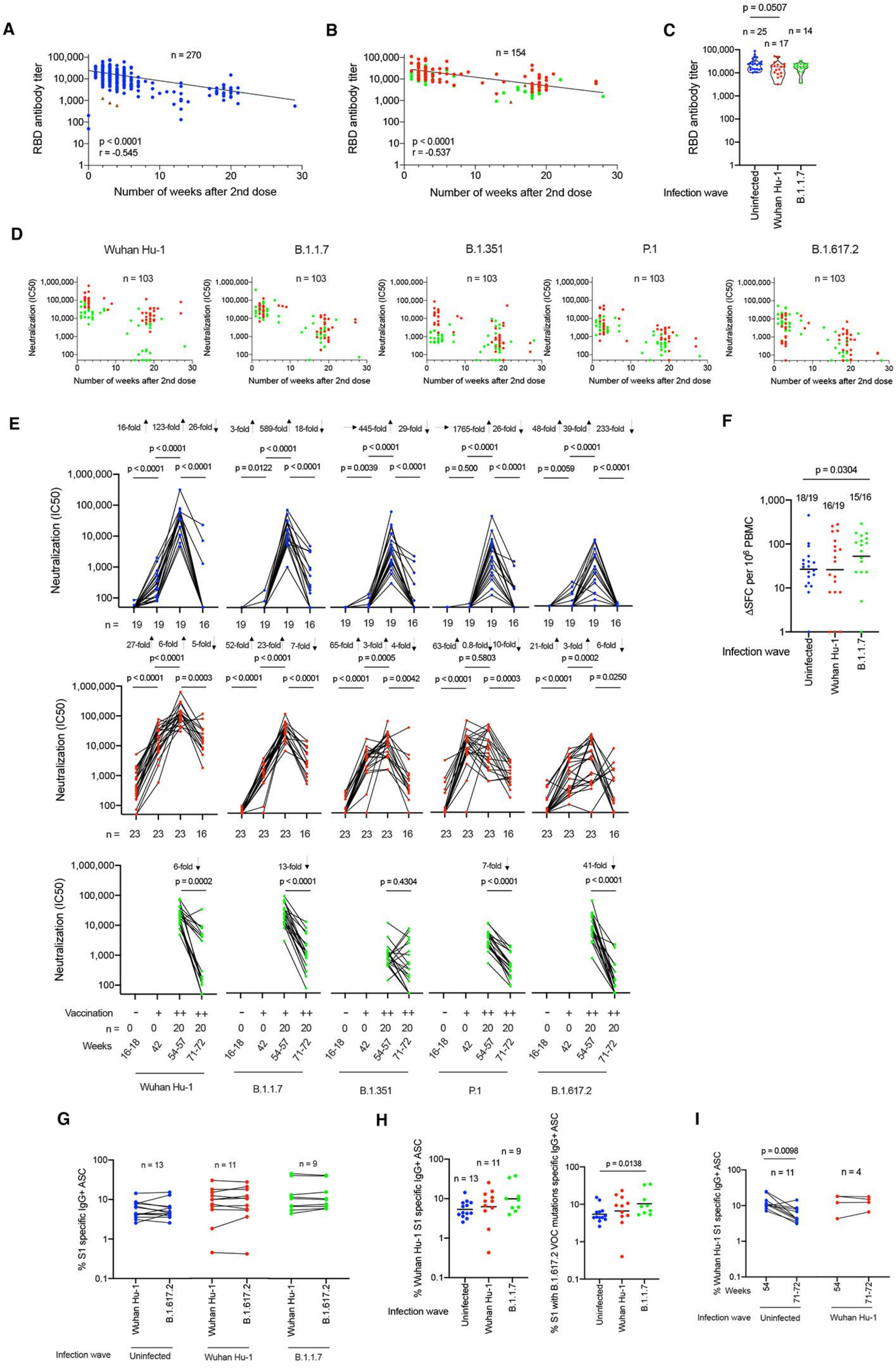
A
B
C
D
E
F
G
H
I
RBD antibody titer
Number of weeks after 2nd dose
n = 270
p < 0.0001
r = -0.545
n = 154
p < 0.0001
r = -0.537
p = 0.0507
n = 25
n = 17
n = 14
Infection wave
Uninfected
Wuhan Hu-1
B.1.1.7
Wuhan Hu-1
B.1.1.7
B.1.351
P.1
B.1.617.2
n = 103
Neutralization (IC50)
Vaccination
Weeks
p = 0.0304
18/19
16/19
15/16
ΔSFC per 10^6 PBMC
% S1 specific IgG+ ASC
n = 13
n = 11
n = 9
% Wuhan Hu-1 S1 specific IgG+ ASC
% S1 with B.1.617.2 VOC mutations specific IgG+ ASC
p = 0.0138
p = 0.0098
n = 4

**Fig. 4. Differential impact of heterologous exposure through B.1.1.7 infection on neutralization of other VOC and durability of the immune response.** (**A**) S1 RBD Ab titers in HCW with no previous SARS-CoV-2 infection (blue) and two dose vaccination (*n* = 270) (**B**) S1 RBD Ab titers in HCW with a history of SARS-CoV-2 infection (Wuhan Hu-1, red; B.1.17, green) and two dose vaccination (*n* = 154), plotted by the number of weeks that serum was sampled after the second vaccine dose. HCW that received the ChAdOx1 nCoV-19 vaccine are indicated by brown triangles. (**C**) S1 RBD Ab titers in infection naïve HCW (blue, *n* = 25) or those with a history of SARS-CoV-2 infection with the Wuhan Hu-1 strain (red, *n* = 17) or B.1.17 VOC (green, *n* = 14) and three dose vaccination, sampled between 10d and 7 weeks after the third vaccine dose. (**D**) Neutralizing Ab titers (IC50) against authentic Wuhan Hu-1 live virus and B.1.1.7, B.1.351, P.1 and B.1.617.2 VOC in HCW with a history of previous SARS-CoV-2 infection who had received two vaccine doses, plotted by the number of weeks that serum was sampled after the most recent vaccine dose. (**E**) Neutralizing Ab titer (IC50) against authentic Wuhan Hu-1 live virus and B.1.1.7, B.1.351, P.1 and B.1.617.2 VOCs plotted longitudinally 20d following the first (+) and 20 days following the second (++) dose and then 21 weeks following the second BNT162b2 vaccination. SARS-CoV-2 infection naïve (blue, upper panel), Wuhan Hu-1 prior infected (red, middle panel) and B.1.1.7 infected (green, lower panel). (**F**) Magnitude of T cell response to spike MEP peptide pool at 71-72 weeks after initial study recruitment in double vaccinated infection naïve HCW (blue, *n* = 19) and HCW with history of SARS-CoV-2 infection by Wuhan Hu-1 (red, *n* = 19) or B.1.1.7 (green, *n* = 16). (**G**) Percentage of IgG+ ASC specific for Wuhan Hu-1 S1 or S1 protein containing T19R, G142D, del 156-157, R158G, L452R, T478K, D614G, P681R (B.1.617.2 VOC) mutations at 71-72 weeks after initial study recruitment in double vaccinated infection naïve (blue, *n* = 13) HCW and those with a history of SARS-CoV-2 infection with the Wuhan Hu-1 strain (red, *n* = 11) and B.1.1.7 VOC (green, *n* = 9) 21 weeks after the second vaccine dose. (**H**) Percentage of IgG+ ASC specific for Wuhan Hu-1 S1 protein (left hand panel) or S1 protein containing T19R, G142D, del 156-157, R158G, L452R, T478K, D614G, P681R (B.1.617.2 VOC) mutations (right hand panel) at 71-72 weeks after initial study recruitment in double vaccinated infection naïve HCW (blue, *n* = 13), prior Wuhan Hu-1 infected (red, *n* = 11) and B.1.1.7 infected (green, *n* = 9) HCW (**I**) Percentage of IgG+ ASC specific for Wuhan Hu-1 S1 protein in double vaccinated HCW with (red, *n* = 4) or without (blue, *n* = 11) a history of Wuhan Hu-1 infection at 54 and 71-72 weeks after initial study recruitment. In (A) and (B), Spearman's rank correlation was used; (C), (F), and (H), Mann-Whitney *U* test; (E) and (I), Wilcoxon matched-pairs signed rank test. HCW, health care workers; RBD, receptor binding domain. ASC, antibody secreting cells; HCW, health care worker; RBD, receptor binding domain; S1, spike subunit 1; SFC, spot forming cells; VOC, variant of concern.
