# Supplementary material for: Heterologous infection and vaccination shapes immunity against SARS-CoV-2 variants
Source: Science. 2021 Dec 2;375(6577):183–92. doi: 10.1126/science.abm0811 (PMC10186585; doi:10.1126/science.abm0811)
Supplement: Supplementary file 2 — Materials and Methods Figs. S1 to S11 Tables S1 to S13 Members of the COVIDsortium Immune Correlates Network and COVIDsortium Investigators References (42, 43) [file science.abm0811_sm.pdf]

Science
AAAS

Supplementary Materials for

Heterologous infection and vaccination shapes immunity against SARS-CoV-2 variants

Catherine J. Reynolds *et al.*

Corresponding author: Rosemary J. Boyton, r.boyton@imperial.ac.uk

Science **375**, 183 (2022)
DOI: 10.1126/science.abm0811

The PDF file includes:

Materials and Methods
Figs. S1 to S11
Tables S1 to S13
Members of the COVIDsortium Immune Correlates Network and COVIDsortium Investigators
References

Other Supplementary Material for this manuscript includes the following:

Data Table S14
MDAR Reproducibility Checklist

MATERIALS AND METHODS

Ethics statement

The COVIDsortium HCW bioresource is registered on ClinicalTrials.gov (NCT04318314) and approved by the UK National Research Ethics Service (20/SC/0149). Subjects gave written, informed consent and the study conformed to the Helsinki Declaration principles. Mouse experiments were performed under U.K Home Office Legislation and the Animals (Scientific Procedures) Act 1986 and Project Licence P809B6A94 granted for this work.

COVIDsortium Healthcare Worker Participants

Recruitment of 731 adult HCW into the COVIDsortium bioresource along with details of the COVIDsortium sampling protocol has been described (*5-10*) (Fig. S1). A cross-sectional case controlled sub-study of 136 HCW recruited 16-18 weeks after March 2020 UK lockdown reported immunity to SARS-CoV-2 natural infection during the UK 1st wave (Wuhan Hu-1) (*6*). SARS-CoV-2 infection was determined by baseline and weekly nasal RNA stabilizing swabs and Roche cobas® SARS-CoV-2 reverse transcriptase polymerase chain reaction (RT-PCR) test and baseline and weekly Ab testing for S1 using the IgG EUROIMMUN enzyme-linked immunosorbent assay (ELISA) and nucleocapsid using the ROCHE Elecsys electrochemiluminescence immunoassay (ECLIA). Ab ratios >1.1 were deemed positive for the EUROIMMUN SARS-CoV-2 ELISA and >1 was considered test positive for the ROCHE Elecsys anti-SARS-CoV-2 ECLIA, as evaluated by UK Health Security Agency (UKHSA), Porton Down, UK. The majority of HCW infections during the first UK wave occurred by March 2020 when UK cases were a mix of the D and G sequences with respect to the D614G

mutation. Analysis of those infected in the first wave (Wuhan Hu-1) showed no evidence of two populations suggestive of a divergent neutralization response (*6, 9*). We concluded from this and other published studies that any differential immunological impact of the D614G mutation would be likely marginal *(42, 43)*. A cross-sectional, case-controlled vaccine sub-study cohort of 51 HCW at a mean timepoint of 22d (±2d SD) after administration of the first dose of BNT162b2 vaccines reported immunity to vaccination in individuals with and without a history of prior SARS-CoV-2 infection during the 1st UK (Wuhan Hu-1) wave (*9*). The vaccine sub-study recruited HCW previously enrolled in the 16-18 week sub-study. It included 25 HCW (mean age 44y, 60% male) with previous lab-defined SARS-CoV-2 infection and 26 HCW (mean age 41y, 54% male) with no laboratory evidence of SARS-CoV-2 infection throughout the initial 16-week longitudinal follow up. The current sub-study includes longitudinal follow up of the previously published vaccine sub-study cohort (n=51) at a median timepoint of 20 days (7, IQR) after administration of the second dose of (Fig. S1, Table S1) BNT162b2 at 54 weeks f/u and an additional 358 HCW recruited at 55-57 weeks follow-up, 53 of whom were infected by the B.1.1.7 VOC during the second UK wave (Fig. S1, Table S5, 6). At 71-72 weeks follow-up 80 two dose vaccinated HCW were re-recruited that were either SARS-CoV-2 infection naïve (n=27) or had been infected by Wuhan Hu-1 during the first wave (n=31) or B.1.1.7 during the second UK wave (n=22) (Fig. S1, Table S7). At 83-84 weeks follow-up, 74 previously two dose vaccinated HCW were re-recruited that were either SARS-CoV-2 infection naïve (n=35) or had been infected by Wuhan Hu-1 during the first wave (n=20) or B.1.1.7 during the second UK wave (n=19) (Table S9). Of these, 67 (91%) had received a third dose of BNT162b2 at a median timepoint of 18 days (12, IQR) previously.

Isolation of PBMC

Peripheral blood mononuclear cells (PBMC) were isolated from heparinized blood using Histopaque®-1077 Hybri-Max™ (Sigma-Aldrich) density gradient centrifugation in SepMate™ tubes (Stemcell) as previously described (*6, 9*). Isolated PBMCs were cryopreserved in fetal calf serum containing 10% DMSO and stored in liquid nitrogen.

Isolation of Serum

Whole blood samples in SST vacutainers (VACUETTE® #455092) were clotted at room temperature, 1h, then centrifuged, 10 minutes at 800xg. Serum was aliquoted and stored at -80^{0}C for SARS-CoV-2 Ab detection.

Anti-nucleocapsid and anti-spike antibody detection Ab testing was conducted at UK Health Security Agency (UKHSA), Porton Down, UK using the Roche cobas®e801 analyser. Anti-nucleocapsid Ab were detected using the qualitative Roche Elecsys® anti-SARS-CoV-2 electrochemiluminescence immune analyzer (ECLIA) nucleocapsid assay (Roche ACOV2, Product code: 09203079190) while anti-RBD Ab were detected using the quantitative Roche Elecsys® anti-SARS-CoV-2 ECLIA spike assay (Roche ACOV2S, Product code: 09289275190). Assays were performed and calibrated as recommended by the manufacturer. Anti-N results were expressed as a cutoff index (COI) value based on the electrochemiluminescence signal of a two-point calibration, with results COI $\geq$ 1.0 classified as positive. Anti-spike results were expressed as units per ml (U/ml) similarly based on a two-point calibration and a reagent specific master curve, with a quantitative range of 0.4 to 225,000 U/ml. Samples with a value of $\geq$0.8 U/ml were interpreted as positive for spike antibody, and

samples exceeding >250 U/ml were diluted to achieve sample results within the quantitatve range. (0.4 to 250 U/ml.

Recombinant proteins

Wild-type SARS-CoV-2 S1 spike protein (Z03485-1), E484K, K417N, N501Y spike protein (Z03531-100) and T19R, G142D, del 156-157, R158G, L452R, T478K, D614G, P681R spike protein (Z03612-1) were purchased from GenScript USA Inc. SARS-CoV-2 Spike Glycoprotein (S1) RBD, SARS-CoV-2 (N501Y Mutant), (501Y.V2: K417N, E484K, N501Y), (B.1.1.28: K417T, E484K, N501Y) and (B.1.617.2: L452R, T478K) Spike Glycoprotein (S1) RBDs derived from Wuhan Hu-1, B.1.1.7, B.1.351, P.1 and B.1.617,2 VOC respectively were purchased from the Native Antigen Company, UK.

Peptides

Spike mapped epitope pool (MEP) comprises a pool of eighteen 12-20mer peptide epitopes (*6, 9*). Wuhan Hu-1 and variant peptide pools were designed to contain peptides from the Wuhan Hu-1 sequence, B.1.1.7, B.1.351, P.1 and B.1.617.2 sequence, whereby the Wuhan Hu-1 peptides and their respective variant peptides containing the amino acid mutations and deletions of the B1.1.7, B.1.351, P.1 and B.1.617.2 variant SARS-CoV-2 strains (Table S2). Note that the B.1.617.2 peptide pool was additionally supplemented with a peptide carrying the E484Q mutation since it had been described in the earlier B.1.617.1 mutant. The B.1.617.2 mutation, L452R, was not included in T cell analysis as the relevant peptide had been omitted from the synthesis and transposed for the peptide L425R:APGQTGKIADYNYKRPDDFT.

These contain predicted HLAII binding motifs as determined by NetMHCIIpan4.0 (*9, 38, 39),* Table S3, S4). Peptides were synthesised by GL Biochem Shanghai Ltd (China).

T cell assay by IFNγ-ELISpot

IFNγ-ELISpots were conducted as previously described (*6, 9*). Precoated ELISpot plates (Mabtech 3420-2APT) were washed x4 with PBS, blocked for 1h (room temperature) with supplemented RPMI1640 (GibcoBRL) (10% heat inactivated FCS; 1% 100xpenicillin, streptomycin and L-Glutamine solutions (GibcoBRL)). 200,000 PBMC were seeded/well and stimulated 18-22h at 37^0C with SARS-CoV-2 recombinant protein (10μg/ml) or peptide (10μg/ml/peptide). Negative and positive plate controls were medium or anti-CD3 (Mabtech mAb CD3-2). After stimulation, culture supernatants were stored for Luminex cytokine analysis. ELISpot plates were developed with 1μg/ml biotinylated anti-human IFNγ detection Ab conjugated to alk-phosphatase (7-B6-1-ALP, Mabtech), diluted in PBS/0.5% FCS, adding 50μl/well for 2h at room temperature followed by 50μl/well BCIP/NBT-plus phosphatase substrate (Mabtech), 5 minutes (room temperature). Plates were washed and dried before analysis on an AID classic ELISpot plate reader (Autoimmun Diagnostika GMBH, Germany). ELISpot data was analyzed in Microsoft Excel. The average of two culture media alone wells was subtracted from all protein/peptide stimulated wells and any response that was lower in magnitude than 2 standard deviations of the sample specific control wells was not considered a stimulation-specific response. Results were expressed as difference in (delta) spot forming cells (SFC)/10^6 PBMC between negative control and protein/peptide stimulation conditions. Results were excluded if negative control wells showed >100SFC/10^6 PBMC (n=4) or cell viability was low with <1000 SFC/10^6 PBMC in anti-CD3 positive control wells (n=16). Results were plotted using Prism 9.0 for Mac OS (GraphPad).

B cell ELISpots

Prior to B cell ELISpot assays PBMCs were cultured for 5d (37^{0}C/5% CO_2) in 24-well plates, 500,000 cells/well containing 1μg/ml TLR7/8 agonist R848 plus 10ng/ml recombinant human IL-2 (Mabtech Human Memory B-cell Stimpack 3660-1). After 4d PBMC stimulation ELISpot PVDF plates (Millipore MSIPS4W10) were coated with PBS, purified anti-human IgG MT91/145 (10μg/ml, Mabtech 3850-3-250), SARS-CoV-2 S1 spike (10μg/ml), E484K, K417N, N501Y spike (10μg/ml) or T19R, G142D, del 156-157, R158G, L452R, T478K, D614G, P681R spike (10μg/ml), incubating with antigen at 4^{0}C overnight. Plates were washed x5 times and blocked for 1h with RPMI1640 (supplemented with 10% heat inactivated FCS, 1% 100x penicillin, streptomycin and L-Glutamine solutions (GibcoBRL)). Prestimulated PBMCs were washed x2 before seeding at 15,000-3,000 cells/well for anti-human IgG coated wells and 300,000-15,000 cells/well for SARS-CoV-2 spike coated wells. Assays were in duplicate, including duplicate control wells coated with PBS-only and seeded with 300,000 PBMC. Plates were incubated, 37^{0}C for 18-20h. For ELISpot development, plates were washed 5x times with PBS/0.05% Tween 20 (PBST) before incubation with 100μl biotinylated anti-human IgG MT78/145 (Mabtech 3850-6-250), in PBS/0.5% FCS, 2h, room temperature. Plates were washed x5 in PBST and incubated with 100μl/well 1:1000 Streptavidin-ALP (Mabtech 3310-10-1000), in PBS/0.5% FCS for 1h, room temperature. Plates were then washed x5 with PBST, once with dH_20 and spots developed by adding 100μl/well BCIP/NBT substrate (Mabtech). Reactions were stopped by washing and dried before analyzing on an AID classic ELISpot plate reader (Autoimmun Diagnostika GMBH, Germany). Analysis of ELISpot data was performed in Microsoft Excel. Spots counted for each well were adjusted for cell numbers seeded and the average of PBS only coated wells subtracted from antigen coated wells. Number of SARS-CoV-2 S antigen specific Ab secreting cells (ASC) was expressed as % of the total number of IgG ASC.

Variant of concern RBD ELISAs

Nunc 96-well immune ELISA plates were coated with 1μg/ml of Wuhan Hu-1 or VOC RBD recombinant proteins in carbonate buffer (Sigma Aldrich) for 2 hours at 37^{0}C before washing with PBS (0.05% Tween) (PBST) and blocking at 37^{0}C for 1 hour with PBS containing 1% Bovine Serum Albumin (BSA). Plates were washed in PBST again before application of 50 μl of diluted sera to each well. All serum dilutions were run in duplicate and a four-point dilution series was run for each sample. Following overnight incubation at 4^{0}C, plates were washed with PBST and wells incubated with 1:1000 dilution of Biotin Mouse Anti-human IgG (BD Pharmingen, 555785) at room temperature for 1 hour. Plates were washed again before application of 1:200 dilution of Streptavidin Horseradish Peroxidase (HRP) (Bio-techne, DY998) for 30 minutes followed by a final wash and then assay development using 3,3', 5,5;-tetramethylbenzidine (TMB) substrate (Sigma Aldrich, T0440). Colour development was stopped after 5 minutes by the addition of 0.18M H_2SO_4 and OD450nm values for each well measured using a FLUOstar® Omega Plate Reader. Analysis of ELISA data was performed in Prism 9.0 for Mac OS (GraphPad). ELISA units were calculated as the serum dilution required to given an OD450nm reading of 0,3.

Wuhan Hu-1 SARS-CoV-2, B.1.1.7, B.1.351 and P.1 variant titration

SARS-CoV-2 isolate stocks (including Wuhan Hu-1, B.1.1.7, B.1.351, P.1 and B.1.617.2) used in experiments (Table S11) were prepared and titrated as previously described (*6, 9*).

Wuhan Hu-1 SARS-CoV-2, B.1.1.7, B.1.351, P.1, and B.1.617.2 VOC microneutralization assays

SARS-CoV-2 microneutralization assays were carried out as described previously (*6, 9*). VeroE6 cells were seeded in 96-well plates 24h prior to infection. Duplicate titrations of heat-inactivated participant sera were incubated with $3x10^4$ FFU SARS-CoV-2 virus (TCID100) at 37°C, 1h. Serum/virus preparations were added to cells and incubated for 72h. Surviving cells were fixed in formaldehyde and stained with 0.1% (wt/vol) crystal violet solution (crystal violet was resolubilized in 1% (wt/vol) sodium dodecyl sulfate solution). Absorbance readings were taken at 570nm using a CLARIOStar Plate Reader (BMG Labtech). Negative controls of pooled pre-pandemic sera (collected before 2008), and pooled serum from neutralization positive SARS-CoV-2 convalescent individuals were spaced across the plates. Absorbance for each well was standardized against technical positive (virus control) and negative (cells only) controls on each plate to determine percentage neutralization values. IC50s were determined from neutralization curves. Representative neutralization curves for Wuhan Hu-1, B.1.1.7, B.1.351, P.1 and B.1617.2 variant SARS-CoV-2 authentic virus are shown in Fig S8. All authentic SARS-CoV-2 propagation and microneutralization assays were performed in a containment level 3 facility.

In silico epitope prediction

In silico predictions of HLA-DRB1 peptide-binding were performed using NetMHCIIpan-4.0 (*38, 39*) based on peptide length of 15 amino acids. HLA core binding sequences containing individual mutations were selected if within a peptide defined as a strong or weak binder by

the NetMHCIIpan-4.0 default parameters of rank score <1% (threshold for strong binder) and rank score <5% (threshold for weak binder).

HLA-DRB1*0401 transgenic T cell assays

Studies using HLAII transgenics carrying DRB1*0401 in the context of a homozygous knockout for murine H2-Aβ have been previously described (*40, 41*). Mice (female, 14 weeks) were immunized in one hind footpad with a pool containing 10μg each peptide sequence in Hunters Titermax Gold adjuvant (Sigma Aldrich). Popliteal lymph nodes were collected at d10 and prepared as single cell suspensions. IFNγ ELISpot assays were performed in triplicate in HL1 serum-free medium (Lonza) (supplemented with 1% 100x L-glutamine and 0.5% 100x penicillin/streptomycin solutions (GibcoBRL)). PVDF ELISpot plates (Merck Millipore MSIPN4550) were coated with anti-mouse IFNγ capture Ab (Diaclone Murine IFN gamma ELISpot Set, 862.031.020) overnight before seeding 200,000 lymph node cells/well and stimulating (72h, 37^{0}C with 5% CO_2) with peptide pools or individual SARS-CoV-2 wild-type or variant peptides (10μg/ml/peptide). Internal plate controls were culture media alone and staphylococcal enterotoxin B (SEB). Assays were developed using biotinylated anti-mouse IFNγ followed by streptavidin-alkaline phosphatase conjugate and BCIP/NTB substrate (Diaclone) before washing in tap water, drying and analyzing using an AID classic ELISpot plate reader (Autoimmun Diagnostika GMBH, Germany). Analysis of ELISpot data was performed in Microsoft Excel. The average from 3 culture media wells was subtracted from peptide-stimulated wells and any response that was <2SD of the sample specific control wells was not considered a peptide-specific response. Results were expressed as difference in (delta) SFC/10^6 PBMC between the negative control and peptide stimulation conditions. Results were plotted using Prism 9.0 for Mac OS (GraphPad).

For transcriptomic and flow cytometry analysis, lymph node cells were cultured with no peptide, or 10μg/ml wild-type or variant N501Y peptides. At 24h, cells were harvested and lysed for RNA extraction or stained for flow cytometry. RNA was extracted using an Agilent RNA microprep kit. For gene arrays, cDNA was prepared using an RT2 first strand kit (Qiagen) and qPCR for target genes performed using RT^2 profiler PCR array Mouse T Helper Cell Differentiation plates (Qiagen PAMM-503Z). Data were analysed and plotted using the Qiagen GeneGlobe data analysis tool and genes upregulated by peptide stimulation greater than 1.5 fold with a p value > 0.05 (by students t-test) compared to no peptide stimulation were identified. For other qPCR, cDNA was prepared using Superscript III (ThermoFisher) and gene expression assays run using Taqman primers (*ifng* assay Mm01168134_m1, *foxp3* assay Mm00475156_m1, *irf4* assay Mm00516431_m1) and Brilliant II QPCR Low ROX Master Mix (Agilent). For flow cytometry, cells were surface stained with anti-mouse CD3e Fluorescein isothiocyanate (FITC) (BD Pharmingen, clone 145-2C11), anti-mouse CD4 Allophycocyanin (APC)/Cyanine 7 (Biolegend, clone GK1.5) and anti-mouse CD8 AlexaFluor®647 (Biolegend, clone 53-6.7) for 30 minutes at 4^{0}C in PBS containing 10% FBS. Cells were then fixed and permeabilised (Fixation/Permeabilization Solution Kit, BD Biosciences) for 20 minutes at 4^{0}C before washing in BD Perm/Wash™ Buffer and staining for 30 minutes at 4^{0}C with anti-mouse Foxp3 AlexaFluor®700 (eBioscience, clone FJK-16S). Following two final washes with BD Perm/Wash™ Buffer flow cytometry data was acquired using an Attune NxT acoustic focusing cytometer (Life Technologies). Data were analysed using FlowJo version 10.2 for Mac.

Statistics and reproducibility

Data was assumed to have a non-Gaussian distribution. Wilcoxon matched-pairs signed rank test and a Mann-Whitney U-test were used for single, paired and unpaired comparisons. Non-parametric tests were used throughout. For multiple unpaired comparisons, Kruskal-Wallis one-way Anova with Dunn's correction was used. For correlations, Spearman's r test was used. p value <0.05 was considered significant. Prism 9.0 for Mac was used for analysis.

Fig. S1A. CONSORT flow diagram of UK COVIDsortium healthcare worker cohort and vaccine sub-studies. CONSORT flow diagram showing participant recruitment into UK parent longitudinal COVIDsortium London healthcare worker study, first and second dose vaccine, B.1.1.7 wave, 55-57 and 71-72 week follow-up cross-sectional sub-study cohorts. Participants were initially stratified by SARS-CoV-2 PCR and anti-S1 antibody laboratory tests and the presence or absence of self-reported case definition or non-case definition symptoms experienced during 16-week follow-up and in the 3 months prior to study initiation.

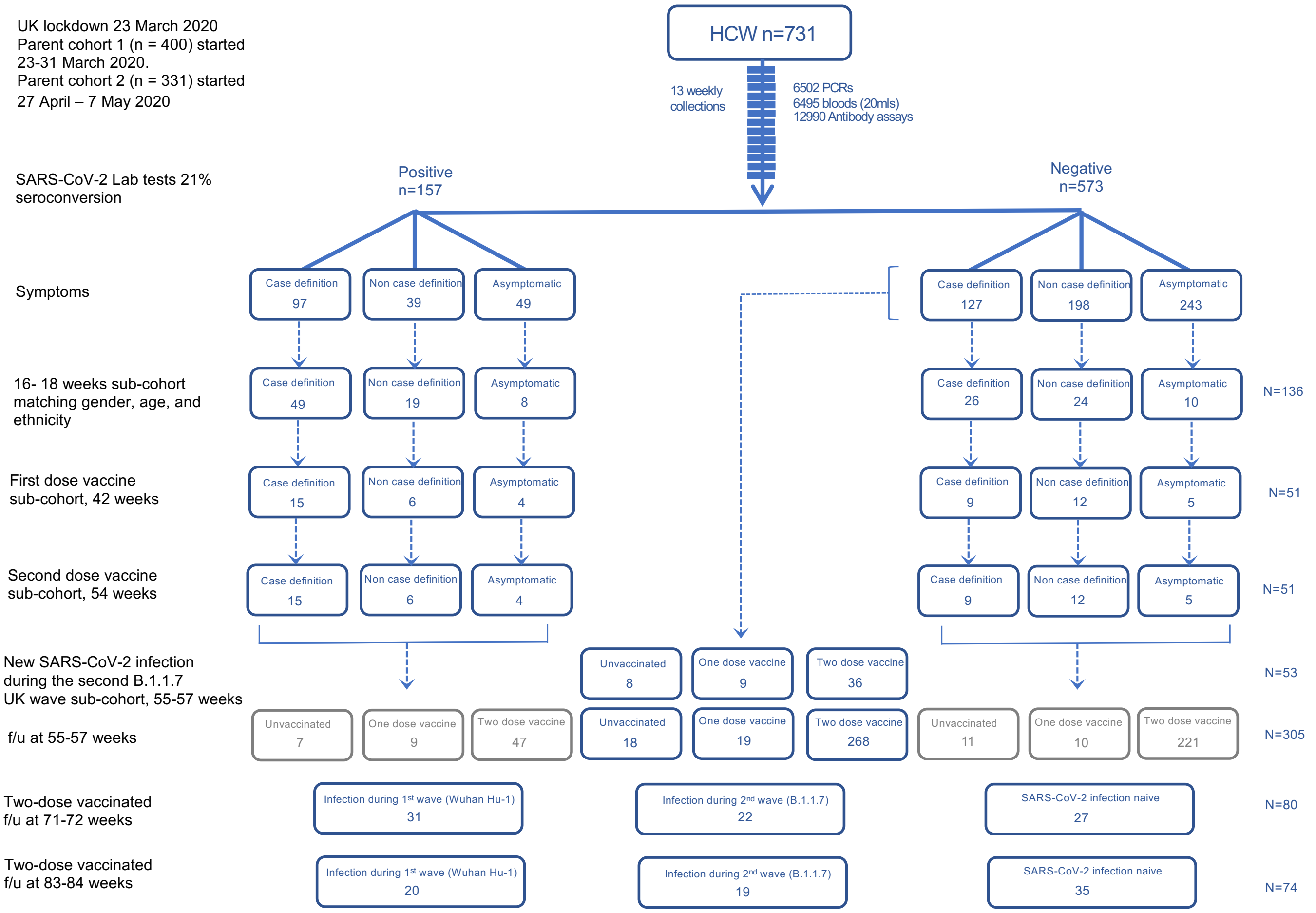

<u>Data analysis</u>

16-18 weeks sub-cohort:						
S1 RBD serology	15	6	3	9	12	5
N serology	15	6	3	9	12	5
T cell ELISpot	13	6	4	7	12	4
B cell ELISpot	7	2	3	1	3	0
nAb	14	6	4	8	10	2

First dose vaccine sub-cohort:						
S1 RBD serology	15	6	4	9	12	5
N serology	15	6	4	9	12	5
T cell ELISpot	13	6	4	7	12	4
B cell ELISpot	14	6	4	9	12	5
nAb	14	6	4	8	10	2

Second dose vaccine sub-cohort:						
S1 RBD serology	15	6	4	9	11	5
N serology	15	6	4	9	11	5
T cell ELISpot	14	6	4	9	11	3
B cell ELISpot	4	3	0	4	8	0
nAb	14	6	4	9	10	2

SARS-CoV-2 infection during the second B.1.1.7 UK wave			
S1 RBD serology	8	8	36
N serology	8	8	36
T cell ELISpot	6	8	26
B cell ELISpot	0	0	0
nAb	8	9	35

55-57 week f/u sub-cohort			
S1 RBD serology	18	19	268
N serology	18	19	268
T cell ELISpot	0	0	0
B cell ELISpot	0	0	0
nAb	0	0	0

71-72 week f/u sub-cohort			
S1 RBD serology	31	22	27
N serology	31	22	20
T cell ELISpot	19	20	27
B cell ELISpot	12	13	14
nAb	28	26	28

83-84 week f/u sub-cohort			
S1 RBD serology	20	19	35
N serology	20	19	35

Fig. S1B. Schematic diagram of UK COVIDsortium healthcare worker cohort and vaccine sub-study.

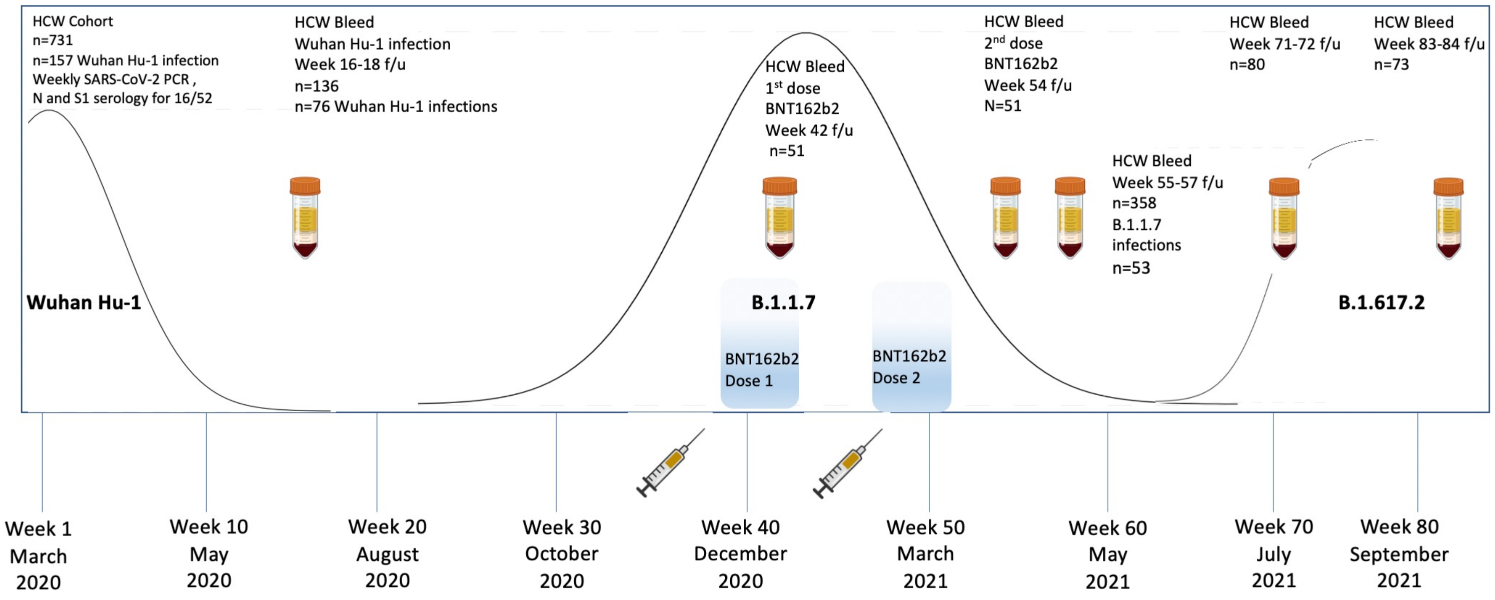

Fig. S2. Magnitude of T cell responses to nucleoprotein MEP pool in SARS-CoV-2 infection naïve (n = 23) and previously infected (n = 24) HCW who had received two doses of BNT162b2. A previously infection naïve HCW infected during the UK second wave just prior to or around the time of vaccination is shown in red. Mann-Whitney U test. HCW, health care workers.

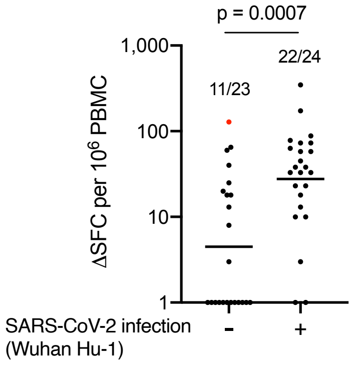

Fig. S3. Neutralizing antibody titer (IC50) against authentic Wuhan Hu-1 live virus and B.1.1.7, B.1.351, P.1 and B.1.617.2 VOC in HCW with (red, n = 24,) and without (blue, n = 20) lab-confirmed SARS-CoV-2 infection at 16-18 weeks after the first UK lockdown following one (+) and two (++) dose vaccination with BNT162b2 vaccine. HCW, health care workers; VOC, variant of concern.

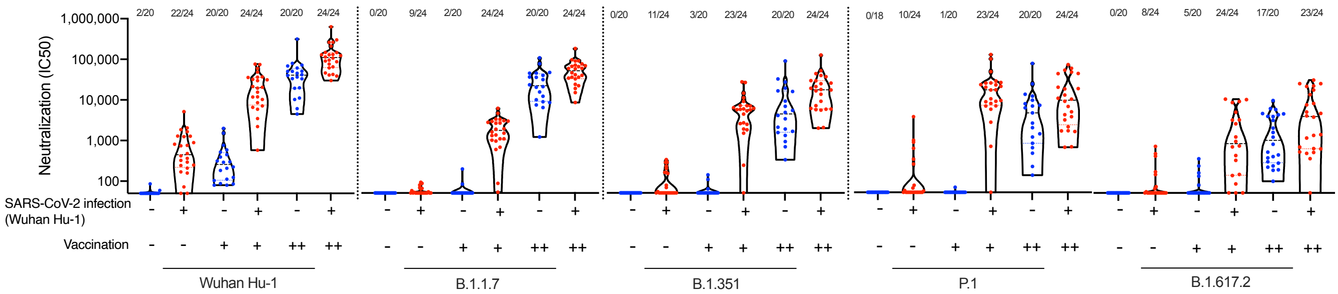

Fig. S4. Anti-RBD antibody titers to Wuhan Hu-1 and VOC proteins are correlated. Correlation between Wuhan Hu-1 RBD Ab titer and Ab titers against Roche S1, B.1.1.7, B.1.351, P.1 and B.1.617.2 RBDs in two dose BNT162b2 vaccinated prior infected (red, n = 23) and SARS-CoV-2 infection-naïve (blue, n = 24) HCW. HCW with new infection (n = 2) or re-infection (n = 2) after 30 weeks are shown in black. Spearman's rank correlation. HCW, health care worker; RBD, receptor binding domain; S1, spike subunit 1.

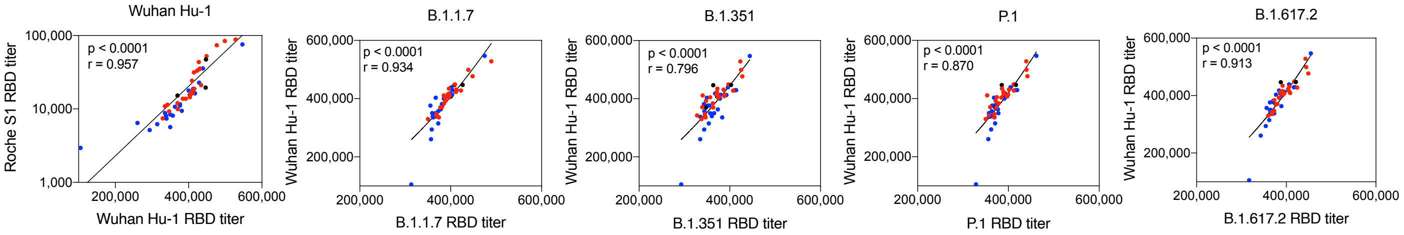

Fig. S5. Neutralizing antibody titer and percentage of spike protein specific memory B cells in two dose vaccinated HCW with and without a prior history of SARS-CoV-2 infection. (A) Correlation between percentage of IgG+ ASC specific for (**A**) Wuhan Hu-1 S1 spike protein, (**B**) S1 spike protein containing N501Y, E484K, K417N (B.1.351 VOC) mutations and (C) S1 spike protein containing T19R, G142D, del 156-157, R158G, L452R, T478K, D614G, P681R (B.1.617.2 VOC) mutations and neutralizing Ab titer (IC50) against authentic Wuhan Hu-1, B.1.1.7, B.1.351, P.1 and B.1.617.2 live virus in HCW with (red, n = 17 or n = 7) and without (blue, n = 17 or n = 12) prior SARS-CoV-2 infection. HCW with re-infection (n = 2) after 30 weeks are shown in black. Spearman's rank correlation. ASC, antibody secreting cells; ; VOC, variant of concern; HCW, health care worker; S1, spike subunit 1.

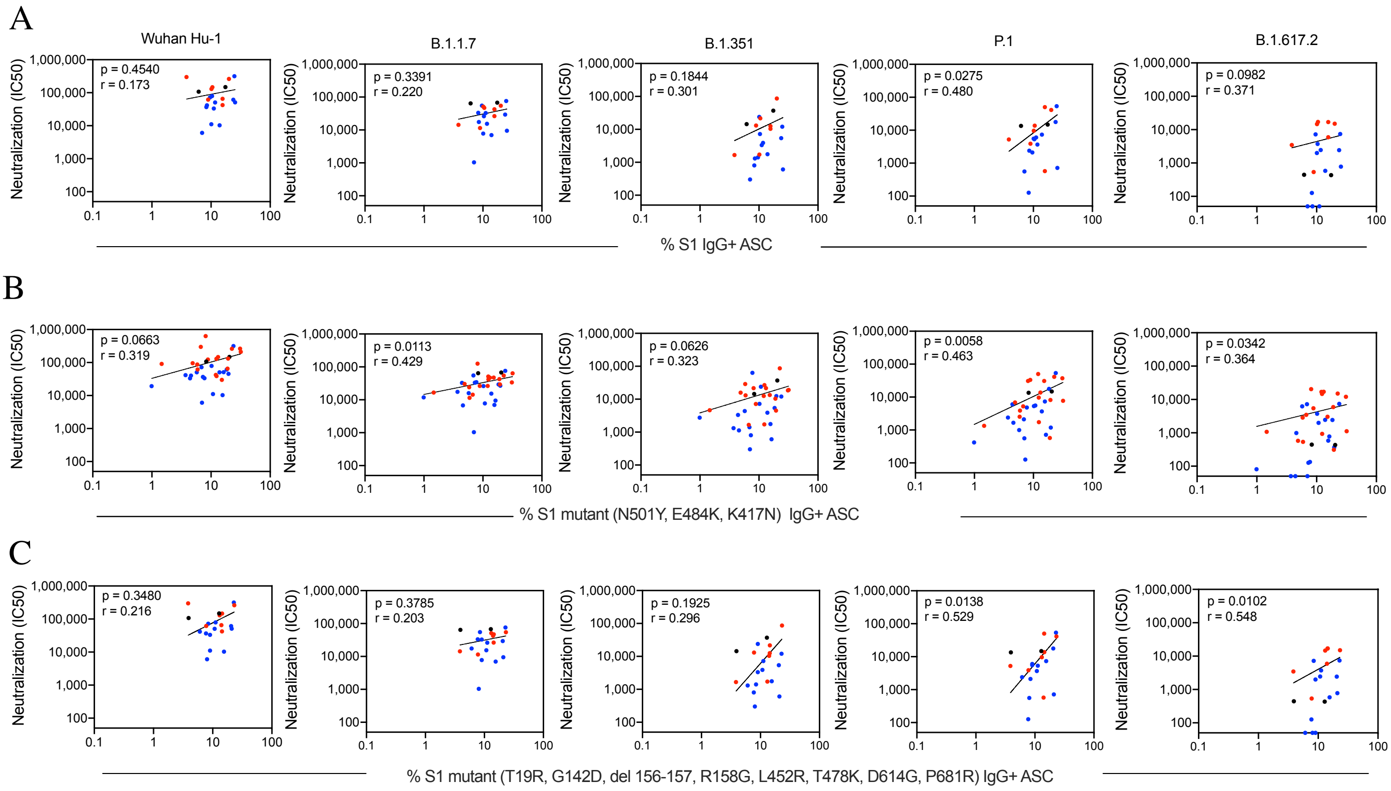

Fig. S6. Nucleocapsid (N) and Spike Ab following infection with B.1.1.7 VOC during the second UK wave and one or two doses of vaccine (A) N Ab and (**B**) S1 IgG antibody (Euroimmun) titer at 28-30 and 55-57 weeks after the start of study recruitment in March 2020 in HCW infected with SARS-CoV-2 B.1.1.7 during the second UK wave. Individuals who received the BNT162b2 vaccine are indicated using green circles and those who received the ChAdOx1 nCoV-19 vaccine are indicated by brown triangles. VOC, variant of concern; Ab, antibody; N, Nucleoprotein; S1, Spike subunit 1.

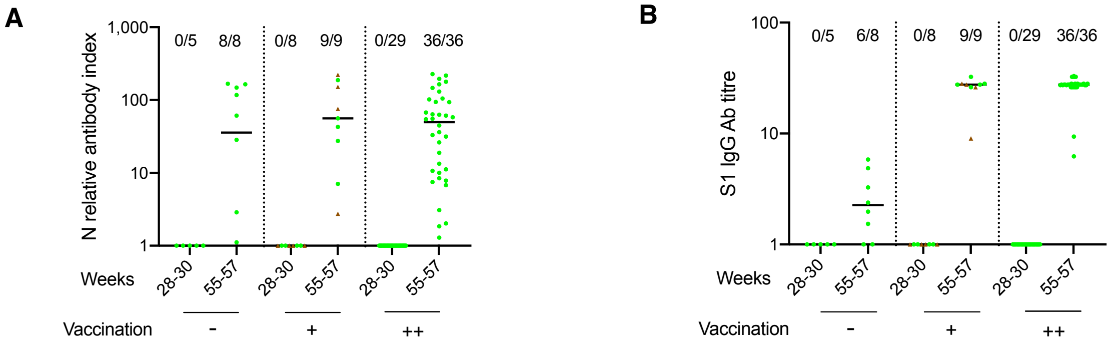

Fig. S7. Neutralization of Wuhan Hu-1 and B.1.1.7 VOC live virus by serum from HCW infected either during the first UK Wuhan Hu-1 infection wave or the second UK B.1.1.7 infection wave. HCW serum neutralizing antibody titers (IC50s) against Wuhan Hu-1 (grey bars) and B.1.1.7 (black bars) are shown for participants infected in either the Wuhan Hu-1 (left) or B.1.1.7 (right) infection waves. IC50s are shown for unvaccinated HCW and HCW vaccinated that had received one or two doses of COVID-19 vaccine. Kruskal-Wallis multiple comparison ANOVA with Dunn's correction. HCW, health care worker; VOC, variant of concern.

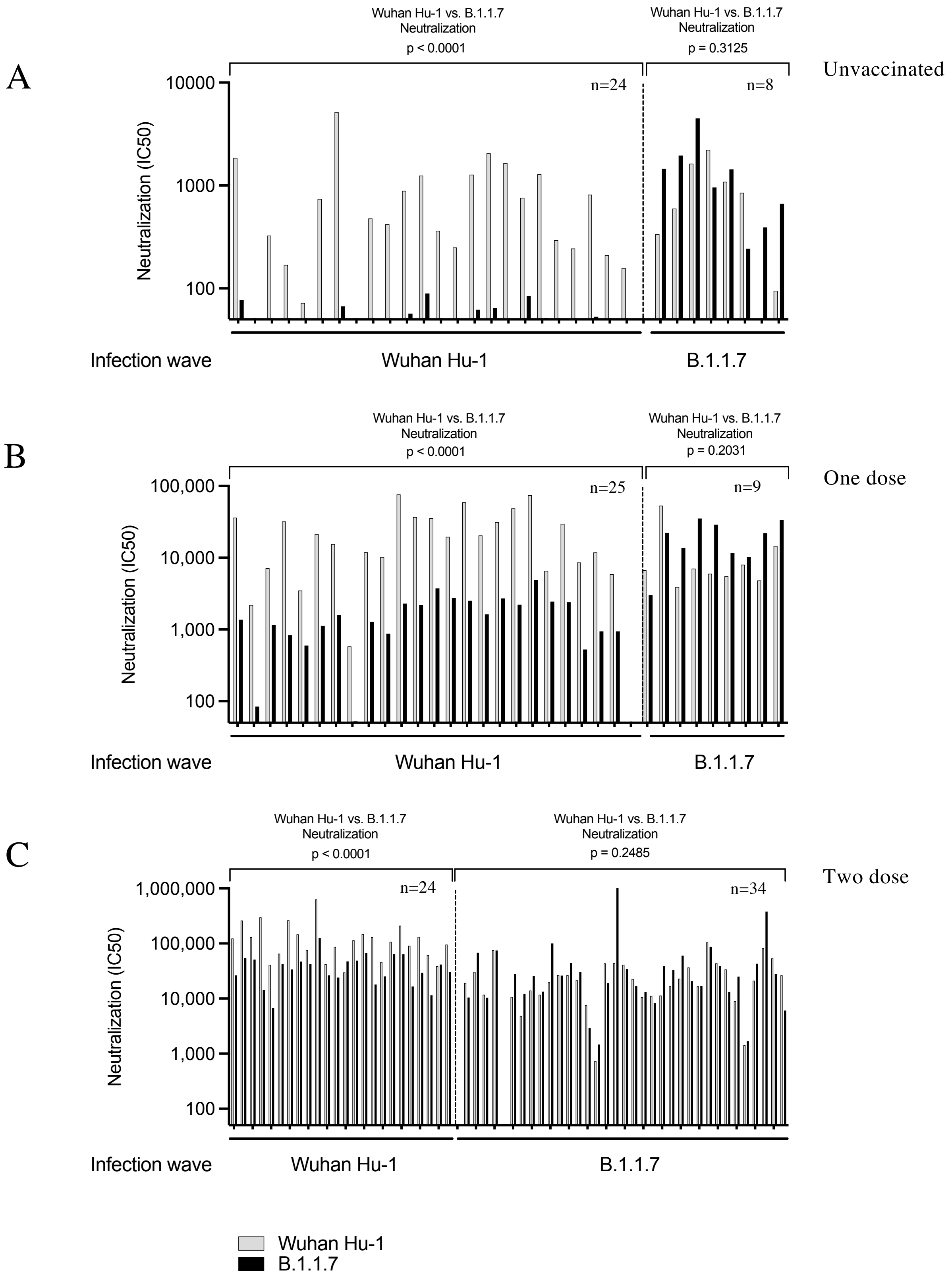

Fig. S8. Neutralization of Wuhan Hu-1, B.1.1.7, B.1.351, P.1 and B.1.617.2 variant SARS-CoV-2 authentic virus. HCW serum neutralisation (%) curves for Wuhan Hu-1 (green), B.1.1.7 (yellow), B.1.351 (plum) P.1 (blue) and B.1.617.2 (grey). Representative curves are shown for uninfected HCW, or HCW infected with SARS-CoV-2 either during the first UK Wuhan Hu-1 or second UK B.1.1.7 infection waves. Within each group, representative curves are shown for individuals who were unvaccinated or had received one or two doses of the BNT162b2 COVID-19 vaccine. Data points represent arithmetic means of technical replicates and error bars represent standard deviation. HCW, health care worker.

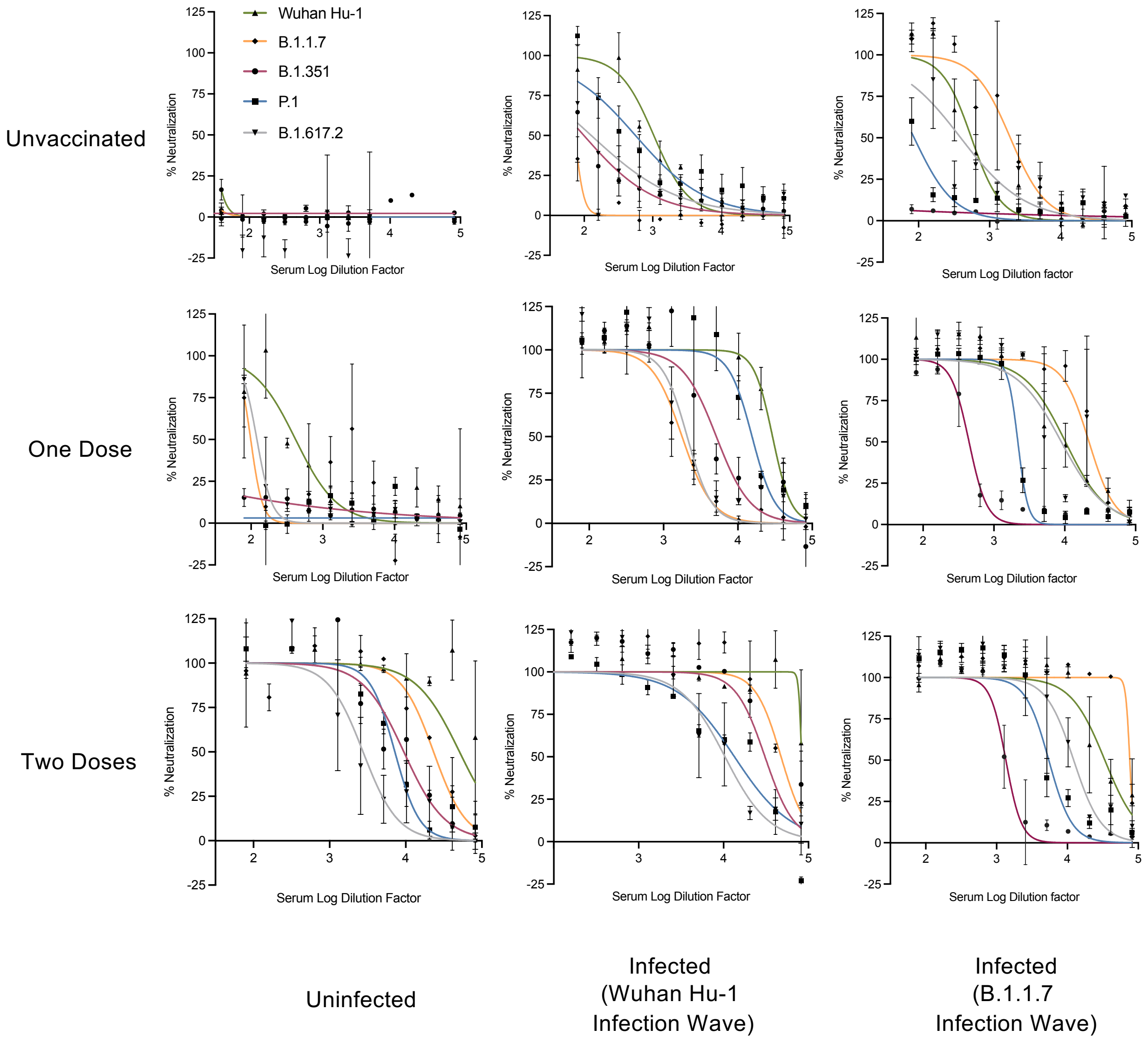

Fig. S9. Different VOC neutralization hierarchy in HCW infected with Wuhan Hu-1 or B.1.1.7 strains of SARS-CoV-2. nAb titers (IC50) against authentic Wuhan Hu-1 live virus and B.1.1.7, B.1.351, P1 or B.1.617.2 VOC in unvaccinated HCW with PCR positive confirmed infection by Wuhan Hu-1 (n = 5) or B.1.1.7 (n = 4) SARS-CoV-2 virus 15-17 weeks (Wuhan Hu-1 wave) or 13-19 weeks (B.1.1.7 wave) previously. Mann-Whitney U test. HCW, Health care workers; VOC, variant of concern.

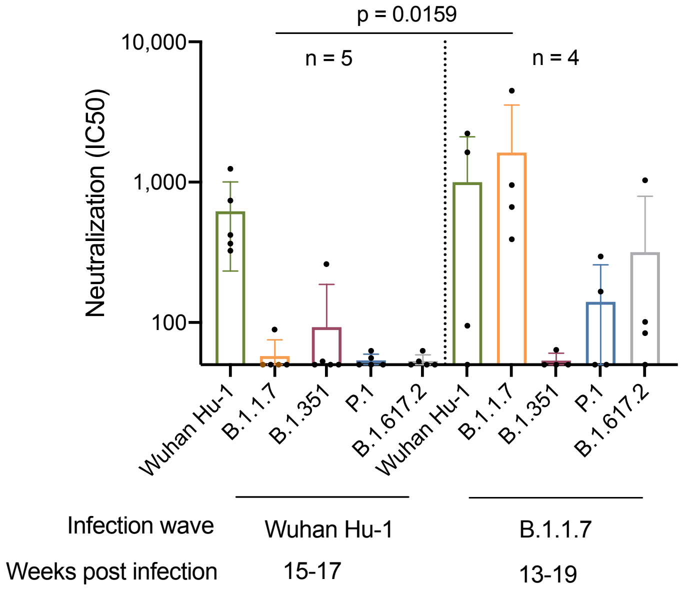

Fig. S10. Relationship between neutralization IC50 and anti-RBD Ab titer for Wuhan Hu-1 and SARS-CoV-2 VOC. Correlation between Wuhan Hu-1, B.1.1.7, B.1.351, P.1 or B.1.617.2 RBD Ab titer and nAb (IC50) against corresponding authentic SARS-CoV-2 live virus in (**A**) one and (**B**) two dose BNT162b2 vaccinated HCW prior infected by Wuhan Hu-1 (red , n = 23) or by B.1.1.7 (green, one dose n= 9, two doses n = 31) and two dose vaccinated SARS-CoV-2 infection naïve (blue, n = 19) HCW at 54-57 weeks after initial study recruitment. In all graphs, individuals who received the ChAdOx1 nCoV-19 vaccine are indicated by brown triangles. (**A, B**) Spearman's rank correlation. HCW, Health care workers; RBD, receptor binding domain; VOC, variant of concern.

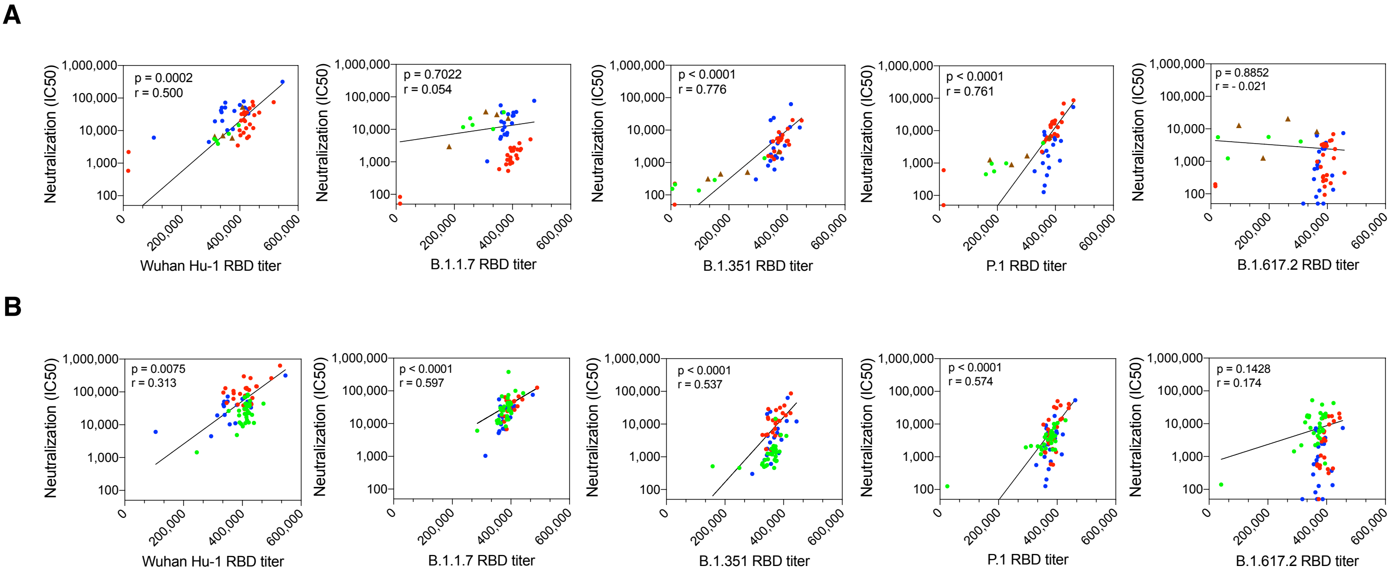

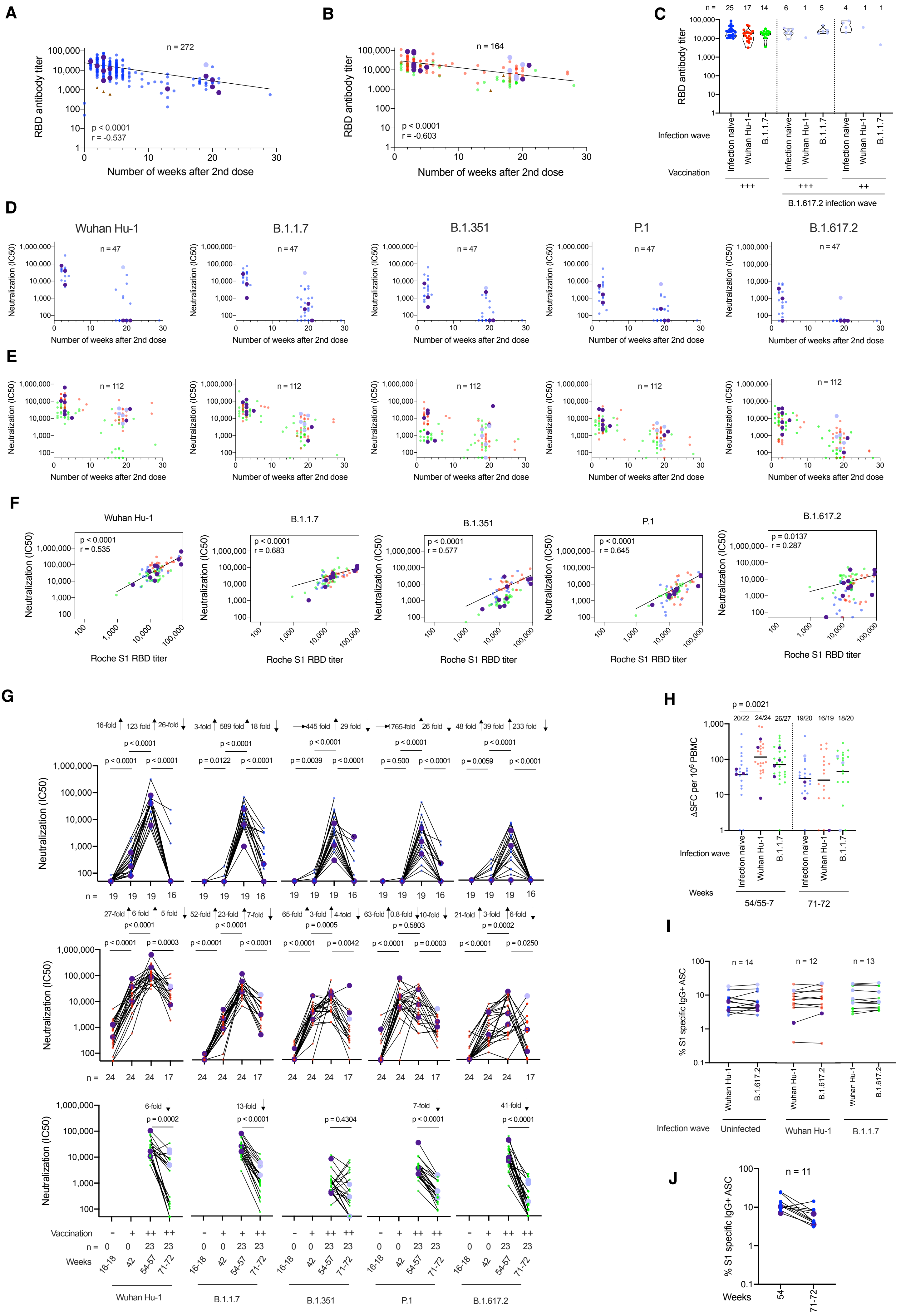

A
RBD antibody titer
n = 272
p < 0.0001
r = -0.537
Number of weeks after 2nd dose
B
n = 164
p < 0.0001
r = -0.603
C
Infection wave
Vaccination
Infection naive
Wuhan Hu-1
B.1.1.7
B.1.617.2 infection wave
D
Wuhan Hu-1
B.1.1.7
B.1.351
P.1
B.1.617.2
Neutralization (IC50)
n = 47
E
n = 112
F
Roche S1 RBD titer
p < 0.0001
r = 0.535
r = 0.683
r = 0.577
r = 0.645
p = 0.0137
r = 0.287
G
Vaccination
Weeks
H
ΔSFC per 10^6 PBMC
p = 0.0021
54/55-7
71-72
I
% S1 specific IgG+ ASC
Uninfected
n = 14
n = 12
n = 13
J
n = 11

Fig. S11. T and B cell immunity preceding B.1.617.2 breakthrough infection. (**A**) S1 RBD Ab titers in infection naïve HCW plotted against time in weeks since the second vaccine dose (blue, n=259) with those who went on to become infected (purple, n = 12) or had become infected (lilac, n = 1) during the UK B.1.617.2 wave at 71-72 and 83-84 weeks f/u respectively. (**B**) S1 RBD Ab titers in HCW with a history of SARS-CoV-2 infection (Wuhan Hu-1, red n = 96.; B.1.17, green n = 52) plotted against time in weeks since the second vaccine dose with those went on to become infected (purple, n = 11) or had become infected (lilac, n = 5) during the UK B.1.617.2 wave at 71-72 and 83-84 weeks f/u respectively. Data are plotted by the number of weeks that serum was sampled after the second vaccine dose. HCW that received the ChAdOx1 nCoV-19 vaccine are indicated by triangles. (**C**) S1 RBD Ab titers in three dose vaccinated infection naïve HCW (blue, n = 25) or with a history of prior SARS-CoV-2 infection with Wuhan Hu-1 (red, n = 17) or infection with B.1.17 VOC (green, n = 14). HCW with UK B.1.617.2 breakthrough infection (lilac) identified at 83-84 weeks f/u in HCW that were two (n = 6) and three (n = 12) dose vaccinated. Neutralizing Ab titers (IC50) against SARS-CoV-2 authentic Wuhan Hu-1 live virus and B.1.1.7, B.1.351, P.1 or B.1.617.2 VOC in two dose vaccinated infection naïve HCW (blue) (**D**) or with (**E**) a history of SARS-CoV-2 infection with Wuhan Hu-1, red; B.1.1.7, green; B.1.617.2, lilac plotted by the number of weeks after the second vaccine dose. HCW who went on to become infected during the B.1.617.2 wave are indicated in purple. (**F**) Correlation between Roche S1 RBD Ab titer and nAb (IC50) against SARS-CoV-2 authentic Wuhan Hu-1 live virus and B.1.1.7, B.1.351, P.1 and B.1.617.2 VOC in two dose BNT162b2 vaccinated infection naïve HCW (blue = 19), HCW prior infected with Wuhan Hu-1 (red , n = 23) or B.1.1.7 infected infected (green, n = 31) at 54-57 weeks after initial study recruitment. HCW who went on to become infected with B.1.617.2 are indicated in purple. (**G**) Neutralizing Ab titer (IC50) against authentic Wuhan Hu-1 live virus, B.1.1.7, B.1.351, P.1, and B.1.617.2 VOCs plotted longitudinally before vaccination and three weeks after one and two dose vaccination and twenty-one weeks after two dose vaccination. nAb IC50 data are shown for SARS-CoV-2 infection naïve (blue, upper panel) and HCW with a history of SARS-CoV-2 infection with Wuhan Hu-1 (red, middle panel) or B.1.1.7 (green, lower panel) are shown. HCW who went on to become (purple) or had been (lilac) infected with B.1.617.2 are indicated. Fold changes and statistics were calculated excluding HCW data plotted in lilac. (**H**) Magnitude of T cell response to spike MEP peptide pool at 54/55-57 (n = 73) and 71-72 (n = 59) weeks after initial study recruitment in double vaccinated HCW with and without a history of SARS-CoV-2 infection. (**I**) Percentage Percentage of IgG+ ASC specific for Wuhan Hu-1 S1 protein or S1 protein containing T19R, G142D, del 156-157, R158G, L452R, T478K, D614G, P681R (B.1.617.2 VOC) mutations at 71-72 weeks after initial study recruitment in double vaccinated HCW with or without a history of SARS-CoV-2 infection. (**J**) Percentage of IgG+ ASC specific for Wuhan Hu-1 S1 protein in double vaccinated infection naïve HCW (blue, n = 11) at 54 and 71-72 weeks after initial study recruitment. In all plots HCW who went on to become infected with B.1.617.2 are marked purple and all HCW who had been infected with B.1.617.2 are marked in lilac. (**A, B, F**) Spearman's rank correlation, (**G**) Wilcoxon matched-pairs signed rank test, (**H**) Mann-Whitney U test.. HCW, health care workers; RBD, receptor binding domain. ASC, antibody secreting cells; HCW, health care worker; RBD, receptor binding domain; S1, spike subunit 1; SFC, spot forming cells; VOC, variant of concern.

Table S1. First and second dose BNT162b2 COVID-19 vaccine study sub-cohort(s) with and without laboratory confirmed SARS-CoV-2 infection during the first UK wave

		Laboratory confirmed SARS-CoV-2 PCR$^+$ and/or Euroimmun S1 Ab$^+$ and/or Roche S1-RBD and/or N Ab$^+$ (first UK Wuhan Hu-1 wave)				SARS-CoV-2 PCR$^-$ and Euroimmun S1 Ab$^-$ Roche S1-RBD and N Ab- (first UK Wuhan Hu-1 wave)		
	Total	i) One or more COVID-19 case definition symptoms	ii) Non-case definition COVID-19 symptoms	iii) Asymptomatic	iv) All of i)-iii)	One or more COVID-19 case definition symptoms	Non-case definition COVID-19 symptoms	Asymptomatic
**HCW n (% of total)**	51 (100)	15 (29)	6 (12)	4 (8)	25 (49)	9 (18)	12 (23)	5 (10)
**Mean age (range)**	43 (24-62)	44 (27-58)	46 (26-58)	43 (33-62)	44 (26-62)	38 (25-58)	42 (27-62)	46 (24-60)
**Gender:**								
**Female n (%)**	22 (43)	8 (53)	1 (17)	1 (25)	10 (40)	3 (33)	6 (50)	3 (60)
**Ethnicity:**								
**White n (%)**	35 (69)	12 (80)	3 (50)	4 (100)	19 (76)	6 (67)	7 (58)	3 (60)
**Minority ethnic group (UK) n (%)**	16 (31)	3 (20)	3 (50)	0 (0)	6 (24)	3 (33)	5 (42)	2 (40)
**BNT162b2 COVID-19 vaccine n (%)**	51 (100)	15 (100)	6 (100)	4 (100)	25 (100)	9 (100)	12 (100)	5 (100)
**Number of days since second vaccine dose Median (IQR)**	20 (7)	21 (13)	21 (3)	18 (8)	21 (8)	17 (7)	20 (7)	15 (10)

Abbreviations: HCW, Healthcare worker; IQR, inter quartile range.

Table S2. Spike mapped epitope peptide (MEP) pool, Nucleoprotein(N) MEP pool, Wuhan Hu-1, B.1.1.7, B.1.351, P.1 and B.1.617.2 variant peptide pools and the matched sequence peptide pools from Wuhan Hu-1.

	**Position/mutation**	**Amino acid Sequence**
**Spike mapped epitope peptide pool (Spike MEP)**		
Spike	166-180	CTFEYVSQPFLMDLE
Spike	191-205	EFVFKNIDGYFKIYS
Spike	206-230	KHTPINLVRDLPQGF
Spike	211-225	NLVRDLPQGFSALEP
Spike	351-365	YAWNRKRISNCVADY
Spike	381-395	GVSPTKLNDLCFTNV
Spike	446-460	GGNYNYLYRLFRKSN
Spike	451-465	YLYRLFRKSNLKPFE
Spike	506-520	VVLSFELLHAPATVC
Spike	526-540	GPKKSTNLVKNKCVN
Spike	721-735	SVTTEILPVSMTKTS
Spike	746-760	STECSNLLLQYGSFC
Spike	751-765	NLLLQYGSFCTQLNR
Spike	801-815	NFSQILPDPSKPSKR
Spike	866-880	TDEMIAQYTSALLAG
Spike	1171-1185	GINASVVNIQKEIDR
Spike	1196-1210	LIDLQELGKYEQYI
Spike	1206-1220	YEQYIKWPWYIWLGF
**Nucleoprotein mapped epitope peptide pool (N MEP)**		
N	1-17	MSDNGPQNQRNAPRITF
N	8-25	NQRNAPRITFGGPSDSTG
N	82-95	DQIGYYRRATRRIR
N	101-113	MKDLSPRWYFYYL
N	104-121	LSPRWYFYYLGTGPEAGL
N	313-330	AFFGMSRIGMEVTPSGTW
N	321-338	GMEVTPSGTWLTYTGAIK
N	329-346	TWLTYTGAIKLDDKDPNF
N	344-361	PNFKDQVILLNKHIDAYK
N	352-369	LLNKHIDAYKTFPPTEPK

**Wuhan Hu-1 peptide pool (B.1.1.7)**		
N	S235F	LLLLDRLNQLESKMSGKGQQ
Spike	69-70 HV del	FSNVTWFHAIHVSGTNGTKR
Spike	144Y del	NDPFLGVYYHKNNKSWMESE
Spike	N501Y	YFPLQSYGFQPTNGVGYQPY
Spike	A570D	QFGRDIADTTDAVRDPQTLE
Spike	D614G	SNQVAVLYQDVNCTEVPVAI
Spike	P681H	CASYQTQTNSPRRARSVASQ
Spike	S982A	GAISSVLNDILSRLDKVEAE
Spike	D1118H	EPQIITTDNTFVSGNCDVVI
ORF1ab	I2230T	KSPNFSKLINIIIWFLLLSV
**B.1.1.7 variant peptide pool**		
N	S235F	LLLLDRLNQLESKMFGKGQQ
Spike	69-70 HV del	FSNVTWFHAISGTNGTKRFD
Spike	144Y del	NDPFLGVYHKNNKSWMESEF
Spike	N501Y	YFPLQSYGFQPTYGVGYQPY
Spike	A570D	QFGRDIDDTTDAVRDPQTLE
Spike	D614G	SNQVAVLYQGVNCTEVPVAI
Spike	P681H	CASYQTQTNSHRRARSVASQ
Spike	S982A	GAISSVLNDILARLDKVEAE
Spike	D1118H	EPQIITTHNTFVSGNCDVVI
ORF1ab	I2230T	KSPNFSKLINITIWFLLLSV
**Wuhan Hu-1 peptide pool (B.1.351)**		
N	T205I	STPGSSRGTSPARMAGNGGD
E	P71L	FYVYSRVKNLNSSRVPDLLV
Spike	L18F	LPLVSSQCVNLTTRTQLPPA
Spike	D80A	SGTNGTKRFDNPVLPFNDGV
Spike	D215G	KHTPINLVRDLPQGFSALEP
Spike	L242-244 del	NITRFQTLLALHRSYLTPGD
Spike	K417N	VRQIAPGQTGKIADYNYKLP
Spike	E484K	AGSTPCNGVEGFNCYFPLQS
Spike	N501Y	YFPLQSYGFQPTNGVGYQPY
Spike	D614G	SNQVAVLYQDVNCTEVPVAI
Spike	A701V	SIIAYTMSLGAENSVAYSNN
ORF1ab	T265I	EIKLAKKFDTFNGECPNFVF
ORF1ab	K1655N	RYMSALNHTKKWKYPQVNGL
ORF1ab	H2799Y	TPVHVMSKHTDFSSEIIGYK
ORF1ab	S2900L	AVGNICYTPSKLIEYTDFAT
ORF1ab	K3353R	HSMQNCVLKLKVDTANPKTP
ORF1ab	D4527Y	DLVYALRHFDEGNCDTLKEI
ORF1ab	T5912I	SDRDLYDKLQFTSLEIPRRN
ORF3a	Q57H	IVGVALLAVFQSASKIITLK
ORF3a	S171L	NSVTSSIVITSGDGTTSPIS
**B.1.351 variant peptide pool**		
N	T205I	STPGSSRGISPARMAGNGGD
E	P71L	FYVYSRVKNLNSSRVLDLLV
Spike	L18F	LPLVSSQCVNFTTRTQLPPA
Spike	D80A	SGTNGTKRFANPVLPFNDGV
Spike	D215G	KHTPINLVRGLPQGFSALEP
Spike	L242-244 del	NITRFQTLHISYLTPGDSSS

Spike	K417N	VRQIAPGQTGNIADYNYKLP
Spike	E484K	AGSTPCNGVKGFNCYFPLQS
Spike	N501Y	YFPLQSYGFQPTYGVGYQPY
Spike	D614G	SNQVAVLYQDVNCTEVPVAI
Spike	A701V	SIIAYTMSLGVENSVAYSNN
ORF1ab	T265I	EIKLAKKFDIFNGECPNFVF
ORF1ab	K1655N	RYMSALNHTKNWKYPQVNGL
ORF1ab	H2799Y	TPVHVMSKYTDFSSEIIGYK
ORF1ab	S2900L	AVGNICYTPLKLIEYTDFAT
ORF1ab	K3353R	HSMQNCVLKLRVDTANPKTP
ORF1ab	D4527Y	DLVYALRHFYEGNCDTLKEI
ORF1ab	T5912I	SDRDLYDKLQFISLEIPRRN
ORF3a	Q57H	IVGVALLAVFHSASKIITLK
ORF3a	S171L	NSVTSSIVITLGDGTTSPIS

**Wuhan Hu-1 peptide pool (P.1)**		
N	P80R	GVPINTNSSPDDQIGYYRRA
Spike	L18F and T20N	LPLVSSQCVNLTTRTQLPPA
Spike	P26S	VNLTTRTQLPPAYTNSFTRG
Spike	D138Y	IKVCEFQFCNDPFLGVYYHK
Spike	R190S	GKQGNFKNLREFVFKNIDGY
Spike	K417T	VRQIAPGQTGKIADYNYKLP
Spike	E484K	AGSTPCNGVEGFNCYFPLQS
Spike	N501Y	YFPLQSYGFQPTNGVGYQPY
Spike	D614G	SNQVAVLYQDVNCTEVPVAI
Spike	H655Y	RAGCLIGAEHVNNSYECDIP
Spike	T1027I	IRASANLAATKMSECVLGQS
ORF1ab	S1188L	DKNLYDKLVSSFLEMKSEKQ
ORF1ab	K1795Q	PCTCGKQATKYLVQQESPFV
ORF1ab	E5665D	RIIPARARVECFDKFKVNST
ORF8	E92K	VSCLPFTINCQEPKLGSLVV
**P.1 variant peptide pool**		
N	P80R	GVPINTNSSRDDQIGYYRRA
Spike	L18F and T20N	LPLVSSQCVNFTNRTQLPPA
Spike	P26S	VNFTNRTQLPSAYTNSFTRG
Spike	D138Y	IKVCEFQFCNYPFLGVYYHK
Spike	R190S	GKQGNFKNLSEFVFKNIDGY
Spike	K417T	VRQIAPGQTGTIADYNYKLP
Spike	E484K	AGSTPCNGVKGFNCYFPLQS
Spike	N501Y	YFPLQSYGFQPTYGVGYQPY
Spike	D614G	SNQVAVLYQGVNCTEVPVAI
Spike	H655Y	RAGCLIGAEYVNNSYECDIP
Spike	T1027I	IRASANLAAIKMSECVLGQS
ORF1ab	S1188L	DKNLYDKLVLSFLEMKSEKQ
ORF1ab	K1795Q	PCTCGKQATQYLVQQESPFV
ORF1ab	E5665D	RIIPARARVDCFDKFKVNST
ORF8	E92K	VSCLPFTINCQKPKLGSLVV

Wuhan Hu-1 peptide pool (B.1.617.2)		
Spike	T19R	VSSQCVNLTTRTQLPPAYTN
Spike	EF 156-7 del and R158G	SWMESEFRVYSSANNCTFEY
Spike	T478K	EIYQAGSTPCNGVEGFNCYF
Spike	D614G	SNQVAVLYQDVNCTEVPVAI
Spike	P681R	CASYQTQTNSPRRARSVASQ
Spike	D950N	TASALGKLQDVVNQNAQALN
Spike	(E484Q)	(AGSTPCNGVEGFNCYFPLQS)
Spike	(L425R)	(APGQTGKIADYNYKLPDDFT)
**B.1.617.2 variant peptide pool**		
Spike	T19R	VSSQCVNLRTRTQLPPAYTN
Spike	EF 156-7 del and R158G	SWMESGVYSSANNCTFEYVS
Spike	T478K	EIYQAGSKPCNGVEGFNCYF
Spike	D614G	SNQVAVLYQGVNCTEVPVAI
Spike	P681R	CASYQTQTNSRRRARSVASQ
Spike	D950N	TASALGKLQNVVNQNAQALN
Spike	(E484Q)	(AGSTPCNGVEGFNCYFPLQS)
Spike	(L425R)	(APGQTGKIADYNYKRPDDFT)

Table S3. ***In silico*** **prediction of P.1 VOC peptide binding to common UK HLAII alleles.** NetMHCIIpan epitope predictions for binding of P.1 variant mutated regions to common UK HLA alleles. Weak binding is annotated in blue, strong binding is annotated in Ayellow and the HLA binding core in each instance is shown with the mutated amino acid marked in red. Predicted reduced binding to DRB1*0401 alleles due to mutation N501Y variant sequences is highlighted by a green box.

			NetMHCIIpan predicted HLA binding core with P.1 mutation indicated						
	Mutation	Sequence	DRB1*0101	DRB1*0301	DRB1*0401	DRB1*0701	DRB1*1101	DRB1*1301	DRB1*1501
Nucleocapsid	P80R	Wuhan Hu-1	-	-	-	-	-	-	-
		P.1	-	-	-	-	-	-	-
Spike	L18F	Wuhan Hu-1	-	-	-	-	-	-	-
		P.1	-	-	-	-	-	-	-
	T20N	Wuhan Hu-1	-	-	-	-	-	-	-
		P.1	-	-	-	-	-	-	-
	P26S	Wuhan Hu-1	-	-	-	-	-	-	-
		P.1	-	-	-	-	-	-	-
	D138Y	Wuhan Hu-1	-	-	-	-	-	-	-
		P.1	-	-	-	-	-	-	-
	R190S	Wuhan Hu-1	-	-	-	-	-	-	-
		P.1	-	-	-	-	-	-	-
	K417T	Wuhan Hu-1	-	-	-	-	-	-	-
		P.1	-	TIADYNYKL	-	-	-	-	-
	E484K	Wuhan Hu-1	-	-	-	-	-	-	-
		P.1	-	-	-	-	-	-	-
	N501Y	Wuhan Hu-1	YGFQPTNGV	-	YGFQPTNGV	FQPTNGVGY	-	-	-
		P.1	YGFQPTYGV	-	YGFQPTYGV	FQPTYGVGY	-	-	-
	D614G	Wuhan Hu-1	-	-	-	YQDVNCTEV	-	-	AVLYQDVNC
		P.1	-	-	-	YQGVNCTEV	-	-	AVLYQGVNC
	H655Y	Wuhan Hu-1	-	-	-	-	-	-	-
		P.1	-	-	-	-	-	-	-
	T1027I	Wuhan Hu-1	-	-	-	-	-	-	-
		P.1	-	-	-	-	-	-	-
ORF1a	S1188L	Wuhan Hu-1	YDKLVSSFL	-	-	-	-	-	-
		P.1	-	-	-	-	-	-	-
	K1795Q	Wuhan Hu-1	-	-	-	-	-	-	-
		P.1	-	-	-	-	-	-	-
	E5665D	Wuhan Hu-1	-	-	-	-	-	VECFDKFKV	VECFDKFKV
		P.1	-	-	-	-	-	VDCFDKFKV	-
ORF8	E92K	Wuhan Hu-1	-	-	-	-	-	-	-
		P.1	-	-	-	-	-	-	-

Table S4. ***In silico*** **predictions of B.1.617.2 (AY.4.2) variant peptide binding to common UK HLA class II alleles.** NetMHCIIpan epitope predictions for binding of B.1.617.2 (AY.4.2) variant mutated regions to common UK HLA alleles. Weak binding is annotated blue, strong binding is annotated yellow and the HLA binding core in each instance is shown with the mutated amino acid marked in red.

			NetMHCIIpan predicted HLA binding core with B.1.617.2 / AY.4.2 mutation indicated						
	**Mutation**	**Sequence**	**DRB1*0101**	**DRB1*0301**	**DRB1*0401**	**DRB1*0701**	**DRB1*1101**	**DRB1*1301**	**DRB1*1501**
**Spike**	**T19R**	Wuhan Hu-1	-	-	-	-	-	-	-
		B.1.617.2	-	-	-	-	-	VNLRTRTQL	-
	**Y145H**	Wuhan Hu-1	-	-	VVYHKNNKS	-	YHKNNKSWM	VVYHKNNKS	VVYHKNNKS
		B.1.617.2 (AY.4.2)	-	-	-	-	FLGVYHHKN	LGVYHHKNN	VYHHKNNKS
	**EF156-7 del and R158G**	Wuhan Hu-1	-	-	FRVYSSANN	-	MESEFRVYS	-	FRVYSSANN
		B.1.617.2	-	-	-	-	-	-	-
	**A222V**	Wuhan Hu-1	FSALEPLVD	-	FSALEPLVD	FSALEPLVD	FSALEPLVD	-	-
		B.1.617.2 (AY.4.2)	FSVLEPLVD	-	FSVLEPLVD	-	FSVLEPLVD	-	-
	**L452R**	Wuhan Hu-1	-	-	-	-	YNYLYRLFR	YNYLYRLFR	-
		B.1.617.2	-	-	-	-	YNYRYRLFR	YNYRYRLFR	-
	**T478K**	Wuhan Hu-1	IYQAGSTPC	-	IYQAGSTPC	IYQAGSTPC	-	-	IYQAGSTPC
		B.1.617.2	IYQAGSKPC	-	-	IYQAGSKPC	-	-	IYQAGSKPC
	**D614G**	Wuhan Hu-1	-	-	-	YQDVNCTEV	-	-	AVLYQDVNC
		B.1.617.2	-	-	-	YQGVNCTEV	-	-	AVLYQGVNC
	**P681R**	Wuhan Hu-1	-	-	YQTQTNSPR	-	-	-	-
		B.1.617.2	-	YQTQTNSRR	YQTQTNSRR	-	-	-	-
	**D950N**	Wuhan Hu-1	-	-	LQDVVNQNA	-	-	-	LQDVVNQNA
		B.1.617.2	-	-	LQNVVNQNA	-	-	-	LQNVVNQNA

Table S5. First and second dose COVID-19 vaccine study sub-cohort of HCW with new SARS-CoV-2 infection during the second B.1.1.7 UK wave

	Total	Unvaccinated	One dose vaccinated	Two dose vaccinated
**HCW n (% of total)**	53 (100)	8 (15)	9 (17)	36 (68)
**Mean age (range)**	37 (22-62)	37 (24-48)	29 (22-40)	39 (25-62)
**Gender:**				
**Female n (%)**	39 (74)	7 (88)	6 (67)	26 (72)
**Ethnicity:**				
**White n (%)**	27 (51)	3 (38)	5 (56)	19 (53)
**Minority ethnic group (UK), n (%)**	26 (49)	5 (62)	4 (44)	17 (47)
**BNT162b2 COVID-19 vaccine, n (%)**	41 (77)	0 (0)	5 (56)	36 (100)
**ChAdOx1 nCoV 19 vaccine, n (%)**	4 (8)	0 (0)	4 (44)	0 (0)
**Number of days since second vaccine dose Median (IQR)**			57 (48)	22 (15)

Abbreviations: HCW, Healthcare worker; IQR, inter quartile range. All HCW were newly seropositive for nucleocapsid antibodies. Anti-N Ab expressed as a cut-off index of ≥1.0 were classified as positive . Median date of SARS-CoV-2 PCR positivity 28.12.2020 (IQR of 22 days).

Table S6. Follow up at 55-57 weeks: first and second dose COVID-19 vaccine sub-study with and without laboratory confirmed SARS-CoV-2 infection during the first Wuhan Hu-1 UK wave

		Laboratory confirmed SARS-CoV-2 PCR⁺ and/or Euroimmun S1 Ab⁺ and/or Roche S1 RBD Ab+ and N Ab+ (first UK Wuhan Hu-1 wave)			SARS-CoV-2 PCR⁻ and Euroimmun S1 Ab⁻ Roche S1-RBD Ab- and N Ab-		
	Total	Unvaccinated	One vaccine dose	Two vaccine doses	Unvaccinated	One vaccine dose	Two vaccine doses
**HCW n (% of total)**	305 (100)	7 (2)	9 (3)	47 (15)	11 (4)	10 (3)	221 (73)
**Mean age (range)**	39 (18-69)	32 (21-40)	41 (22-58)	40 (24-62)	33 (18-44)	40 (27-64)	40 (22-69)
**Gender:**							
**Female n (%)**	206 (68)	7 (100)	8 (89)	30 (64)	9 (82)	5 (50)	147 (67)
**Male n (%)**	99 (32)	0 (0)	1 (11)	17 (36)	2 (18)	5 (50)	74 (33)
**Ethnicity:**							
**White n (%)**	221 (72)	7 (100)	6 (67)	34 (72)	10 (91)	7 (70)	157 (71)
**Minority ethnic group (UK) n (%)**	84 (28)	0 (0)	3 (33)	13 (28)	1 (9)	3 (30)	64 (29)
**BNT162b2 COVID-19 vaccine, n (%)**	272 (89)	0 (0)	2 (22)	46 (98)	0 (0)	6 (60)	218 (99)
**Number of days since most recent vaccine dose Median (IQR)**	28 (16)	NA	42 (46)	23 (18)	NA	65 (37)	28 (15)

Abbreviations: HCW, Healthcare worker; NA, Not Applicable

Table S7. Follow up at 71-72 weeks: COVID-19 vaccine study sub-cohort with laboratory confirmed SARS-CoV-2 infection during the first Wuhan Hu-1 UK wave, second B.1.1.7 UK wave and infection naïve.

	Total	SARS-CoV-2 infection naïve<br><br>SARS-CoV-2 PCR$^-$ and Euroimmun S1 Ab$^-$<br>Roche S1-RBD Ab- and N Ab-<br><br>(No evidence of SARS-CoV-2 infection during the first (Wuhan Hu-1) and second (B.1.1.7) UK waves	SARS-CoV-2 infected in first UK Wuhan Hu-1 wave	SARS-CoV-2 infected in second UK B.1.1.7 wave
HCW<br>n (% of total)	80 (100)	27 (34)	31 (39)	22 (27)
Mean age (range)	40<br>(21-62)	40<br>(21-60)	42<br>(27-62)	37<br>(25-57)
Gender:				
Female<br>n (%)	45 (56)	15 (56)	17 (55)	13 (59)
Ethnicity:				
White<br>n (%)	55 (69)	20 (74)	22 (71)	13 (59)
Minority ethnic group (UK), n (%)	25 (31)	7 (26)	9 (29)	9 (41)
BNT162b2 COVID-19 vaccine, n (%)	76 (95)	27 (100)	28 (90)	21 (95)
ChAdOx1 nCoV19 vaccine, n (%)	4 (5)	0 (0)	3 (10)	1 (5)
Number of days since second vaccine dose<br>Median (IQR)	131 (13)	132 (11)	131 (12)	129 (12)

Abbreviations: HCW, Healthcare worker; IQR, inter quartile range.

Table S8. Double vaccinated HCW with possible breakthrough infection at 71-72 weeks follow-up during the B.1.617.2 UK wave.

HCW	1 Infection naïve	2 Prior Wuhan Hu-1 infection	3 Prior B.1.1.7 infection	4 Prior B.1.1.7 infection	5 Prior B.1.1.7 infection	6 Prior B.1.1.7 infection
Age	30	50	33	62	47	34
Sex	Female	Male	Female	Male	Female	Female
N titer 55-57 weeks	0	47.6	178.0	13.4	94.0	1.3
N titer 71-72 weeks	3.7	65.9	233.0	20.0	126.0	18.9
S1 RBD titer 55-57 weeks	46,817	73,779	13,822	19,500	10,068	87,674
S1 RBD titer 71-72 weeks	19,345	43,393	3,488	18,526	4,546	9,206
Days since 1st vaccine dose	208	217	213	217	211	205
Days since 2nd vaccine dose	131	127	133	140	134	128
Days since Wuhan Hu-1 infection	NA	>457	NA	NA	NA	NA
Days since B.1.1.7 infection	NA	NA	>96	>120	190	128

Abbreviations: HCW, health care worker; N, nucleocapsid; S1, Spike subunit 1; RBD, receptor binding domain. Anti-N Ab expressed as a cut-off index of ≥1.0 were classified as positive. HCW with a newly positive anti-N serology result were deemed newly infected. HCW with an increase in anti-N serology were deemed to have been re-infected.

Table S9. Follow up at 83-84 weeks: COVID-19 vaccine study sub-cohort with laboratory confirmed SARS-CoV-2 infection during the first Wuhan Hu-1 UK wave, second B.1.1.7 UK wave and infection naïve.

	Total	SARS-CoV-2 infection naïve<br><br>SARS-CoV-2 PCR⁻ and Euroimmun S1 Ab⁻<br>Roche S1-RBD Ab- and N Ab-<br><br>(No evidence of SARS-CoV-2 infection during the first (Wuhan Hu-1) and second (B.1.1.7) UK waves	SARS-CoV-2 infected in first UK Wuhan Hu-1 wave	SARS-CoV-2 infected in second UK B.1.1.7 wave
**HCW n (% of total)**	74 (100)	35 (47)	20 (27)	19 (26)
**Mean age (range)**	43 (22-63)	46 (22-61)	45 (26-63)	41 (22-63)
**Gender:**				
**Female n (%)**	45 (61)	25 (71)	8 (40)	12 (63)
**Ethnicity:**				
**White n (%)**	53 (72)	26 (74)	15 (75)	12 (63)
**Minority ethnic group (UK), n (%)**	21 (28)	9 (26)	5 (25)	7 (37)
**BNT162b2 COVID-19 vaccine, n (%)**	74 (100)	35 (100)	20 (100)	19 (100)
**BNT162b2 COVID-19 vaccine 3 dose vaccinated, n (%)**	67 (91)	30 (86)	19 (95)	18 (95)
**Number of days since second vaccine dose Median (IQR)**	220 (15)	222 (13)	219 (16)	219 (22)
**Number of days since third vaccine dose Median (IQR)**	18 (12)	18 (11)	20 (13)	18 (17)

Abbreviations: HCW, Healthcare worker; IQR, inter quartile range.

Table S10. HCW with breakthrough infection at 83-84 weeks follow-up during the B.1.617.2 UK wave

HCW	1 Infection naive	2 Infection naive	3 Infection naive	4 Infection naive	5 Infection naive	6 Infection naive	7 Infection naive	8 Infection naive	9 Infection naive	10 Prior Wuhan Hu-1 infection	11 Prior Wuhan Hu-1 infection	12 Prior Wuhan Hu-1 infection	13 Prior B.1.1.7 infection	14 Prior B.1.1.7 infection
Age (y)	55	43	21	50	35	27	31	47	38	58	62	59	27	36
Sex	Female	Female	Female	Female	Male	Male	Female	Female	Female	Male	Female	Male	Female	Male
PCR positive test during B.1.617.2 wave	Yes	Yes			Yes	Yes	Yes	Yes	Yes			Yes		
N titer at 16-18 weeks	-	0	0	0	0	0	-	0	0	4.5	11.8	-	0	0
N titer at 28-30 weeks	0	0	0	0	0	0	0	0	0	10.4	10.2	73	0	0
N titer at 54/55-57 weeks	0	0	0	0	0	0	-	0	0	9.8	6.3	10.1	6.8	56
N titer at 71-72 weeks	-	0	0	0	0	0	-	-	-	6.0	3.5	-	-	-
N titer at 83-84 weeks	4.47	10.5	1.34	8.49	2.49	4.80	1.31	15.1	48.3	83.3	19.9	7.3	97.3	87.9
S1 RBD titer at 54/55-57 weeks	4081	2950	0	12133	16600	9440	-	29308	1110	88200	13800	9010	16001	4316
S1 RBD titer at 71-72 weeks	-	714	17958	3219	5041	1413	-	-	-	17184	3344	-	-	-
S1 RBD titer at 83-84 weeks	20143	35965	38953	32173	20622	9181	29370	67566	88804	39370	11011	9555	51891	19236
Days since 1st vaccine dose	303	313	183	305	300	293	285	249	314	287	297	310	272	239
Days since 2nd vaccine dose	224	236	123	222	220	228	216	192	286	217	217	233	215	211
Days since 3rd vaccine dose	11	14		17		14	14				12	96	22	9
Days since Wuhan Hu-1 infection	NA	NA	NA	NA	NA	NA	NA	NA	NA	566	565	578	NA	NA
Days since B.1.1.7 infection	NA	NA	NA	NA	NA	NA	NA	NA	NA	NA	NA	NA	313	295
Days since PCR confirmed B.1.617.2 infection	78	58	-	-	26	83	88	20	63	-	-	101	-	-

Abbreviations: HCW, health care worker; N, nucleocapsid; S1, Spike subunit 1; RBD, receptor binding domain. Anti-N Ab expressed as a cut-off index of ≥1.0 were classified as positive. HCW with a newly positive ant-N serology result or a positive SARS-CoV-2 PCR test were deemed newly infected. HCW with an increase in anti-N serology were deemed to have been re-infected.

Table S11. Spike mutations in VOC used in this study. Mutations in the receptor binding domain (RBD) are highlighted in grey

Lineage	Spike Mutations (Mutations in RBD)
Wuhan Hu-1	S247R
B.1.1.7	H146H/R, N501Y, A570D, D614G, S982A, H69del, V70del, Y145del, P681H, T716I, D1118H
B.1.351	D80A, D215G, K417N, E484K, N501Y, D614G, A701V, nucleotides 22281-22289
P.1	L18F, T20N, P26S, D138Y, R190S, K417T, E484K, N501Y, D614G, H655Y, T1027I, V1176F
B.1.617.2	T19R, L452R, T478K, D614G, P681R, D950N, nucleotides 22029-22034

Table S12. UK COVIDsortium investigators

The members of the UK COVIDsortium investigators are Hakam Abbass, Aderonke Abiodun, Mashael Alfarih, Zoe Alldis, Daniel M Altmann, Oliver E Amin, Mervyn Andiapen, Jessica Artico, João B Augusto, Georgina L Baca, Sasha N L. Bailey, Anish N Bhuva, Alex Boulter, Ruth Bowles, Rosemary J Boyton, Olivia V Bracken, Ben O'Brien, Tim Brooks, Natalie Bullock, David K Butler, Gabriella Captur, Olivia Carr, Nicola Champion, Carmen Chan, Aneesh Chandran, Tom Coleman, Jorge Couto de Sousa, Xose Couto-Parada, Eleanor Cross, Teresa Cutino-Moguel, Silvia D'Arcangelo, Rhodri H Davies, Brooke Douglas, Cecilia Di Genova, Keenan Dieobi-Anene, Mariana O Diniz, Anaya Ellis, Karen Feehan, Malcolm Finlay, Marianna Fontana, Nasim Forooghi, Joseph M Gibbons, Derek Gilroy, Matt Hamblin, Gabrielle Harker, Jacqueline Hewson, Wendy Heywood, Lauren M Hickling, Bethany Hicks, Aroon D Hingorani, Lee Howes, Ivie Itua, Victor Jardim, Wing-Yiu Jason Lee, Melaniepetra Jensen, Jessica Jones, Meleri Jones, George Joy, Vikas Kapil, Caoimhe Kelly, Hibba Kurdi, Jonathan Lambourne, Kai-Min Lin, Siyi Liu, Sarah Louth, Mala K Maini, Vineela Mandadapu, Charlotte Manisty, Áine McKnight, Katia Menacho, Celina Mfuko, Kevin Mills, Sebastian Millward, Oliver Mitchelmore, Christopher Moon, James Moon, Diana Muñoz Sandoval, Sam M Murray, Mahdad Noursadeghi, Ashley Otter, Corinna Pade, Susana Palma, Ruth Parker, Kush Patel, Mihaela Pawarova, Steffen E Petersen, Brian Piniera, Franziska P Pieper, Lisa Rannigan, Alicja Rapala, Catherine J Reynolds, Amy Richards, Matthew Robathan, Joshua Rosenheim, Cathy Rowe, Jane Sackville West, Genine Sambile, Nathalie M. Schmidt, Amanda Semper, Andreas Seraphim, Mihaela Simion, Angelique Smit, Michelle Sugimoto, Leo Swadling, Stephen Taylor, Nigel Temperton, Stephen Thomas, George D Thornton, Thomas A Treibel, Art Tucker, Ann Varghese, Jessry Veerapen, Mohit Vijayakumar, Tim Warner, Sophie Welch, Theresa Wodehouse, Lucinda Wynne, and Dan Zahedi

Table S13. UK COVIDsortium Immune Correlates Network

The members of the COVIDsortium immune correlates network are Daniel M Altmann, Rosemary J Boyton, Tim Brooks, Benjamin Chain, Mala K Maini, Charlotte Manisty, Áine McKnight, James C Moon, Mahdad Noursadeghi, Thomas A Treibel

References and Notes

1. D. A. Collier, I. A. T. M. Ferreira, P. Kotagiri, R. P. Datir, E. Y. Lim, E. Touizer, B. Meng, A. Abdullahi, CITIID-NIHR BioResource COVID-19 Collaboration, A. Elmer, N. Kingston, B. Graves, E. Le Gresley, D. Caputo, L. Bergamaschi, K. G. C. Smith, J. R. Bradley, L. Ceron-Gutierrez, P. Cortes-Acevedo, G. Barcenas-Morales, M. A. Linterman, L. E. McCoy, C. Davis, E. Thomson, P. A. Lyons, E. McKinney, R. Doffinger, M. Wills, R. K. Gupta, Age-related immune response heterogeneity to SARS-CoV-2 vaccine BNT162b2. *Nature* **596**, 417–422 (2021). doi:10.1038/s41586-021-03739-1 Medline

2. E. C. Wall, M. Wu, R. Harvey, G. Kelly, S. Warchal, C. Sawyer, R. Daniels, P. Hobson, E. Hatipoglu, Y. Ngai, S. Hussain, J. Nicod, R. Goldstone, K. Ambrose, S. Hindmarsh, R. Beale, A. Riddell, S. Gamblin, M. Howell, G. Kassiotis, V. Libri, B. Williams, C. Swanton, S. Gandhi, D. L. Bauer, Neutralising antibody activity against SARS-CoV-2 VOCs B.1.617.2 and B.1.351 by BNT162b2 vaccination. *Lancet* **397**, 2331–2333 (2021). doi:10.1016/S0140-6736(21)01290-3 Medline

3. D. Planas, D. Veyer, A. Baidaliuk, I. Staropoli, F. Guivel-Benhassine, M. M. Rajah, C. Planchais, F. Porrot, N. Robillard, J. Puech, M. Prot, F. Gallais, P. Gantner, A. Velay, J. Le Guen, N. Kassis-Chikhani, D. Edriss, L. Belec, A. Seve, L. Courtellemont, H. Péré, L. Hocqueloux, S. Fafi-Kremer, T. Prazuck, H. Mouquet, T. Bruel, E. Simon-Lorière, F. A. Rey, O. Schwartz, Reduced sensitivity of SARS-CoV-2 variant Delta to antibody neutralization. *Nature* **596**, 276–280 (2021). doi:10.1038/s41586-021-03777-9 Medline

4. W. T. Harvey, A. M. Carabelli, B. Jackson, R. K. Gupta, E. C. Thomson, E. M. Harrison, C. Ludden, R. Reeve, A. Rambaut, S. J. Peacock, D. L. Robertson; COVID-19 Genomics UK (COG-UK) Consortium, SARS-CoV-2 variants, spike mutations and immune escape. *Nat. Rev. Microbiol.* **19**, 409–424 (2021). doi:10.1038/s41579-021-00573-0 Medline

5. T. A. Treibel, C. Manisty, M. Burton, Á. McKnight, J. Lambourne, J. B. Augusto, X. Couto-Parada, T. Cutino-Moguel, M. Noursadeghi, J. C. Moon, COVID-19: PCR screening of asymptomatic health-care workers at London hospital. *Lancet* **395**, 1608–1610 (2020). doi:10.1016/S0140-6736(20)31100-4 Medline

6. C. J. Reynolds, L. Swadling, J. M. Gibbons, C. Pade, M. P. Jensen, M. O. Diniz, N. M. Schmidt, D. K. Butler, O. E. Amin, S. N. L. Bailey, S. M. Murray, F. P. Pieper, S. Taylor, J. Jones, M. Jones, W. J. Lee, J. Rosenheim, A. Chandran, G. Joy, C. Di Genova, N. Temperton, J. Lambourne, T. Cutino-Moguel, M. Andiapen, M. Fontana, A. Smit, A. Semper, B. O'Brien, B. Chain, T. Brooks, C. Manisty, T. Treibel, J. C. Moon, M. Noursadeghi, D. M. Altmann, M. K. Maini, Á. McKnight, R. J. Boyton; COVIDsortium investigators; COVIDsortium immune correlates network, Discordant neutralizing antibody and T cell responses in asymptomatic and mild SARS-CoV-2 infection. *Sci. Immunol.* **5**, eabf3698 (2020). doi:10.1126/sciimmunol.abf3698 Medline

7. C. Manisty, T. A. Treibel, M. Jensen, A. Semper, G. Joy, R. K. Gupta, T. Cutino-Moguel, M. Andiapen, J. Jones, S. Taylor, A. Otter, C. Pade, J. Gibbons, J. Lee, J. Bacon, S. Thomas, C. Moon, M. Jones, D. Williams, J. Lambourne, M. Fontana, D. M. Altmann, R. Boyton, M. Maini, A. McKnight, B. Chain, M. Noursadeghi, J. C. Moon, Time series analysis and mechanistic modelling of heterogeneity and sero-reversion in antibody responses to mild SARS-CoV-2 infection. *EBioMedicine* **65**, 103259 (2021). doi:10.1016/j.ebiom.2021.103259 Medline

8. C. Manisty, A. D. Otter, T. A. Treibel, Á. McKnight, D. M. Altmann, T. Brooks, M. Noursadeghi, R. J. Boyton, A. Semper, J. C. Moon, Antibody response to first BNT162b2 dose in previously SARS-CoV-2-infected individuals. *Lancet* **397**, 1057–1058 (2021). doi:10.1016/S0140-6736(21)00501-8 Medline

9. C. J. Reynolds, C. Pade, J. M. Gibbons, D. K. Butler, A. D. Otter, K. Menacho, M. Fontana, A. Smit, J. E. Sackville-West, T. Cutino-Moguel, M. K. Maini, B. Chain, M. Noursadeghi, ; UK COVIDsortium Immune Correlates Network, T. Brooks, A. Semper, C. Manisty, T. A. Treibel, J. C. Moon, UK COVIDsortium Investigators, A. M. Valdes, Á. McKnight, D. M. Altmann, R. Boyton, Prior SARS-CoV-2 infection rescues B and T cell responses to variants after first vaccine dose. *Science* **372**, eabh1282 (2021). doi:10.1126/science.abh1282 Medline

10. R. K. Gupta, J. Rosenheim, L. C. Bell, A. Chandran, J. A. Guerra-Assuncao, G. Pollara, M. Whelan, J. Artico, G. Joy, H. Kurdi, D. M. Altmann, R. J. Boyton, M. K. Maini, A. McKnight, J. Lambourne, T. Cutino-Moguel, C. Manisty, T. A. Treibel, J. C. Moon, B. M. Chain, M. Noursadeghi, COVIDsortium Investigators, Blood transcriptional biomarkers of acute viral infection for detection of pre-symptomatic SARS-CoV-2 infection: A nested, case-control diagnostic accuracy study. *Lancet Microbe* **2**, e508–e517 (2021). doi:10.1016/S2666-5247(21)00146-4 Medline

11. F. Krammer, K. Srivastava, H. Alshammary, A. A. Amoako, M. H. Awawda, K. F. Beach, M. C. Bermúdez-González, D. A. Bielak, J. M. Carreño, R. L. Chernet, L. Q. Eaker, E. D. Ferreri, D. L. Floda, C. R. Gleason, J. Z. Hamburger, K. Jiang, G. Kleiner, D. Jurczyszak, J. C. Matthews, W. A. Mendez, I. Nabeel, L. C. F. Mulder, A. J. Raskin, K. T. Russo, A. T. Salimbangon, M. Saksena, A. S. Shin, G. Singh, L. A. Sominsky, D. Stadlbauer, A. Wajnberg, V. Simon, Antibody responses in seropositive persons after a single dose of SARS-CoV-2 mRNA vaccine. *N. Engl. J. Med.* **384**, 1372–1374 (2021). doi:10.1056/NEJMc2101667 Medline

12. L. Stamatatos, J. Czartoski, Y. H. Wan, L. J. Homad, V. Rubin, H. Glantz, M. Neradilek, E. Seydoux, M. F. Jennewein, A. J. MacCamy, J. Feng, G. Mize, S. C. De Rosa, A. Finzi, M. P. Lemos, K. W. Cohen, Z. Moodie, M. J. McElrath, A. T. McGuire, mRNA vaccination boosts cross-variant neutralizing antibodies elicited by SARS-CoV-2 infection. *Science* **372**, eabg9175 (2021). doi:10.1126/science.abg9175 Medline

13. R. R. Goel, S. A. Apostolidis, M. M. Painter, D. Mathew, A. Pattekar, O. Kuthuru, S. Gouma, P. Hicks, W. Meng, A. M. Rosenfeld, S. Dysinger, K. A. Lundgreen, L. Kuri-Cervantes, S. Adamski, A. Hicks, S. Korte, D. A. Oldridge, A. E. Baxter, J. R. Giles, M. E. Weirick, C. M. McAllister, J. Dougherty, S. Long, K. D'Andrea, J. T. Hamilton, M. R. Betts, E. T. Luning Prak, P. Bates, S. E. Hensley, A. R. Greenplate, E. J. Wherry, Distinct antibody and memory B cell responses in SARS-CoV-2 naïve and recovered individuals after mRNA vaccination. *Sci. Immunol.* **6**, eabi6950 (2021). doi:10.1126/sciimmunol.abi6950 Medline

14. J. E. Ebinger, J. Fert-Bober, I. Printsev, M. Wu, N. Sun, J. C. Prostko, E. C. Frias, J. L. Stewart, J. E. Van Eyk, J. G. Braun, S. Cheng, K. Sobhani, Antibody responses to the BNT162b2 mRNA vaccine in individuals previously infected with SARS-CoV-2. *Nat. Med.* **27**, 981–984 (2021). doi:10.1038/s41591-021-01325-6 Medline

15. P. S. Arunachalam, M. K. D. Scott, T. Hagan, C. Li, Y. Feng, F. Wimmers, L. Grigoryan, M. Trisal, V. V. Edara, L. Lai, S. E. Chang, A. Feng, S. Dhingra, M. Shah, A. S. Lee, S. Chinthrajah, S. B. Sindher, V. Mallajosyula, F. Gao, N. Sigal, S. Kowli, S. Gupta, K. Pellegrini, G. Tharp, S. Maysel-Auslender, S. Hamilton, H. Aoued, K. Hrusovsky, M. Roskey, S. E. Bosinger, H. T. Maecker, S. D. Boyd, M. M. Davis, P. J. Utz, M. S. Suthar, P. Khatri, K. C. Nadeau, B. Pulendran, Systems vaccinology of the BNT162b2 mRNA vaccine in humans. *Nature* **596**, 410–416 (2021). doi:10.1038/s41586-021-03791-x Medline

16. A. Zhang, H. D. Stacey, C. E. Mullarkey, M. S. Miller, Original antigenic sin: How first exposure shapes lifelong anti-influenza virus immune responses. *J. Immunol.* **202**, 335–340 (2019). doi:10.4049/jimmunol.1801149 Medline

17. T. Aydillo, A. Rombauts, D. Stadlbauer, S. Aslam, G. Abelenda-Alonso, A. Escalera, F. Amanat, K. Jiang, F. Krammer, J. Carratala, A. García-Sastre, Immunological imprinting of the antibody response in COVID-19 patients. *Nat. Commun.* **12**, 3781 (2021). doi:10.1038/s41467-021-23977-1 Medline

18. A. K. Wheatley, A. Fox, H. X. Tan, J. A. Juno, M. P. Davenport, K. Subbarao, S. J. Kent, Immune imprinting and SARS-CoV-2 vaccine design. *Trends Immunol.* **42**, 956–959 (2021). doi:10.1016/j.it.2021.09.001 Medline

19. N. Kamar, F. Abravanel, O. Marion, C. Couat, J. Izopet, A. Del Bello, Three doses of an mRNA Covid-19 vaccine in solid-organ transplant recipients. *N. Engl. J. Med.* **385**, 661–662 (2021). doi:10.1056/NEJMc2108861 Medline

20. A. Del Bello, F. Abravanel, O. Marion, C. Couat, L. Esposito, L. Lavayssière, J. Izopet, N. Kamar, Efficiency of a boost with a third dose of anti-SARS-CoV-2 messenger RNA-based vaccines in solid organ transplant recipients. *Am. J. Transplant.* ajt.16775 (2021). doi:10.1111/ajt.16775 Medline

21. D. M. Altmann, R. J. Boyton, R. Beale, Immunity to SARS-CoV-2 variants of concern. *Science* **371**, 1103–1104 (2021). doi:10.1126/science.abg7404 Medline

22. D. S. Khoury, D. Cromer, A. Reynaldi, T. E. Schlub, A. K. Wheatley, J. A. Juno, K. Subbarao, S. J. Kent, J. A. Triccas, M. P. Davenport, Neutralizing antibody levels are highly predictive of immune protection from symptomatic SARS-CoV-2 infection. *Nat. Med.* **27**, 1205–1211 (2021). doi:10.1038/s41591-021-01377-8 Medline

23. A. M. Solinger, M. E. Ultee, E. Margoliash, R. H. Schwartz, T-lymphocyte response to cytochrome c. I. Demonstration of a T-cell heteroclitic proliferative response and identification of a topographic antigenic determinant on pigeon cytochrome c whose immune recognition requires two complementing major histocompatibility complex-linked immune response genes. *J. Exp. Med.* **150**, 830–848 (1979). doi:10.1084/jem.150.4.830 Medline

24. D. P. Martin, S. Weaver, H. Tegally, J. E. San, S. D. Shank, E. Wilkinson, A. G. Lucaci, J. Giandhari, S. Naidoo, Y. Pillay, L. Singh, R. J. Lessells, R. K. Gupta, J. O. Wertheim, A. Nekturenko, B. Murrell, G. W. Harkins, P. Lemey, O. A. MacLean, D. L. Robertson, T. de Oliveira, S. L. Kosakovsky Pond; NGS-SA; COVID-19 Genomics UK (COG-UK), The emergence and ongoing convergent evolution of the SARS-CoV-2 N501Y lineages. *Cell* **184**, 5189–5200.e7 (2021). doi:10.1016/j.cell.2021.09.003 Medline

25. L. Lu, A. W. Chu, R. R. Zhang, W. M. Chan, J. D. Ip, H. W. Tsoi, L. L. Chen, J. P. Cai, D. C. Lung, A. R. Tam, Y. S. Yau, M. Y. Kwan, W. K. To, O. T. Tsang, L. L. Lee, H. Yi, T. C. Ip, R. W. Poon, G. K. Siu, B. W. Mok, V. C. Cheng, K. H. Chan, K. Y. Yuen, I. F. Hung, K. K. To, The impact of spike N501Y mutation on neutralizing activity and RBD binding of SARS-CoV-2 convalescent serum. *EBioMedicine* **71**, 103544 (2021). doi:10.1016/j.ebiom.2021.103544 Medline

26. GISAID, "Tracking of variants" (2021); https://www.gisaid.org/hcov19-variants/

27. T. M. Snyder, R. M. Gittelman, M. Klinger, D. H. May, E. J. Osborne, R. Taniguchi, H. J. Zahid, I. M. Kaplan, J. N. Dines, M. T. Noakes, R. Pandya, X. Chen, S. Elasady, E. Svejnoha, P. Ebert, M. W. Pesesky, P. De Almeida, H. O'Donnell, Q. DeGottardi, G. Keitany, J. Lu, A. Vong, R. Elyanow, P. Fields, J. Greissl, L. Baldo, S. Semprini, C. Cerchione, F. Nicolini, M. Mazza, O. M. Delmonte, K. Dobbs, R. Laguna-Goya, G. Carreño-Tarragona, S. Barrio, L. Imberti, A. Sottini, E. Quiros-Roldan, C. Rossi, A. Biondi, L. R. Bettini, M. D'Angio, P. Bonfanti, M. F. Tompkins, C. Alba, C. Dalgard, V. Sambri, G. Martinelli, J. D. Goldman, J. R. Heath, H. C. Su, L. D. Notarangelo, E. Paz-Artal, J. Martinez-Lopez, J. M. Carlson, H. S. Robins, Magnitude and dynamics of the T-cell response to SARS-CoV-2 Infection at both individual and population levels. medRxiv 2020.07.31.20165647 [Preprint] (2020); https://doi.org/10.1101/2020.07.31.20165647.

28. Wellcome Sanger Institute, "COVID-19 genomic surveillance" (2021); https://covid19.sanger.ac.uk/lineages/raw.

29. F. Muecksch, Y. Weisblum, C. O. Barnes, F. Schmidt, D. Schaefer-Babajew, Z. Wang, J. C. C. Lorenzi, A. I. Flyak, A. T. DeLaitsch, K. E. Huey-Tubman, S. Hou, C. A. Schiffer, C. Gaebler, J. Da Silva, D. Poston, S. Finkin, A. Cho, M. Cipolla, T. Y. Oliveira, K. G. Millard, V. Ramos, A. Gazumyan, M. Rutkowska, M. Caskey, M. C. Nussenzweig, P. J. Bjorkman, T. Hatziioannou, P. D. Bieniasz, Affinity maturation of SARS-CoV-2 neutralizing antibodies confers potency, breadth, and resilience to viral escape mutations. *Immunity* **54**, 1853–1868.e7 (2021). doi:10.1016/j.immuni.2021.07.008 Medline

30. Z. Wang, F. Muecksch, D. Schaefer-Babajew, S. Finkin, C. Viant, C. Gaebler, H. H. Hoffmann, C. O. Barnes, M. Cipolla, V. Ramos, T. Y. Oliveira, A. Cho, F. Schmidt, J. Da Silva, E. Bednarski, L. Aguado, J. Yee, M. Daga, M. Turroja, K. G. Millard, M. Jankovic, A. Gazumyan, Z. Zhao, C. M. Rice, P. D. Bieniasz, M. Caskey, T. Hatziioannou, M. C. Nussenzweig, Naturally enhanced neutralizing breadth against SARS-CoV-2 one year after infection. *Nature* **595**, 426–431 (2021). doi:10.1038/s41586-021-03696-9 Medline

31. C. Gaebler, Z. Wang, J. C. C. Lorenzi, F. Muecksch, S. Finkin, M. Tokuyama, A. Cho, M. Jankovic, D. Schaefer-Babajew, T. Y. Oliveira, M. Cipolla, C. Viant, C. O. Barnes, Y. Bram, G. Breton, T. Hägglöf, P. Mendoza, A. Hurley, M. Turroja, K. Gordon, K. G. Millard, V. Ramos, F. Schmidt, Y. Weisblum, D. Jha, M. Tankelevich, G. Martinez-Delgado, J. Yee, R. Patel, J. Dizon, C. Unson-O'Brien, I. Shimeliovich, D. F. Robbiani, Z. Zhao, A. Gazumyan, R. E. Schwartz, T. Hatziioannou, P. J. Bjorkman, S. Mehandru, P. D. Bieniasz, M. Caskey, M. C. Nussenzweig, Evolution of antibody immunity to SARS-CoV-2. *Nature* **591**, 639–644 (2021). doi:10.1038/s41586-021-03207-w Medline

32. N. Faulkner, K. W. Ng, M. Y. Wu, R. Harvey, M. Margaritis, S. Paraskevopoulou, C. Houlihan, S. Hussain, M. Greco, W. Bolland, S. Warchal, J. Heaney, H. Rickman, M. Spyer, D. Frampton, M. Byott, T. de Oliveira, A. Sigal, S. Kjaer, C. Swanton, S. Gandhi, R. Beale, S. J. Gamblin, J. W. McCauley, R. S. Daniels, M. Howell, D. Bauer, E. Nastouli, G. Kassiotis, Reduced antibody cross-reactivity following infection with B.1.1.7 than with parental SARS-CoV-2 strains. *eLife* **10**, e69317 (2021). doi:10.7554/eLife.69317 Medline

33. M. Bergwerk, T. Gonen, Y. Lustig, S. Amit, M. Lipsitch, C. Cohen, M. Mandelboim, E. G. Levin, C. Rubin, V. Indenbaum, I. Tal, M. Zavitan, N. Zuckerman, A. Bar-Chaim, Y. Kreiss, G. Regev-Yochay, Covid-19 breakthrough infections in vaccinated health care workers. *N. Engl. J. Med.* **385**, 1474–1484 (2021). doi:10.1056/NEJMoa2109072 Medline

34. T. Moyo-Gwete, M. Madzivhandila, Z. Makhado, F. Ayres, D. Mhlanga, B. Oosthuysen, B. E. Lambson, P. Kgagudi, H. Tegally, A. Iranzadeh, D. Doolabh, L. Tyers, L. R. Chinhoyi, M. Mennen, S. Skelem, G. Marais, C. K. Wibmer, J. N. Bhiman, V. Ueckermann, T. Rossouw, M. Boswell, T. de Oliveira, C. Williamson, W. A. Burgers, N. Ntusi, L. Morris, P. L. Moore, Cross-reactive neutralizing antibody responses elicited by SARS-CoV-2 501Y.V2 (B.1.351). *N. Engl. J. Med.* **384**, 2161–2163 (2021). doi:10.1056/NEJMc2104192 Medline

35. M. McCallum, A. De Marco, F. A. Lempp, M. A. Tortorici, D. Pinto, A. C. Walls, M. Beltramello, A. Chen, Z. Liu, F. Zatta, S. Zepeda, J. di Iulio, J. E. Bowen, M. Montiel-Ruiz, J. Zhou, L. E. Rosen, S. Bianchi, B. Guarino, C. S. Fregni, R. Abdelnabi, S. C. Foo, P. W. Rothlauf, L. M. Bloyet, F. Benigni, E. Cameroni, J. Neyts, A. Riva, G. Snell, A. Telenti, S. P. J. Whelan, H. W. Virgin, D. Corti, M. S. Pizzuto, D. Veesler, N-terminal domain antigenic mapping reveals a site of vulnerability for SARS-CoV-2. *Cell* **184**, 2332–2347.e16 (2021). doi:10.1016/j.cell.2021.03.028 Medline

36. Y. Goldberg, M. Mandel, Y. M. Bar-On, O. Bodenheimer, L. Freedman, E. J. Haas, R. Milo, S. Alroy-Preis, N. Ash, A. Huppert, Waning immunity after the BNT162b2 vaccine in Israel. *N. Engl. J. Med.* NEJMoa2114228 (2021). doi:10.1056/NEJMoa2114228 Medline

37. L. A. VanBlargan, L. J. Adams, Z. Liu, R. E. Chen, P. Gilchuk, S. Raju, B. K. Smith, H. Zhao, J. B. Case, E. S. Winkler, B. M. Whitener, L. Droit, I. D. Aziati, T. L. Bricker, A. Joshi, P. Y. Shi, A. Creanga, A. Pegu, S. A. Handley, D. Wang, A. C. M. Boon, J. E. Crowe Jr., S. P. J. Whelan, D. H. Fremont, M. S. Diamond, A potently neutralizing SARS-CoV-2 antibody inhibits variants of concern by utilizing unique binding residues in a highly conserved epitope. *Immunity* **54**, 2399–2416.e6 (2021). doi:10.1016/j.immuni.2021.08.016 Medline

38. D. M. Altmann, C. J. Reynolds, R. J. Boyton, SARS-CoV-2 variants: Subversion of antibody response and predicted impact on T cell recognition. *Cell Rep. Med.* **2**, 100286 (2021). doi:10.1016/j.xcrm.2021.100286 Medline

39. B. Reynisson, B. Alvarez, S. Paul, B. Peters, M. Nielsen, NetMHCpan-4.1 and NetMHCIIpan-4.0: Improved predictions of MHC antigen presentation by concurrent motif deconvolution and integration of MS MHC eluted ligand data. *Nucleic Acids Res.* **48** (W1), W449–W454 (2020). doi:10.1093/nar/gkaa379 Medline

40. K. J. Quigley, C. J. Reynolds, A. Goudet, E. J. Raynsford, R. Sergeant, A. Quigley, S. Worgall, D. Bilton, R. Wilson, M. R. Loebinger, B. Maillere, D. M. Altmann, R. J. Boyton, Chronic infection by mucoid *Pseudomonas aeruginosa* associated with dysregulation in T-cell immunity to outer membrane porin F. *Am. J. Respir. Crit. Care Med.* **191**, 1250–1264 (2015). doi:10.1164/rccm.201411-1995OC Medline

41. C. Reynolds, A. Goudet, K. Jenjaroen, M. Sumonwiriya, D. Rinchai, J. Musson, S. Overbeek, J. Makinde, K. Quigley, J. Manji, N. Spink, P. Yos, V. Wuthiekanun, G. Bancroft, J. Robinson, G. Lertmemongkolchai, S. Dunachie, B. Maillere, M. Holden, D. Altmann, R. Boyton, T cell immunity to the alkyl hydroperoxide reductase of *Burkholderia pseudomallei*: A correlate of disease outcome in acute melioidosis. *J. Immunol.* **194**, 4814–4824 (2015). doi:10.4049/jimmunol.1402862 Medline

42. K. Tao, P. L. Tzou, J. Nouhin, R. K. Gupta, T. de Oliveira, S. L. Kosakovsky Pond, D. Fera, R. W. Shafer, The biological and clinical significance of emerging SARS-CoV-2 variants. *Nat. Rev. Genet.* **22**, 757–773 (2021). doi:10.1038/s41576-021-00408-x Medline

43. J. Zou, X. Xie, C. R. Fontes-Garfias, K. A. Swanson, I. Kanevsky, K. Tompkins, M. Cutler, D. Cooper, P. R. Dormitzer, P. Y. Shi, The effect of SARS-CoV-2 D614G mutation on BNT162b2 vaccine-elicited neutralization. *NPJ Vaccines* **6**, 44 (2021). doi:10.1038/s41541-021-00313-8 Medline
