# Supplementary material for: Heterologous infection and vaccination shapes immunity against SARS-CoV-2 variants
Source: Science. 2021 Dec 2;375(6577):183–92. doi: 10.1126/science.abm0811 (PMC10186585; doi:10.1126/science.abm0811)
Supplement: Supplementary file 4 — MDAR Reproducibility Checklist [file science.abm0811_mdar_reproducibility_checklist.pdf]

Materials Design Analysis Reporting (MDAR) Checklist for Authors

The MDAR framework establishes a minimum set of requirements in transparent reporting applicable to studies in the life sciences (see Statement of Task: doi:10.31222/osf.io/9sm4x.). The MDAR checklist is a tool for authors, editors and others seeking to adopt the MDAR framework for transparent reporting in manuscripts and other outputs. Please refer to the MDAR Elaboration Document for additional context for the MDAR framework.

Materials

Antibodies	Yes (indicate where provided: page no/section/legend)	n/a
For commercial reagents, provide supplier name, catalogue number and RRID, if available.	All antibodies were supplied by Mabtech, Sweden.<br>T cell ELISpot antibodies<br>ELISpot plates pre-coated with anti-human IFNg mAb 1-D1K.<br>ELISpot alkaline phosphatase conjugated detection antibody, anti-human IFNg mAb 7-B6-1-ALP<br>ELIspot positive control anti-CD3 mAb CD3-2<br>B cell ELISpot antibodies<br>Coating antibody for total IgG wells was anti-Human IgG mAbs MT91/145<br>Biotinylated secondary antibody was anti-Human mAbs MT78/145<br>Flow cytometry antibodies<br>Anti-mouse CD3 FITC, BD Pharmingen, clone 145-2C11, product code 553062<br>Anti-mouse CD4 APC/Cy7, Biolegend, clone GK1.5, product code 100414<br>Anti-mouse CD8 AlexaFluor®647, Biolegend, clone 53-6.7, product code 100724<br>Anti-mouse Foxp3 AlexaFluor®700, eBioscience, clone FJK-16S, product code 56-5773-80<br>Biotin Mouse Anti-Human IgG, BD Pharmingen, clone G18-145<br>This information is located in the Supplementary Materials, Materials and Methods section.	

Cell materials	Yes (indicate where provided: page no/section/legend)	n/a
**Cell lines:** Provide species information, strain. Provide accession number in repository **OR** supplier name, catalog number, clone number, **OR** RRID	VeroE6 (ATCC, The Global Bioresource Centre) [https://www.lgcstandards-atcc.org/]<br>This information is located in the Supplementary Materials, Materials and Methods section.	
**Primary cultures:** Provide species, strain, sex of origin, genetic modification status.		n/a

Experimental animals	Yes (indicate where provided: page no/section/legend)	n/a
**Laboratory animals:** Provide species, strain, sex, age, genetic modification status. Provide accession number in repository **OR** supplier name, catalog number, clone number, **OR** RRID	Mouse, HLA DRB1*0401, female, 8-10 weeks of age. Mice carry transgene for the human HLA allele DRB1*0401 and are homozygous knockout for murine H2-Abeta. This information is located in the Supplementary Materials, Materials and Methods section.	
**Animal observed in or captured from the field:** Provide species, sex and age where possible		n/a
**Model organisms:** Provide Accession number in repository (where relevant) **OR** RRID		n/a

Plants and microbes	Yes (indicate where provided: page no/section/legend)	n/a
**Plants:** provide species and strain, unique accession number if available, and source (including location for collected wild specimens)		n/a

**Microbes:** provide species and strain, unique accession number if available, and source	SARS-CoV-2 virus isolates were obtained from either the European Virus Archive Global (EVAg) or Centre for AIDS reagents (CFAR) repository at The National Institute for Biological Standards and Controls (NIBSC).<br>SARS-CoV-2 strain 2019-nCoV/BavPat1/2020 (Wuhan Hu-1) was obtained from EVAg (Ref-SKU: 026V-03883).<br>The SARS-CoV-2 VOC202012/01 B.1.1.7 isolate was obtained from CFAR (CFAR#101019).<br>The nCoV19 isolate/UK ex South African/2021 lineage B.1.351 was obtained from EVAg (Ref-SKU: 004V-04071).<br>The nCov19 hCoV-19/Netherlands/NoordHolland_10915/2021, Brazilian variant P.1, nextstrain clade 20J, lineage B.1.1.28 P.1 was obtained from EVAg (Ref-SKU: 014V-04089).<br>The nCoV19 isolate/Germany ex India, 2021, 20A/452R (B.1.617) was obtained from EVA (Ref-SKU: 009V-04187).<br>This information is located in the Supplementary Materials, Materials and Methods section, Acknowledgements and Data and Material Availability statement.	

Human research participants	Yes (indicate where provided: page no/section/legend)	n/a
Identify authority granting ethics approval (IRB or equivalent committee(s), provide reference number for approval.	The COVIDsortium Healthcare Workers bioresource was approved by the ethical committee of UK National Research Ethics Service (20/SC/0149) and registered on ClinicalTrials.gov (NCT04318314). The study conformed to the principles of the Helsinki Declaration, and all subjects gave written informed consent. This information is located in the Supplementary Materials, Materials and Methods section and Acknowledgements.	
Provide statement confirming informed consent obtained from study participants.	All subjects gave written informed consent	

Report on age and sex for all study participants.	Adult Healthcare workers HCW (>18 years old) self-declared as fit to attend work. Mean age of the COVIDsortium cohort (n=731) was 38±11 years; 33% male. The cross-sectional vaccine sub-study reported here included 25 vaccinated HCW (mean age 44y, 60% male) with laboratory defined SARS-CoV-2 either by SARS-CoV-2 positive PCR and/or positive spike IgG (Euroimmun ELISA) / NP IgG/IgM antibody (Roche Elecsys). A second group of 26 vaccinated HCW (mean age 41y, 54% male) with no evidence of prior SARS-CoV-2 infection were also recruited. All were SARS-CoV-2 PCR negative and negative for Spike IgG (Euroimmun ELISA) and NP IgG/IgM antibody (Roche Elecsys) tests at baseline and throughout the first 16-weekly follow-up. Fig. S1, Table S1. A total of n=358 HCW were studied at 55-57-week follow-up (mean age 39y, 32% male). 63 had laboratory confirmed SARS-CoV-2 infection (Wuhan Hu-1) during the first wave and 242 were infection naïve. Fig, S1, Table S5/S6. A total of 53 were infected by B.1.1.7 during the second wave (mean age 37y, 61% male) Fig. S1, Table S5. A total of 80 HCW were studied at 71-72-week follow-up (mean age 40y, 55% male). Of these, n=27 had no laboratory evidence of SARS-CoV-2 infection, n=31 had prior Wuhan Hu-1 SARS-CoV-2 infection (during the first wave) and n=22 had B.1.1.7 SARS-CoV-2 infection (during the second wave) Fig. S1, Table S7. A total of 59 HCW were studied at 83-84-week follow-up (mean age 44y, 36% male). Of these, n=30 had no laboratory evidence of SARS-CoV-2 infection, n=15 had prior Wuhan Hu-1 SARS-CoV-2 infection (during the first wave) and n=14 had B.1.1.7 SARS-CoV-2 infection (during the second wave) Fig. S1, Table S.9<br><br>This information is located in the Supplementary Materials, Materials and Methods section, Fig S1 and Table S1, Table S5, Table S6, Table S7, Table S9.	

Design

**Study protocol**	**Yes (indicate where provided: page no/section/legend)**	**n/a**
For clinical trials, provide the trial registration number **OR** cite DOI in manuscript.	ClinicalTrials.gov (NCT04318314). This information is located in the Supplementary Materials, Materials and Methods and Acknowledgements.	

**Laboratory protocol**	**Yes (indicate where provided: page no/section/legend)**	**n/a**
Provide DOI or other citation details if detailed step-by-step protocols are available.	This information is located in the Supplementary Materials, Materials and Methods which includes a detailed description of each experimental laboratory protocol used in the study. References supporting the protocols are also included.	

**Experimental study design (statistics details)**	**Yes (indicate where provided: page no/section/legend)**	**n/a**
State whether and how the following have been done**, or** if they were not carried out.		

Sample size determination	The cross-sectional vaccine sub-study cohorts reported here included 25 vaccinated HCW (mean age 44y, 60% male) with laboratory evidence of SARS-CoV-2 either by SARS-CoV-2 positive PCR and/or positive spike IgG (Euroimmun ELISA) / NP IgG/IgM antibody (Roche Elecsys). A second group of 26 vaccinated HCW (mean age 41y, 54% male) with no evidence of previous SARS-CoV-2 infection were also recruited. All were SARS-CoV-2 PCR negative and negative for Spike IgG (Euroimmun ELISA) and NP IgG/IgM antibody (Roche Elecsys) tests at baseline and throughout all follow ups. Sample size was based on available sample sets following active recruitment at 3 weeks after the first and second vaccine doses.<br>The B.1.1.7 infection 55-57-week follow-up cross-sectional sub-cohort included 53 HCW infected by B.1.1.7 during the second wave (mean age 37y, 61% male); 63 HCW infected by Wuhan Hu-1 during the first wave and 242 uninfected HCW (mean age 39y, 32% male). Sample size was based on available sample sets following active recruitment at 55-57-weeks follow-up.<br>The 71-72-week follow-up cross-sectional sub-cohort reported 80 HCW [n=31 (39%) prior infection by Wuhan Hu-1 during the first wave; n= 22 (27%) prior infection by B.1.1.7 during the second wave and n=27 (34%) with no evidence of SARS-CoV-2 infection during the first and second wave]. Sample size was based on available sample sets following active recruitment at 71-72-weeks follow-up.<br>The 83-84-week follow-up cross-sectional sub-cohort reported 59 HCW [n=15 (25%) prior infection by Wuhan Hu-1 during the first wave; n= 14 (24%) prior infection by B.1.1.7 during the second wave and n=30 (51%) with no evidence of SARS-CoV-2 infection during the first and second wave]. Sample size was based on available sample sets following active recruitment at 83-84-weeks follow-up.<br><br>This information is located in the Supplementary Materials, Materials and Methods, Fig S1 and Table S1, Table S5, Table S6, Table S7, Table S9.	
Randomisation	Randomization was not appropriate for this study as there was no therapeutic intervention.	
Blinding	Blinding was not appropriate for this study as there was no therapeutic intervention. The laboratory staff were blinded to HCW ID when doing experimental work.	
Inclusion/exclusion criteria	Adult Healthcare workers HCW (>18 years old) who self-declared as fit to attend work were invited to participate via local advertisement of the project (see https://covid-consortium.com). This information is located in the Supplementary Materials, Materials and Methods section called, COVIDsortium Healthcare Worker Participants.	

Sample definition and in-laboratory replication	Yes (indicate where provided: page no/section/legend)	n/a
State number of times the experiment was replicated in laboratory	This information is located in the Supplementary Materials, Materials and Methods that describes each *in vitro* experiment. n numbers are stated on Figures and in figure legends. Fig. 1 to Fig. 4 and Fig. S1 to Fig. S11.	

Define whether data describe technical or biological replicates	The number of individuals (either SARS-CoV-2 infected or uninfected), or transgenic mice included in each experiment is described in each figure legend and the Materials and Methods section. Samples analysed in the study were all participants in a HCW cohort study and samples were analysed from individual HCW participants. Experiments did not include replicates as all HCW participants and data points are unique. Some experiments used technical and/or biological replicates. All the experiments shown in Fig. 1 to Fig. 4 and Fig. S2 to S11 were all preformed in technical duplicates. HLA DRB1*0401 transgenic studies (Fig 2F to J) included technical duplicates and biological replicates as indicated in the legend.	

**Ethics**	**Yes (indicate where provided: page no/section/legend)**	**n/a**
Studies involving human participants: State details of authority granting ethics approval (IRB or equivalent committee(s), provide reference number for approval.	The COVIDsortium Healthcare Workers bioresource was approved by the ethical committee of UK National Research Ethics Service (20/SC/0149) and registered on ClinicalTrials.gov (NCT04318314). The study conformed to the principles of the Helsinki Declaration, and all subjects gave written informed consent. This information is located in the Supplementary Materials, Materials and Methods and Acknowledgements.	
Studies involving experimental animals: State details of authority granting ethics approval (IRB or equivalent committee(s), provide reference number for approval.	Mouse experiments were performed following approval by the UK Home Office under the terms of the project licence P809B6A94 granted under the "Animals (Scientific Procedures) Act 1986" This information is located in the Supplementary Materials, Materials and Methods and Acknowledgements.	
Studies involving specimen and field samples: State if relevant permits obtained, provide details of authority approving study; if none were required, explain why.		n/a

**Dual Use Research of Concern (DURC)**	**Yes (indicate where provided: page no/section/legend)**	**n/a**
If study is subject to dual use research of concern, state the authority granting approval and reference number for the regulatory approval		n/a

Analysis

Attrition	Yes (indicate where provided: page no/section/legend)	n/a
State if sample or data point from the analysis is excluded, and whether the criteria for exclusion were determined and specified in advance.	ELISpot results were excluded if negative control wells had >100 SFU/106 PBMC (n=4) or cell viability was low with <1000 SFU/106 PBMC in anti-CD3 positive control wells (n=16). The criteria for exclusion were determined and specified in advance.	n/a

Statistics	Yes (indicate where provided: page no/section/legend)	n/a
Describe statistical tests used and justify choice of tests.	Data was assumed to have a non-Gaussian distribution. Non-parametric tests were used throughout. For single paired and unpaired comparisons Wilcoxon matched-pairs signed rank test and a Mann-Whitney U-test were used. For correlations, Spearman's r test was used. A p value <0.05 was considered significant. Prism 9.0 for Mac was used for analysis. This information is located in the Supplementary Materials, Materials and Methods and Figure legends for Fig. 1 to Fig. 4 and Fig. S2 to Fig. S11.	

Data Availability	Yes (indicate where provided: page no/section/legend)	n/a
State whether newly created datasets are available, including protocols for access or restriction on access.	All data is presented in the manuscript and/or in the Supplementary Material files.	
If data are publicly available, provide accession number in repository or DOI or URL.		n/a
If publicly available data are reused, provide accession number in repository or DOI or URL, where possible.		n/a

Code Availability	Yes (indicate where provided: page no/section/legend)	n/a
For all newly generated code and software essential for replicating the main findings of the study:		
State whether the code or software is available.		n/a
If code is publicly available, provide accession number in repository, or DOI or URL.		n/a

Reporting

Adherence to community standards	Yes (indicate where provided: page no/section/legend)	n/a
MDAR framework recommends adoption of discipline-specific guidelines, established and endorsed through community initiatives. Journals have their own policy about requiring specific guidelines and recommendations to complement MDAR.		
State if relevant guidelines (eg., ICMJE, MIBBI, ARRIVE) have been followed, and whether a checklist (eg., CONSORT, PRISMA, ARRIVE) is provided with the manuscript.	CONSORT flow diagram of UK COVIDsortium healthcare worker cohort and first dose vaccine sub-study is provided in Supplementary Figure S1A	
